# NDST3‐Induced Epigenetic Reprogramming Reverses Neurodegeneration in Parkinson's Disease

**DOI:** 10.1002/advs.202507323

**Published:** 2025-11-21

**Authors:** Yujung Chang, Yongwoo Na, Hyeonjoo Im, Garam Yang, Seungseon Yang, Hyun Soo Shim, Chunggoo Kim, GyeungYun Kim, Hyeok Ju Park, Hee Young Kim, Seung Eun Lee, Wonwoong Lee, Yoon Ha, Sungho Park, Jieun Kim, Won‐Young Cho, Woong Sun, Jong‐Seo Kim, Junsang Yoo

**Affiliations:** ^1^ Laboratory of Regenerative Medicine for Neurodegenerative Disease Stand Up Therapeutics Inc. Hannamdaero 98 Seoul 04418 South Korea; ^2^ Center for RNA Research Institute for Basic Science Seoul 08826 South Korea; ^3^ School of Biological Sciences Seoul National University Seoul 08826 South Korea; ^4^ Department of Biological Sciences Korea Advanced Institute of Science and Technology (KAIST) Daejeon 34141 South Korea; ^5^ Biology Department Boston University Boston MA 02215 USA; ^6^ Database Laboratory Department of Computer Science and Engineering Dongguk University Seoul 04620 South Korea; ^7^ Department of Physiology College of Medicine Yonsei University Seoul 03722 South Korea; ^8^ Research Animal Resource Center Korea Institute of Science and Technology Seoul 02792 South Korea; ^9^ College of Pharmacy Woosuk University Wanju 55338 South Korea; ^10^ Department of Neurosurgery College of Medicine Yonsei University Seoul 03722 South Korea; ^11^ Department of Chemistry Yonsei University Seoul 03722 South Korea; ^12^ Department of Bio‐Health Technology College of Biomedical Science and Multi‐dimensional Genomics Research Center Kangwon National University Chuncheon 24341 South Korea; ^13^ Department of Anatomy College of Medicine Korea University Seoul 02841 South Korea

**Keywords:** cell vitalization, cellular repair, epigenetic reprogramming, neurodegenerative disease, Parkinson's disease

## Abstract

Parkinson's disease (PD) is a progressive neurodegenerative disorder characterized by the loss of dopaminergic neurons (DN) in the substantia nigra and disruption of cellular maintenance. Here, we demonstrate that NDST3‐mediated epigenetic reprogramming halts neuronal degeneration and restores motor function in an animal model of PD. Following NDST3 administration, the DN and its associated circuits in the substantia nigra and striatum showed marked revitalization, accompanied by a considerable alleviation of PD‐like motor deficits. Integrative single‐cell and spatial RNA sequencing with CUT&RUN revealed that NDST3‐driven reactivation of pathways is linked to neuronal survival, synaptic integrity, and steering of compromised cells toward a resilient phenotype. By recalibrating the epigenetic landscape, NDST3 promotes cellular maintenance and functional recovery in key motor circuits. These findings highlight NDST3's therapeutic potential as an epigenetic modulator of PD. These findings provide insights into the mechanisms underlying neuronal revitalization and highlight the potential of NDST3 as a novel agent for restoring brain function in neurodegenerative conditions.

## Graphical abstract

NDST3‐mediated epigenetic reprogramming revitalizes neuronal circuits in the substantia nigra and striatum to halt dopaminergic neuron degeneration and restore motor function in Parkinson's disease models. This strategy promotes neuronal maintenance and functional recovery, highlighting NDST3's therapeutic potential in neurodegenerative disorders.

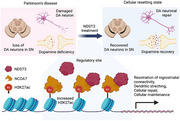

## 1 Introduction

Living mechanisms are orchestrated through the intricate interplay between cellular machinery and regulatory information encoded in the genome and epigenome, analogous to the hardware and software of biological systems. The maintenance of cellular homeostasis and DNA self‐repair is essential for the proper functioning of multicellular organisms, enabling cells to respond dynamically to internal and external stimuli while preserving their identity and function.^[^ ^1^, ^2^ ^]^ In the context of Parkinson's disease (PD), mounting evidence suggests a crucial role for epigenetic modifications in disease pathogenesis and progression.^[^ ^3^, ^4^, ^5^, ^6^, ^7^, ^8^, ^9^ ^]^ These epigenetic alterations, including histone modifications, have been implicated in the dysregulation of key PD‐associated genes and pathways, contributing to the loss of dopaminergic neurons (DN) and the accumulation of α‐synuclein aggregates.

Recent studies have shown that the relationship between aging and PD is characterized by several interconnected processes that contribute to DN vulnerability, particularly in the ventral tier of the substantia nigra pars compacta (SNpc).^[^ ^10^, ^11^, ^12^ ^]^ As age advances, proteasomal and lysosomal dysfunction become more pronounced, leading to α‐synuclein accumulation. Concurrently, markers of oxidative and nitrative stress showed a consistent upward trend, creating a heightened oxidative environment that could damage cellular components. Inflammatory processes also become more prevalent, with elevated levels of inflammatory markers and microglial activation generating a proinflammatory environment. Crucially, these age‐related changes exhibit regional specificity and are most pronounced in the ventral tier of the SNpc, which contains the DN that is most vulnerable to degeneration in patients with PD. This specificity aligns with the pattern of dopaminergic neuronal impairment and loss observed in PD, suggesting that the aging process may establish a pre‐Parkinsonian state in this region. Collectively, these age‐related alterations contribute to creating a cellular environment that nears the biological threshold for Parkinsonism, offering insight into why age is the primary risk factor for PD and suggesting that the disease may represent an extreme manifestation of normal aging in susceptible neuronal populations.

Recent advances have deepened our understanding of the intricate relationship between epigenetic integrity and cellular longevity. Previous studies showed that aging is profoundly influenced by the progressive loss of epigenetic information, a phenomenon that critically undermines the intrinsic DNA repair mechanisms in cells.^[^ ^3^, ^13^ ^]^ Their findings showed that epigenetic marks, including histone modifications such as H3K27ac and H3K4me3, are highly enriched at DNA repair sites. This enrichment leads to the accumulation of genomic stability, rendering the cells less susceptible to mutations and impaired functionality. In PD, epigenetic modulation is critical for dopaminergic neuronal survival, as these cells are vulnerable to oxidative stress and proteotoxicity, highlighting a mechanistic link between aging‐associated epigenetic alterations and PD pathogenesis.

We propose the introduction of an epigenetic regulator, specifically the NDST3 gene identified in our study, as a potential therapeutic factor for PD. NDST3 has shown significant efficacy in revitalizing and regenerating damaged cells within the substantia nigra (SN) and striatum (ST) regions in various PD models, resulting in notable improvements in motor function and survival of DN. This study extends our initial findings by elaborating on NDST3's role in maintaining cellular homeostasis and its potential as a therapeutic factor for neurodegenerative diseases such as PD. By using a combination of in vitro and in vivo settings, encompassing cell culture and diverse PD animal models, we elucidate NDST3's pivotal role in resetting damaged DN and associated cells and enhancing their survival and function. Our results illuminate the intricate mechanisms of cellular resetting through epigenetic modulation and identify a promising therapeutic target for restoring cerebral homeostasis, highlighting the potential of cellular resetting technology in devising innovative regenerative therapies for neurodegenerative diseases.

This study represents a seminal advance in the understanding of cellular homeostasis and the epigenetic landscape, establishing a foundational framework for overcoming the current constraints in personalized regenerative medicine and moving toward the design of standardized pharmaceutical interventions. By elucidating the mechanisms underlying cellular recovery and stability, our findings provide essential insights that are both fundamental and pivotal for future research and the strategic development of therapeutic modalities in this field. Moreover, these insights will streamline the trajectory for designing and refining precisely targeted interventions, contributing to a more effective and precise paradigm for devising treatment strategies tailored to address neurodegenerative diseases.

## 2 Results

### 2.1 Screening and Confirmation of an Epigenetic Modulator that Plays a Key Role in PD

**Figure 1:**
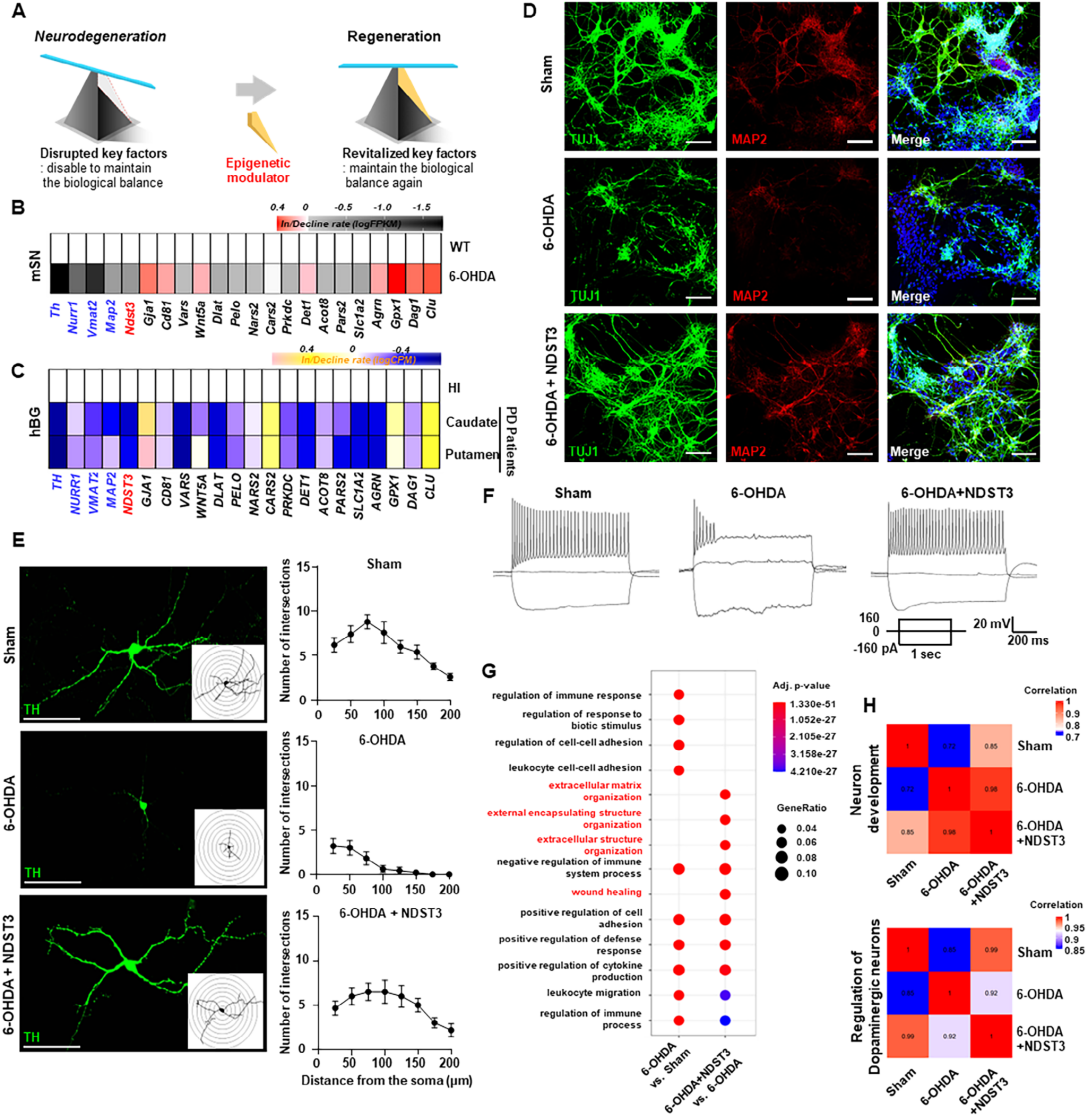
Identification of regenerating factor as a regulator of therapeutic genes for Parkinson's disease therapy. A) Conceptual diagram outlining the basis of an epigenetic regulator. B) Comparative gene expression heatmap of substantia nigra (SN) in wild type control versus 6‐OHDA‐induced Parkinson's disease (PD) mouse model. C) Heatmap showing gene expression profiles in the caudate and putamen regions of healthy individuals (HI) and a cohort of human PD patients. BG: Basal Ganglia. D) Immunofluorescence images showing TUJ1‐ and MAP2‐positive cells under each condition. Scale bar = 50 µm. E) Immunochemistry and Sholl analysis of TH‐labeled neurons. Left panel: morphology of individual neurons. Right panel: Sholl analysis showing the number of neurite intersections as a function of distance from the soma. Scale bar = 100 µm. The data are presented as mean ± SEM (*n* = 5 – 6 cells per group). F) Representative traces of action potentials evoked by depolarizing current injections under each condition (sham, 6‐OHDA, 6‐OHDA+NDST3). G) Dot plot showing the top 14 GO Biological Process terms from enrichment analyses: 6‐OHDA versus Sham (left side) and 6‐OHDA+NDST3 versus 6‐OHDA (right side). H) Pearson correlation matrix of transcriptomic among samples.

In biological systems, a delicate equilibrium underlies normal functioning, whereas degeneration and dysfunction reflect the breakdown of this homeostatic balance. Neurodegenerative diseases, such as PD, exemplify the challenge of maintaining this equilibrium in the face of progressive neuronal damage to the SN‐ST circuit. To address these challenges, we developed a screening approach to identify the fundamental regulatory factors and cellular repair molecules that preserve biological processes under pathological conditions. When these critical mechanisms are disrupted, neurotransmitter production, particularly dopamine and serotonin, is compromised, contributing to the pathology of PD. By focusing on reinstating this essential balance, our strategy aimed to offer a comprehensive therapeutic approach for managing PD (**Figure** **1**A).

Before candidate discovery, we verified the differentiation status of 3D‑optimized (OPTI) cultures using immunocytochemistry and western blotting (WB) (Figure S1A–D, Supporting Information). We quantified the neuronal markers βIII‑tubulin (TUJ1) and microtubule‐associated protein 2 (MAP2) in human neurons derived from human neural stem cells (hNSCs) under conventional 2D and 3D OPTI conditions at 0, 3, 8, and 18 days post‑induction (Figure S1B–D, Supporting Information). Both markers increased progressively over time in both conditions, with a more pronounced increase in the 3D OPTI cultures (Figure S1D, Supporting Information), indicating that the 3D environment enhanced neuronal maturation relative to the 2D environment. Using the same biological replicates after confirming marker expression, we performed LC‐MS/MS‐based proteomic profiling to comprehensively screen and identify potential therapeutic modulators (Figure S2, Supporting Information). First, we examined the expression of markers associated with DNA double‐strand breaks (DSBs).

Compared with the controls, the 3D OPTI cultures exhibited a significant reduction in key DSB markers, including *γ‐H2AX*, *MRE11*, and *BRCA2* (Figure S1E, Supporting Information).^[^ ^3^, ^14^, ^15^, ^16^, ^17^, ^18^ ^]^ These findings suggest that the 3D OPTI culture environment closely recapitulates in vivo conditions, supporting enhanced intrinsic molecular repair processes.

Under both conditions, computational analysis using neighboring clustering algorithms segregated the proteomic data into four clusters (Figures S2B,C, Supporting Information). Of these, Cluster 4 showed a strong enrichment of proteins involved in neuronal lineage specification, differentiation, synapse formation, epigenetic regulation, and neuronal fate determination, reflecting a more complex neural environment than that observed in the control group (Figures S2D,E, Supporting Information). Based on these findings, we hypothesized that the therapeutic factors of interest resided in Cluster 4.

To refine the identification of potential PD targets, we performed RNA transcriptomics in both wild type (WT) control and 6‐OHDA–induced PD animal models, focusing on 18 proteins identified in the proteomic screen as key regulators of metabolic and catabolic pathways, as well as wound healing processes, which are central components of cellular homeostasis and repair (Figure 1B; Figure S2A, Supporting Information). Among these candidates, ten protein‐coding genes were markedly downregulated in the PD model, showing a reduction in dopaminergic neuronal markers, including tyrosine hydroxylase (*Th*), nuclear receptor subfamily 4 group A member 2 (*Nurr1*), vesicular monoamine transporter 2 (*Vmat2*), and *Map2* (Figure 1B). Preliminary experiments inducing excessive oxidative stress in primary neurons using H_2_O_2_ treatment revealed that NDST3 elicited the most significant recovery of neuronal markers such as TUJ1 and MAP2 compared to the other candidate‐treated groups besides NDST3 (Figures S3A,B, Supporting Information). NDST3 subsequently emerged as a prominent therapeutic candidate because of its consistent and pronounced downregulation in both mouse and human PD samples, suggesting a possible loss‐of‐function (Figure 1B,C). To further confirm the relevance of NDST3, we examined its regional expression in mouse brains. Western blot analysis revealed that NDST3 was broadly expressed in multiple brain regions, including the midbrain, cortex, cerebellum, and hippocampus (Figure S3C, Supporting Information). This widespread expression profile suggests that NDST3 plays an essential role in various neural circuits and highlights its potential as a therapeutic target for disorders involving diverse brain regions. Specifically, the caudate and putamen regions in patients with PD exhibited significantly lower NDST3 expression than those in healthy individuals (HI) (Figure 1C). This finding was further validated using a publicly available human dataset (GSE205450) comprising samples from 75 individuals (Figure S4, Supporting Information).^[^ ^19^ ^]^ The consistent and substantial downregulation of NDST3 underscores its critical involvement in the pathophysiology of PD and highlights its potential therapeutic target.

### 2.2 Restorative Impact of the NDST3 on Impaired Neuronal Conditions

We investigated the restorative effects of NDST3 on dopaminergic neuronal circuits by verifying the successful upregulation of NDST3 in primary DN. We introduced AAV‐CMV‐NDST3 into primary dopaminergic neuronal cultures and confirmed a significant increase in NDST3 expression compared with that in the control (Figures S5A,B, Supporting Information). Following validation, NDST3 was administered to primary dopaminergic neuronal cultures (in vitro), and the findings were validated in a 6‐hydroxydopamine (6‐OHDA)‐induced Parkinsonian mouse model (in vivo), a widely used PD model. Ten days after NDST3 administration to 6‐OHDA‐treated primary DN in vitro, we observed a marked recovery of TH and SYN1 expression, which are key markers of dopaminergic integrity and mature synaptic function (Figures S5C,D, Supporting Information). To further assess neuronal viability, we quantified the cells expressing the mature neuronal markers TUJ1 and MAP2, noting a significant restoration of neuronal numbers following NDST3 treatment under the 6‐OHDA–treated condition (Figure 1D; Figures S5E,F, Supporting Information). Single‐cell pathological analysis of TH‐positive neurons revealed an increase in soma size and elongated axons, with more extensive dendritic branching in the NDST3‐treated group, indicating robust structural recovery at the cellular level (Figure 1E; Figure S5G, Supporting Information).

Building on the observed functional recovery, we assessed the electrophysiological profiles of neurons to determine whether the molecular improvements translated into functional reinstatement in the neural network. We conducted comprehensive electrophysiological analyses focusing on two key parameters of neuronal function: action potential (AP) dynamics and resting membrane potential (RMP) (Figure 1F; Figures S6A–I, and Table S1, Supporting Information). In the 6‐OHDA–treated group, 22.2% of the cells failed to fire APs, and 55.6% exhibited immature AP firing (Figure S6I, Supporting Information). In contrast, NDST3 administration dramatically improved these outcomes; no cells (0%) failed to fire APs, and only 9.1% exhibited immature AP firing, a profile closely resembling that of the healthy control group (0% non‐AP firing and 12.5% immature AP firing) (Figure 1F; Figures S6A,I and Table S1, Supporting Information). Furthermore, the RMP values in the NDST3‐treated group were similar to those in healthy controls, collectively indicating that NDST3 restores the molecular markers of neuronal health and the functional electrophysiological properties of previously impaired neurons (Figure S6H, Supporting Information).

### 2.3 The Therapeutic Effect of NDST3 in the 6‐OHDA‐Induced PD Model

Before elucidating the molecular phenotype of NDST3 in PD, we introduced an AAV9‐CMV‐eGFP group along with a sham condition to account for potential vector‐related effects. Behavioral analysis revealed that AAV9‐CMV‐eGFP administration alone induced no significant deficits, as confirmed by the lack of difference in the apomorphine‐induced rotation test compared with that in the sham group (Figure S7A, Supporting Information). Furthermore, quantitative PCR analyses of inflammatory markers (*Il‐6*, *Il‐10*, and *Tnf‐α*) showed no significant expression changes between the Sham and AAV9‐CMV‐eGFP groups (Figure S7B, Supporting Information). These data confirm that the observed behavioral phenotype in NDST3‐treated mice can be attributed specifically to NDST3, rather than to vector‐related artifacts.

To further validate the 6‐OHDA‐induced PD mouse model, we quantified TH‐positive cells in the SN at 1, 3, 5, and 7 days after 6‐OHDA administration. This analysis revealed a progressive loss of TH‐positive cells, with maximal dopaminergic neuronal loss occurring 5 days after post‐6‐OHDA treatment (Figures S7C,D, Supporting Information). These results confirmed the successful establishment of a PD animal model.

To investigate the molecular phenotype of NDST3, we used a 6‐OHDA‐induced PD mouse model, which is widely used in neurodegenerative research. We conducted bulk RNA transcriptomics (bulk RNA‐Seq) to analyze the total mRNA expression patterns across three groups: healthy controls (sham), 6‐OHDA–induced PD model, and NDST3‐treated PD model. Gene ontology (GO) analysis was performed to identify the specific biological processes influenced by NDST3 treatment (Figure 1G). This comparative approach allowed us to isolate gene expression patterns uniquely associated with NDST3 treatment in the context of 6‐OHDA‐induced neurodegeneration. Our analysis revealed significant enrichment of genes related to “extracellular structural processes,” which are crucial for neural network formation and regeneration (Figure 1G). These findings suggest that NDST3 promotes neural repair and restructuring in PD models. Our investigation revealed strong correlations between the healthy control (sham), 6‐OHDA–induced PD model, and NDST3‐treated PD model. The correlation heatmap showed that the NDST3‐treated PD model exhibited a higher correlation with the healthy control (sham) regarding dopaminergic neuron lineage and neuronal development ontology (Figure 1H). In contrast, the 6‐OHDA‐induced PD model exhibited a lower correlation with healthy controls (sham) in these categories (Figure 1H). NDST3 treatment in the 6‐OHDA‐induced PD model led to the restoration of genes related to the “DNA self‐repairing system,” including “mitochondrial DNA repair,” to levels comparable to those in the healthy control (sham) (Figure S8A, Supporting Information). These findings suggest that NDST3 treatment plays a crucial role in promoting neuronal integrity and maintenance in the context of 6‐OHDA–induced neurodegeneration, demonstrating a molecular correlation pattern similar to that in healthy controls (sham) in the regulation of DN and general neuronal development.

**Figure 2:**
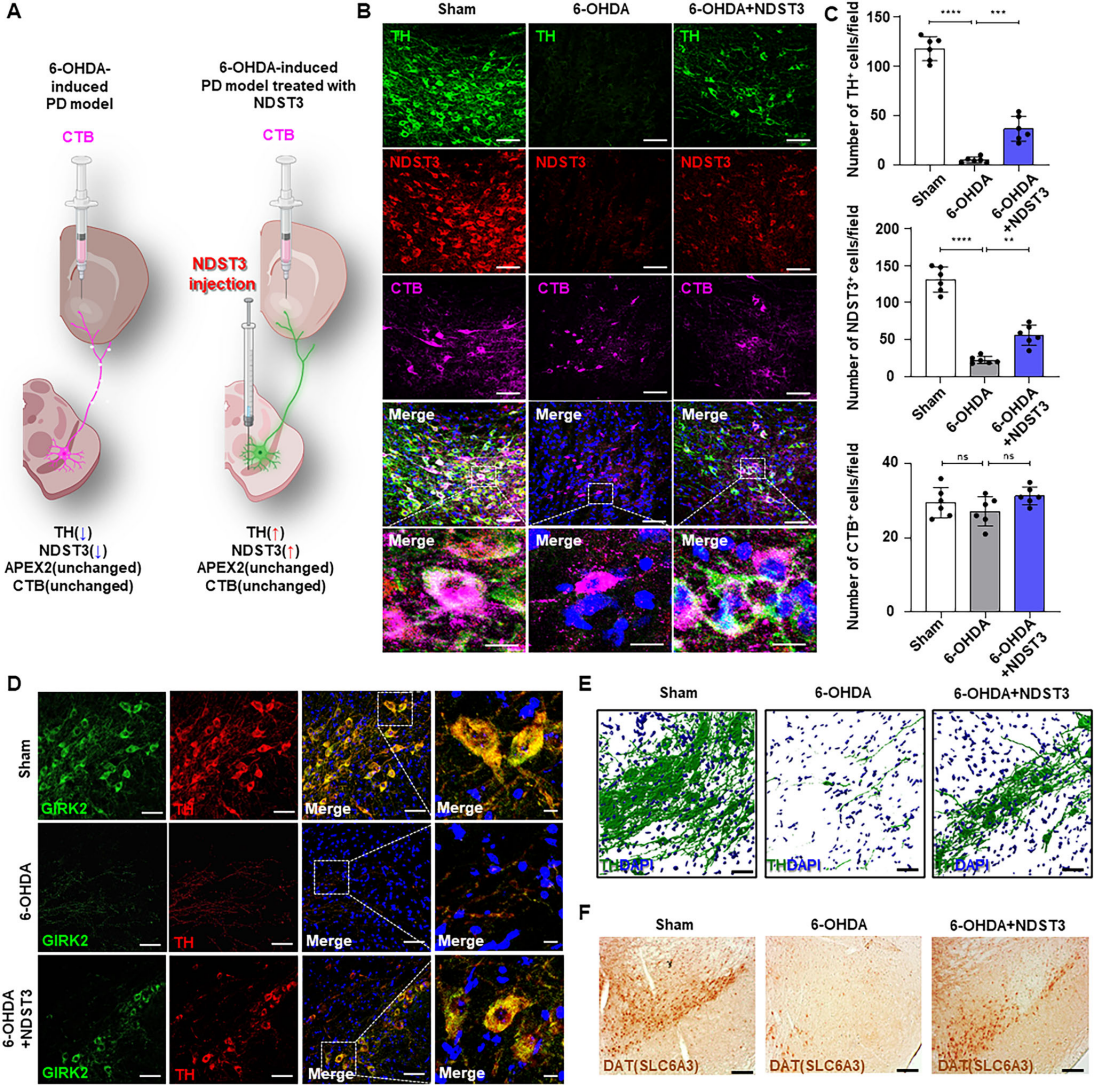
Therapeutic efficacy of NDST3 and retrograde tracing with CTB in mice. A) Schematic diagram of in vivo experimental design involving CTB injection in the PD mouse model. B) Representative immunofluorescence images of CTB, TH, and NDST3 expression in the SN of Sham, 6‐OHDA‐induced PD mice, and NDST3‐treated PD mice. Scale bar = 50 µm and 10 µm (Magnified image). C) Quantification of CTB‐, TH‐, and NDST3‐positive cells shown in Figure 2B. Data are presented as mean ± SEM (*n* = 6 independent animals per group). One‐way ANOVA with Tukey's multiple comparisons test. **p < 0.01, ***p < 0.001, ****p < 0.0001, and ns = not significant.  D) Immunofluorescence images showing GIRK2‐ and TH‐positive cells in the Sham, 6‐OHDA‐induced PD mice, and NDST3‐treated PD mice. Scale bar = 50 µm and 10 µm (Magnified image). E) 3D Z‐stack analysis (IMARIS) of TH‐positive neurons obtained via confocal microscopy. F) DAB‐DAT staining in the SN.

In addition, we investigated the projection pattern of TH‐positive DN originating in the SN and extending to the ST to elucidate the role of NDST3 in dopaminergic neuron regeneration. To determine whether the effects of NDST3 were because to neuronal differentiation from neural precursors or cell regeneration, we introduced a retrograde tracer Cholera Toxin Subunit B (CTB) (**Figure** **2**A). We found that cells labeled with CTB on the ipsilateral side treated with NDST3 exhibited upregulation of Th expression compared to the 6‐OHDA‐induced PD model, whereas the expression of *Apex*1 and 2, subcellular markers indicative of DN, remained unaltered but lost their functionality (Figure S6J, Supporting Information).^[^ ^20^, ^21^ ^]^ Subsequent analysis of the SN revealed stark contrasts between the different treatment groups. In the healthy control (sham), as expected, we observed that TH‐positive cells co‐localized with CTB, indicating intact dopaminergic projections. However, in the 6‐OHDA‐induced PD model, TH‐positive cells were absent, and only CTB‐positive cells remained, suggesting that 6‐OHDA impairs DN and renders it TH negative.

In contrast, in the NDST3‐treated cohort of the 6‐OHDA‐induced PD model, a significant resurgence of cells co‐expressing TH and CTB was observed. This marked increase in TH and CTB double‐positive cells in the NDST3‐treated PD model compared to that in the 6‐OHDA‐induced PD model demonstrated the regenerative capacity of NDST3 (Figures 2B,C). Additionally, to evaluate the regional change of dopaminergic marker following NDST3 treatment in the PD, sagittal immunohistochemical analyses were performed. Examination of the SN, ST, and the nigrostriatal fiber (NF) tract was conducted using CTB tracing, which enables objective visualization of projection in these regions (Figures S9A,B, Supporting Information). Across all groups, quantification of CTB‐positive cells revealed no significant difference in these regions. However, expression of TH and NDST3 was reduced in the SN, ST, and NF of 6‐OHDA group compared to the sham. Remarkably, NDST3 treatment led to an increase in TH and NDST3 expression across these regions compared to the 6‐OHDA group, indicating restoration of dopaminergic phenotype in the nigrostriatal axis.

Importantly, these results indicate that NDST3 facilitates cellular restoration of dopaminergic neurons in a damaged neural environment. The ability of NDST3 to restore dopaminergic neuron functionality and phenotype in a damaged neural environment across the neural circuit provides compelling evidence of its role as a potent neuron‐regenerating molecule that acts through cellular resetting mechanisms.

The ventral tegmental area (VTA) is integral to the brain's reward circuitry, with the DN projecting to various brain regions, including the nucleus accumbens within the mesolimbic pathway.^[^ ^22^, ^23^ ^]^ The SNpc is predominantly recognized for dopamine (DA) production, with the DN forming a nigrostriatal pathway that extends to the striatum.^[^ ^24^, ^25^ ^]^ In contrast, the substantia nigra pars lateralis (SNL) is associated with inhibitory neuronal regulation, modulating the SNpc and other midbrain regions.^[^ ^26^, ^27^ ^]^ We examined three regions (VTA, SNpc, and SNL) in both 6‐OHDA‐induced and NDST3‐treated PD models. Interestingly, whereas a sharp reduction in TH‐positive neurons was observed in the 6‐OHDA‐induced PD model, the NDST3‐treated PD model demonstrated a dramatic rescue of DN across all three regions (Figures S9C,D, Supporting Information). No significant difference in TH expression was observed between the Sham and NDST3‐only treated groups (Figures S9C,D, Supporting Information).

We also examined macromorphological properties by analyzing TH‐positive cells after NDST3 treatment in a PD mouse model. We observed an increase in fluorescence intensity following NDST3 administration (Figure 2E). Further molecular analysis revealed the restoration of key dopaminergic neuronal lineage markers, including G protein‐gated inwardly rectifying potassium channel 2 (GIRK2) and TH. These markers were restored in the NDST3‐treated PD model, indicating molecular recovery along with the observed neuronal regeneration (Figure 2D; Figure S9E, Supporting Information).

Besides molecular and cellular analyses, we assessed the potential of NDST3 administration to ameliorate Parkinsonian phenotypes in a 6‐OHDA–induced PD model. Employing the tail suspension test and an apomorphine‐induced rotatory test tailored for 6‐OHDA–induced motor impairment, we observed a pronounced improvement in Parkinsonian phenotypes after NDST3 treatment (Figures S9F,G, Supporting Information). There were no detectable differences in the behavioral outcomes between the sham and NDST3‐only treated groups (Figures S9F,G, Supporting Information). This behavioral recovery suggests functional restoration of motor control, which is indicative of the therapeutic efficacy of NDST3. To further support these findings at the molecular level, we examined dopamine transporter (DAT) expression in the NDST3‐treated PD models. DAB staining revealed a substantial increase in DAT‐positive cells, suggesting that NDST3 administration restores DN and stabilizes the dopamine neuronal circuitry, potentially revitalizing DA receptor function and enhancing the efficacy of the overall neurotransmitter system (Figure 2F; Figure S9H, Supporting Information). This includes the reinstatement of dopamine release and normalization of dopamine transport mechanisms, which are key processes typically disrupted in PD.^[^ ^28^, ^29^ ^]^

**Figure 3:**
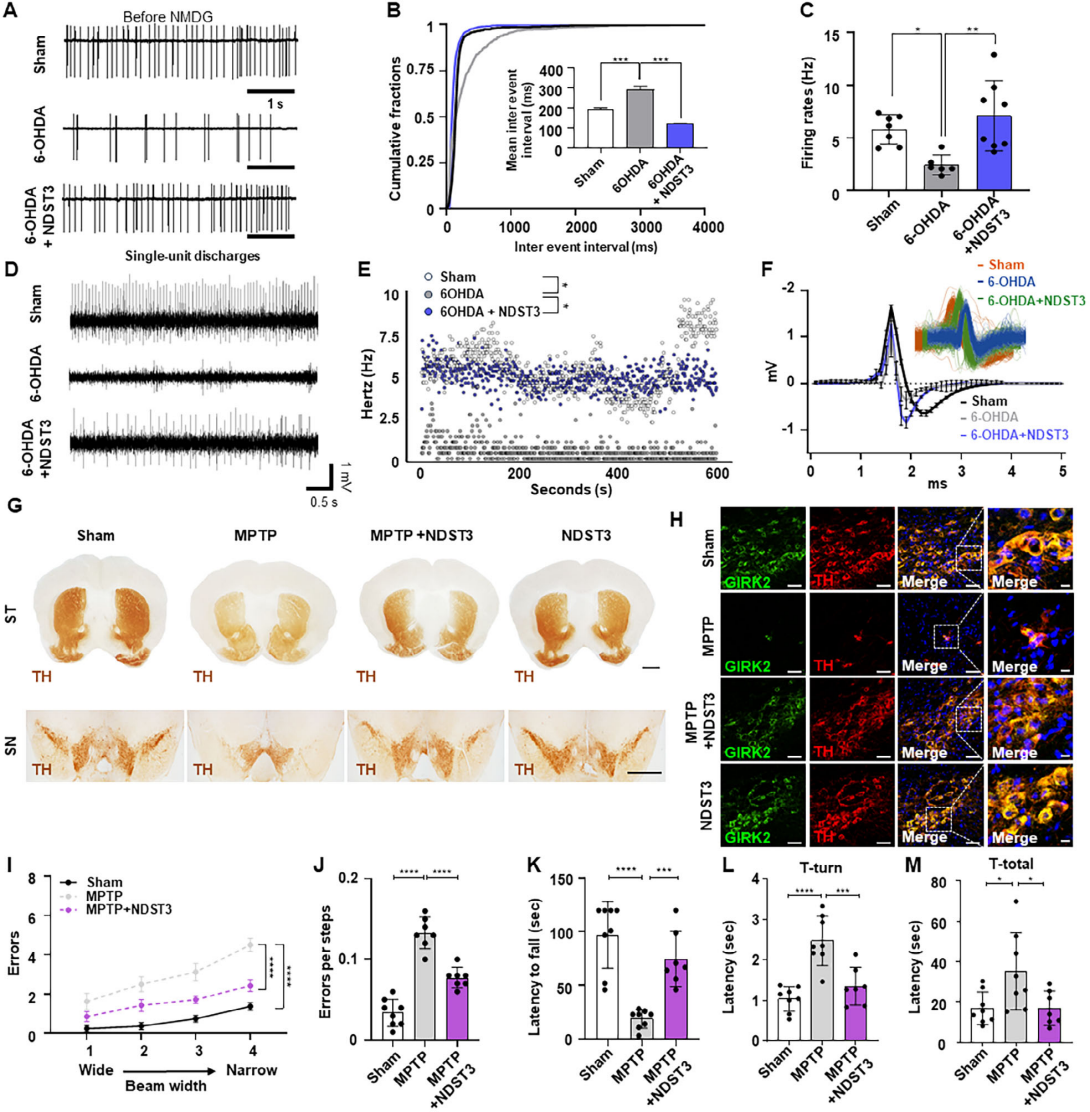
Efficacy and electrophysiological properties of NDST3 in chemical‐induced PD model. A) Representative traces of spontaneous firing currents recorded from DA neurons of the SNpc in brain slices from each group. B) Cumulative fractions curves showing shortened inter‐event intervals, indicating a higher frequency of spontaneous firing in the 6‐OHDA + NDST3 group compared to the 6‐OHDA group. The inner bar graph showed mean inter‐event intervals in the ipsilateral of SNpc of each group. Data are presented as mean ± SEM (*n* = 6 – 8 independent animals per group). One‐way ANOVA with Tukey's multiple comparisons test. ***p < 0.001. C) Quantification of DA neuronal firing rates in the ipsilateral SNpc of each group. The data are presented as mean ± SEM (*n* = 6–8 independent animals per group). One‐way ANOVA with Tukey's multiple comparisons test. *p < 0.05, and **p < 0.01. D) Representative in vivo recording traces from the SNpc of live animals in each condition. E) Instantaneous firing frequencies during the recorded period. (*n* = 4–6 independent animals per group; repeated measures) Two‐way ANOVA with Tukey's multiple comparisons test, *p < 0.05. F) Comparison of action potential waveforms among DA neurons across conditions. G) Representative image of DAB‐TH staining in ST and SN. Scale bar = 1 mm. H) Immunofluorescence images showing GIRK2‐ and TH‐positive cells in the Sham, MPTP‐induced PD mice, NDST3‐treated PD mice, and NDST3 only‐treated mice. Scale bar = 50 µm and 10 µm (Magnified image). I) Error count during the challenging beam traversal test for each experimental condition. The data are presented as mean ± SEM. (*n* = 7 – 8 independent animals per group) Two‐way ANOVA with Tukey's multiple comparisons test. ****p < 0.0001. J) Errors per step during the challenging beam traversal test across conditions. The data are presented as mean ± SEM (*n* = 7 – 8 independent animal per group). One‐way ANOVA with Tukey's multiple comparisons test. ****p < 0.0001. K) Fall latency in the wire‐hanging test. The data are presented as mean ± SEM (*n* = 7–8 independent animals per group). One‐way ANOVA with Tukey's multiple comparisons test. ***p < 0.001 and ****p < 0.0001. L) Time to orient downward (T‐turn) and M) time to descend to the base (T‐total). The data are presented as mean ± SEM (*n* = 7–8 independent animals per group). One‐way ANOVA with Tukey's multiple comparisons test. *p < 0.05, ***p < 0.001 and ****p < 0.0001.

The observed improvements in Parkinsonian phenotypes and molecular restoration of dopamine transport mechanisms prompted a more in‐depth exploration of NDST3's impact on neuronal electrophysiology. To investigate how these macroscopic and molecular changes translate into functional neuronal activity, we conducted *ex vivo* slice and in vivo extracellular recordings (**Figures** **3**A–F; Figures S10A–D, Supporting Information). This electrophysiological approach enabled the direct assessment of neuronal firing patterns and synaptic function, offering crucial insights into NDST3 functional efficacy in PD models.^[^ ^30^, ^31^ ^]^ In the 6‐OHDA‐induced PD model, single‐unit discharge analysis of the DN in the SN revealed significant functional recovery following NDST3 treatment. This was indicated by a marked improvement in the coordination of neuronal firing rates and inter‐event intervals in slice patch recordings (Figures 3A–C). In addition, treatment with NMDG, which reduces neuronal excitability by replacing extracellular Na^+^, abolished action potentials in all three groups (Figure S10A, Supporting Information). Furthermore, the mean coordination ratio in vivo was higher in the NDST3‐treated PD model and closely resembled that of healthy controls (sham) (Figures 3D–F; Figures S10B–D, Supporting Information). Waveform analysis of in vivo extracellular recordings further confirmed this therapeutic effect. In the NDST3‐treated PD model, the waveforms exhibited rescued morphology, indicating improved neuronal activity (Figure 3F; Figure S10C, Supporting Information). These results provide additional functional evidence of NDST3 efficacy, showing significant recovery in the NDST3‐treated PD model compared to the PD model.

Based on these findings, we hypothesized that NDST3 treatment would restore dopamine release in a PD model. To test this hypothesis, we performed LC‐MS analysis to quantify the dopamine levels in the striatum. Remarkably, the NDST3‐treated PD model exhibited dopamine release of 12.67 ± 2.88 pg µL^−1^, closely resembling the healthy control (sham), which showed a dopamine release of 12.55 ± 2.58 pg µL^−1^ (Figure S10E, Supporting Information). In contrast, the 6‐OHDA‐induced PD model released only 8.82 ± 1.94 pg µL^−1^ of dopamine, underscoring the effectiveness of NDST3 in restoring dopamine to near‐normal levels in the brain.

### 2.4 Therapeutic effect of NDST3 in the MPTP‐induced PD model

To investigate the effect of NDST3 in an N‐methyl‐4‐phenyl‐1,2,3,6‐tetrahydropyridine (MPTP)‐induced PD mouse model, we first validated the process by quantifying TH‐positive cells in the SN at 1–4 weeks after MPTP treatment. We found a significant decrease in the latency to fall and a progressive decline in TH‐positive cells, with maximal dopaminergic neuronal loss occurring 4 weeks post‐MPTP treatment. In agreement with previous studies, TH‐positive cell loss began ≈2 weeks after MPTP treatment and continued to deteriorate (Figures S11A,B, Supporting Information).

To further evaluate NDST3's therapeutic potential, we examined its effects on the MPTP‐induced mouse model of PD. Two weeks after post‐NDST3 administration in the SN region, we assessed the molecular mechanisms underlying these effects. We analyzed the mRNA and protein levels of key dopaminergic neuronal lineage markers in an MPTP‐induced PD model. We measured the expression levels of *Th*, *Dat*, and *Nurr1*. Our results revealed a marked reversal in the protein and mRNA expression levels of these markers in the NDST3‐treated PD model, indicating the upregulation of dopaminergic markers (Figures S11C ^−^E, Supporting Information). Histological analysis of coronal brain sections from NDST3‐treated PD models further supported this finding, showing an increased number of TH‐DAB‐positive cells in the SN and enhanced signal intensity in the ST (Figure 3G; Figure S11F, Supporting Information). Moreover, a higher number of DAT‐DAB‐positive neurons was observed in the NDST3‐treated PD model than in the MPTP‐induced PD model, suggesting improved dopaminergic neuron functionality (Figures S11G,H, Supporting Information). Consistently, the NDST3‐treated PD group exhibited higher expression of the DA neuron markers GIRK2 and TH (Figure 3H; Figure S11I, Supporting Information).

We assessed the severity of Parkinsonian symptoms using behavioral tests, including the hanging, pole, and challenging beam traversal tests. Remarkably, NDST3‐treated PD models showed notable improvement in the challenging beam traversal test, with performance closely resembling that of the healthy controls. This was underscored by a significant reduction in the traversal errors (Figure 3I). The number of errors per step was significantly lower in the NDST3‐treated PD model than in the MPTP‐induced model (Figure 3J). The hanging ability assessment revealed a significant increase in the latency to fall in the NDST3‐treated PD model, in contrast to the reduced latency observed in the MPTP‐only induced PD model (Figure 3K). In the pole test, the NDST3‐treated PD model exhibited notably improved motor function, as evidenced by significantly reduced latency times compared to the MPTP‐induced PD model (Figures 3L,M). These findings suggest that NDST3 administration promotes neuronal circuitry regeneration, thereby ameliorating motor deficits in mice.

### 2.5 Molecular phenotypes of PD under NDST3 administration

Building on these observations, we aimed to elucidate the molecular mechanisms of NDST3 in a 6‐OHDA‐induced PD mouse model, which is widely used because of its broad relevance in neurodegenerative research.^[^ ^32^, ^33^, ^34^, ^35^, ^36^ ^]^ Unlike the MPTP model, which primarily targets DN, the 6‐OHDA model induces broader neuronal disruption, affecting monoaminergic neurons involved in noradrenaline and serotonin signaling.^[^ ^34^, ^37^, ^38^, ^39^ ^]^ This leads to widespread disruption of neuronal circuits and synaptic plasticity, increased reactive oxygen species (ROS) production, and triggers astrocyte reactivation, which are key factors in the pathophysiology of neurological diseases.^[^ ^40^, ^41^, ^42^, ^43^ ^]^ To dissect NDST3‐induced molecular alterations, we performed bulk RNA sequencing (RNA‐seq), single‐cell RNA sequencing (scRNA‐seq), and spatial RNA transcriptomics. This approach enabled us to comprehensively map the transcriptomic landscape across various neuronal populations affected by 6‐OHDA, providing a holistic perspective on NDST3's therapeutic impact. We specifically focused on how NDST3 modulates the expression of genes related to neuronal damage, regeneration, ROS regulation, cellular self‐repair, and astrocyte reactivation, highlighting the potential cell‐resetting mechanisms in PD.

**Figure 4:**
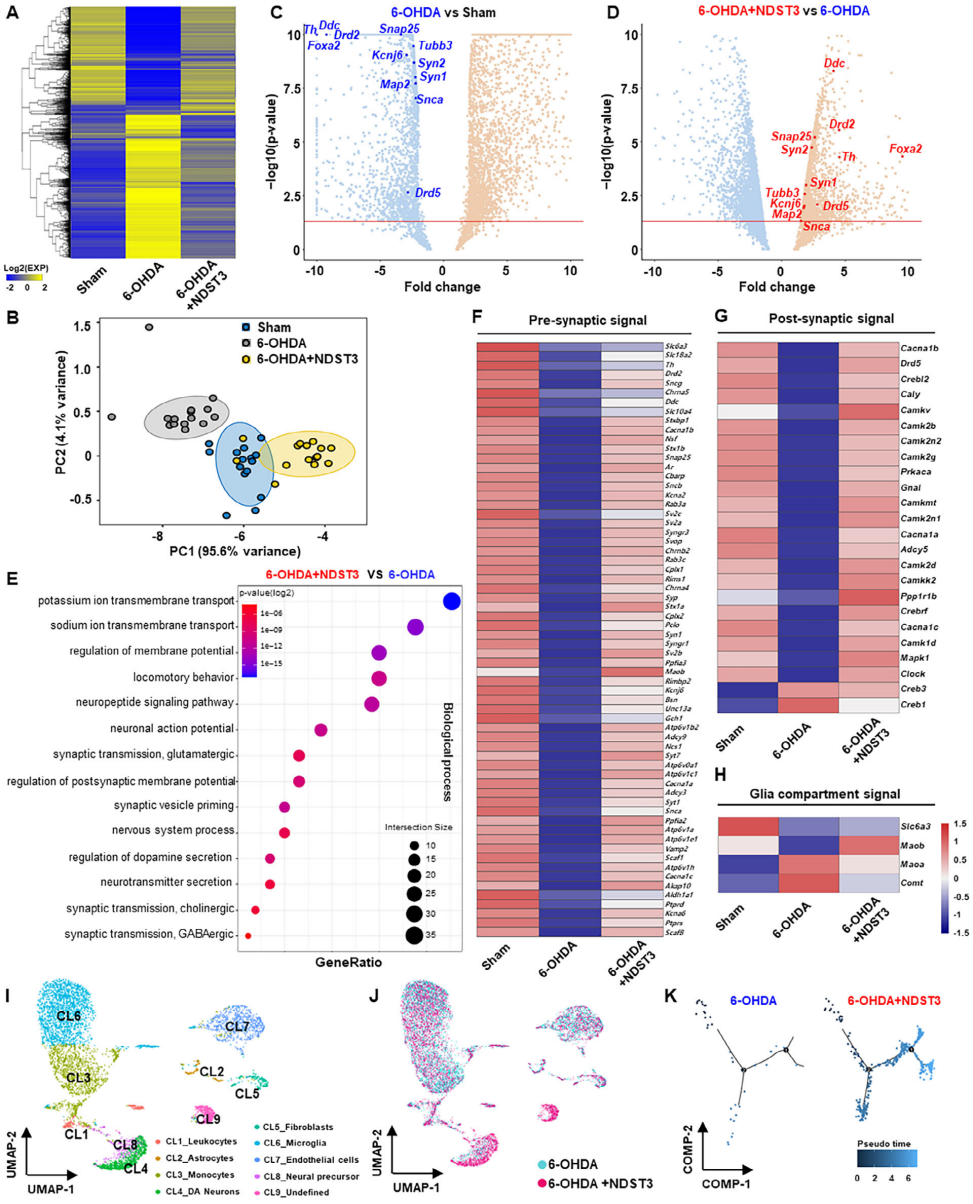
Molecular mechanisms of NDST3 in the PD model. A) One‐way hierarchical clustering heatmap based on Z‐score of normalized expression value for 5629 genes selected with fold change ≥ 2 and raw *p*‐value < 0.05. B) Principal component analysis (PCA) analysis of RNA‐seq data to visualize sample‐to‐sample variation. C) Volcano plot showing differentially expressed genes between 6‐OHDA and Sham group; Down‐regulated genes marked in blue. D) Volcano plot showing differentially expressed genes between 6‐OHDA+NDST3 and 6‐OHDA; Up‐regulated genes marked in red. E) Dot plot of top 14 GO cellular component terms from GO enrichment analyses: 6‐OHDA+NDST3 versus 6‐OHDA. Heatmap showing gene expression patterns in F) pre‐synaptic neurons, G) post‐synaptic neurons, and H) glia compartments. I) UMAP visualizing cluster identity. J) UMAP representation comparing cellular composition in 6‐OHDA and 6‐OHDA+NDST3. K) Branched trajectory analysis illustrating cell state transitions in a 2D state‐space, where each dot represents a single cell, color‐coded by group identity.

RNA‐Seq was used to examine the total mRNA expression patterns across three groups: healthy controls (sham), 6‐OHDA–induced PD model, and NDST3–treated PD model. As shown in **Figure** **4**A, the absolute gene expression profiles of a substantial cohort of genes (*n* = 5629) with > 2‐fold change showed strong similarity between the NDST3–treated PD model and healthy controls (sham) (Figure 4A). Principal component analysis (PCA) was conducted to better understand the relationships among these groups. The PCA plot showed an overlap between the sham and NDST‐treated PD groups, whereas the 6‐OHDA‐induced PD group was distinctly separated from both. These findings indicate that NDST3 administration effectively shifted the transcriptional landscape of the PD model closer to that of healthy controls, suggesting the restoration of a healthier molecular phenotype (Figure 4B).

Next, we evaluated midbrain dopaminergic neuron‐related genes in the NDST3‐treated PD model and observed a dramatic recovery of key markers, such as *Th*, dopa decarboxylase (*Ddc*), *Drd2*, forkhead box protein A2 (*Foxa2*), synapsin I (*Syn1*), synapsin I (*Syn2*), *Map2*, *Tubb3*, synaptosome associated protein 25 (*Snap25*), G protein‐activated inward rectifier potassium channel 2 (*Kcnj6;also called Grik2*), and dopamine receptor D5 (*Drd5*), which are vital for dopaminergic neuron function (Figures 4C,D). These results highlight NDST3's role in restoring cellular functions impaired by neurodegenerative conditions. To identify the specific biological processes modulated by NDST3, we performed GO analysis of a 6‐OHDA–induced PD model with and without NDST3 treatment. This analysis revealed a significant enrichment of genes involved in extracellular structural processes that are essential for the formation and regeneration of neuronal networks (Figure 4E; Figure S12, Supporting Information). Genes central to neural signaling pathways, a key criterion of our screening strategy, were markedly upregulated in the NDST3‐treated PD model compared to those in the 6‐OHDA‐induced PD model (Figure 4E).

Given the multifactorial etiology of PD, including genetic predispositions, heightened oxidative stress, and impaired neuronal–glial communication, we expanded our scope beyond dopaminergic regeneration. We hypothesized that NDST3, identified as a potential “resetting” factor, may serve as a master regulator of cellular homeostasis, affecting both neurons and glial populations. To evaluate this, we examined the expression of genes associated with presynaptic and postsynaptic DN, as well as neuron–glial signaling molecules (Figures 4F–H).

In the 6‐OHDA–induced PD model, these markers were markedly downregulated, indicating impaired dopaminergic circuits and disrupted intercellular communication. NDST3 treatment reversed these deficits in all cell types, restoring neuronal and glial gene expression (Figures 4F–H; Figure S13, Supporting Information). This robust molecular recovery suggests that NDST3 may support synaptic repair and re‐establish functional signaling between neurons and the glia.

To further explore the pathophysiology of PD, we broadened the molecular analysis beyond DN to include glutamatergic, GABAergic, and cholinergic neuronal subtypes (Figures S14–S16, Supporting Information). By examining both presynaptic and postsynaptic markers and their interactions with glial cells, we observed that NDST3 treatment substantially alleviated 6‐OHDA‐induced molecular impairments across all subtypes. This broad restorative effect supports the role of NDST3 as a potential “cell‐resetting” agent, with actions that extend beyond the dopaminergic system to diverse neuronal and glial pathways. Our findings suggest that PD involves a wider range of neuronal disruptions than previously recognized and that NDST3 offers therapeutic benefits in multiple affected cell types. To complement these molecular findings, we performed immunostaining to investigate the localization of NDST3 in glutamatergic, dopaminergic, GABAergic, and cholinergic neurons. Neuronal populations were identified using the co‐staining markers VGLUT1 for glutamatergic neurons, TH for DN, GABA R1 for GABAergic neurons, and vesicular acetylcholine transporter (VAChT) for cholinergic neurons. The NDST3‐treated groups exhibited a significant increase in the expression of these subtype‐specific markers compared to the 6‐OHDA‐induced PD group (Figure S17, Supporting Information).

Following the characterization of the broader transcriptomic changes induced by NDST3 in the 6‐OHDA‐induced PD model using RNA‐seq, we used scRNA‐seq to prove NDST3's effect at the cellular level. This high‐resolution approach enabled the dissection of NDST3‐driven transcriptional changes across a range of cell types, including DN, astrocytes, and glial cells, within the neuronal environment. Using cell marker gene data from the CellMarker database and cluster‐specific markers, we identified nine discrete cell clusters in both the PD and NDST3‐treated PD models (Figures 4I,J; Figures S18–S20, Supporting Information).^[^ ^44^ ^]^ Through dimensionality reduction, we visualized single‐cell gene expression in an unsupervised clustering format, revealing substantial increases in DN (Cluster 4), neural precursors (Cluster 8), and undefined cells (Cluster 9) in the NDST3‐treated PD model (Figures S20 and S21, Supporting Information). To validate the enrichment and functional identity of these clusters, dopaminergic development and regenerative pathways in Clusters 4 and 8 were examined. We examined the expression of critical transcription factors, including nuclear receptor 4A2 (*Nr4A2*), Zinc‐finger E‐box‐binding homeobox 1 (*Zeb1*), and calcium/calmodulin‐dependent protein kinase IV (*CamK4*), and observed significant increases in both cell numbers and expression levels following NDST3 treatment (Figure S20, Supporting Information). In addition, quantification of single‐cell counts demonstrated a marked increase in these clusters in the NDST3‐ treated PD group (Figures S21A–D, Supporting Information). Ontological analysis confirmed that pathways associated with “DNA repair,” “telomere maintenance,” and “metabolic and catabolic processes” in the NDST3‐ treated PD model, reflecting a broad enhancement of both genetic repair mechanisms and metabolic homeostasis essential for neuronal restoration (Figure S21E, Supporting Information). To further investigate the relevance of these biological processes (DNA repair, chromatin remodeling, and nervous system development), we analyzed the molecular interactions within Clusters 4, 8, and 9, respectively. The node correlative heatmap and network analysis showed that the node strengths (all > 0.8) between Cluster 4‐8, 8‐9, and 4‐9 were substantially greater than the averages (0.54) for the other clusters, suggesting coordinated resetting processes (Figure S22, Supporting Information). Moreover, to validate the activation of the DNA repair pathway in Clusters 4, 8, and 9, we conducted RT‐qPCR analysis for the key markers of the DNA repair process: *Ku70*, *Rad51*, and *Cdc25a*. These markers were significantly upregulated in the NDST3‐treated group compared to those in the sham and 6‐OHDA‐induced PD groups (Figure S22C, Supporting Information). Further comparison between the PD and NDST3‐treated PD models revealed additional ontological differences within Clusters 4, 8, and 9, which exhibited enhanced resilience via the upregulation of self‐repair and defense pathways (Figure S23, Supporting Information). Cluster 9 emerged as a distinct “resetting” population, enriched in genes associated with “spontaneous cellular maintenance,” “DNA repair,” and “homeostasis process” (Figures S23 and S24, Supporting Information), highlighting NDST3's pivotal role in promoting cellular repair and restoration. In addition, trajectory analysis using Monocle 2 revealed that NDST3 treatment promoted branching cell fate pathways in the NDST3‐treated PD model, in contrast to the linear trajectories observed in the untreated PD controls (Figure 4K).^[^ ^45^ ^]^ To further elucidate the mechanisms underlying the effect of NDST3 on PD, we integrated the findings and data from bulk RNA‐seq and scRNA‐seq (Figure S25, Supporting Information), enabling a thorough evaluation of the gene expression profiles associated with both presynaptic and postsynaptic DN functions. In Clusters 4, 8, and 9, encompassing DN development, regeneration, and “cellular resetting,” scRNA‐seq showed a notable upregulation of genes linked to both presynaptic and postsynaptic functions in the NDST3‐treated PD model (Figure S26A, Supporting Information).

**Figure 5:**
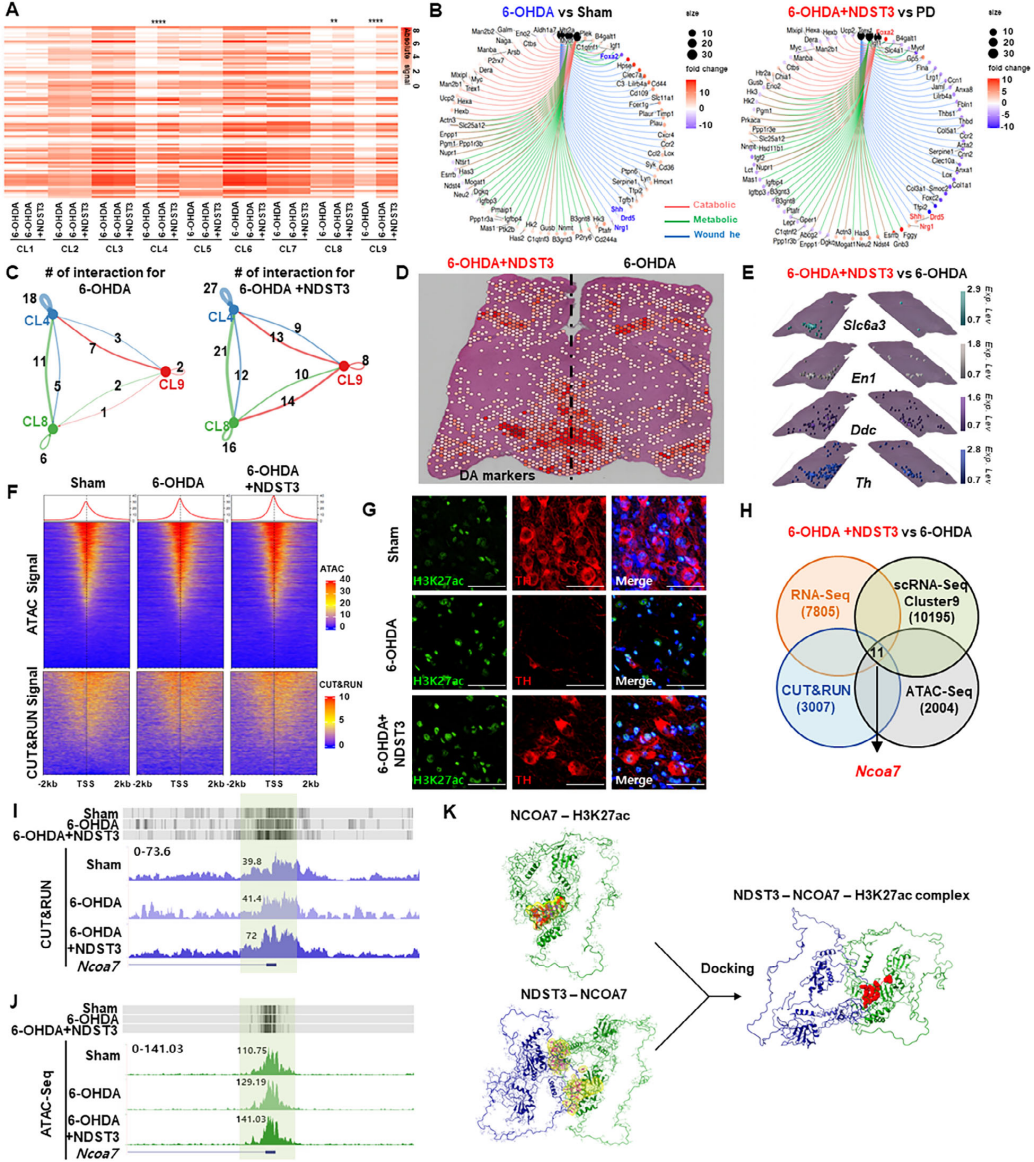
Comprehensive analysis of spatial transcriptomics and epigenetic modulation following NDST3 treatment in a PD model. A) Heatmap showing gene expression patterns in each cluster. **p < 0.01, and ****p < 0.0001. B) Gene concept network plot displaying genes enriched in catabolic, metabolic, and wound healing GO categories. The top 30 most differentially expressed genes comparing 6‐OHDA versus Sham and 6‐OHDA+NDST3 versus 6‐OHDA. Node color intensity represents the log2 fold‐change of gene expression. C) Cell‐cell communication network plot illustrating interactions among three distinct cell clusters in 6‐OHDA‐induced PD model (left panel) and NDST3‐treated PD model (right panel), based on ligand–receptor pair probabilities using the CellChat database. Line thickness indicates proportionality to the number of interactions. D) Spatial localization of dopamine‐related markers. E) Spatial mapping of dopaminergic lineage markers identified via scRNA‐Seq. F) Heatmap visualization of CUT&RUN and ATAC‐Seq signal intensity ±2 kb around the TSS. G) Immunofluorescence images showing H3K27ac and TH‐positive cells in the Sham, 6‐OHDA‐induced PD mice, and NDST3‐treated PD mice. Scale bar = 50 µm. H) Venn diagram illustrating overlapping genes among DEGs from RNA‐Seq, scRNA‐Seq Cluster 9, CUT&RUN peak, and ATAC‐Seq peak. Average signal plot of I) CUT&RUN and J) ATAC‐seq signals at over‐enriched TSS regions of the Ncoa7 gene. K) Structure of NDST3‐NCOA7‐H3K27ac complex. Blue – NDST3, Green – NCOA7, and Red – H3K27ac. The yellow boundary represents the interaction region.

This alignment between RNA‐seq and single‐cell transcriptomics validates earlier results and offers deeper insights into NDST3's effect on specific neuronal subtypes. The robust elevation of dopaminergic markers in these key clusters underscores NDST3's comprehensive restorative effect, accelerating synaptic integrity and neuronal function (**Figure** **5**A).

We further used CellChat to analyze intercellular signaling among Clusters 4, 8, and 9, revealing significantly enhanced communication in the NDST3‐treated PD model compared to that in the untreated PD model (Figure 5C). Further pathway analysis indicated that NDST3 predominantly promoted signaling cascades that were critically involved in spontaneous cellular maintenance, self‐repair, and anti‐inflammatory responses (Figure S26B, Supporting Information). This enhanced intercellular signaling underscores NDST3's therapeutic mechanism involving DN restoration. Building on these findings, we examined the interplay between catabolic, metabolic, and wound‐healing processes, which led us to identify NDST3 and midbrain dopaminergic regulators, such as *Foxa2*, sonic hedgehog (*Shh*), *Drd5*, and neuregulin 1 (*Nrg1*). These genes were notably downregulated in the PD model but exhibited significant recovery following NDST3 treatment (Figure 5B). Collectively, these findings highlight NDST3's ability to restore dopaminergic pathways and promote neuronal resilience in PD.

To deepen our analysis of regional gene expression changes in PD, we performed spatial RNA transcriptomics of the midbrain of an NDST3‐treated PD model. We first focused on DN‐associated genes (*Th*, *Ddc*, *En1*, *Slc6a3*, *Nr4a2*, *Drd2*, *Pitx2*, and *Slc18a2*) that were downregulated in the midbrain. These genes were notably upregulated in the ipsilateral midbrain region of the NDST3‐treated PD model compared to that in the vehicle‐treated controls, indicating robust restoration of dopaminergic function (Figure 5D,E, Figure S27A,B, Supporting Information). In contrast, proinflammatory markers (*Aif1*, *Cd68*, and *ITGAM*), which were elevated in the PD model, were markedly downregulated in the same region following NDST3 treatment (Figures S27C,D, Supporting Information). In parallel, scRNA‐Seq was used to analyze gene expression profiles. The scRNA‐Seq results revealed consistent patterns: DN‐associated gene expression was elevated, whereas inflammation‐related gene expression was decreased in the NDST3‐treated PD model (Figure S28, Supporting Information). These observations suggest that NDST3 restores dopaminergic function, re‐establishes cellular equilibrium, and attenuates inflammatory responses in the affected midbrain regions.

### 2.6 Mechanistic Studies of the Epigenetic Modulator NDST3 in PD Therapy

To explore the mechanisms underlying NDST3‐mediated neuronal recovery in PD, we first examined whether the upregulated gene clusters identified in our transcriptomic data correlated with data associated with specific epigenetic modifications. Preliminary analyses indicated that many of these gene clusters were linked to H3K27ac, an epigenetic marker correlated with active chromatin. Therefore, we performed cleavage under targets and release using nucleases (CUT&RUN) with an H3K27ac‐specific antibody and compared the results with ATAC‐seq profiles from DN in the NDST3‐treated PD model and 6‐OHDA‐induced PD models. CUT & RUN revealed a substantial increase in H3K27ac levels in the NDST3‐treated PD model. Consistently, ATAC‐seq showed a concomitant increase in open chromatin regions, suggesting that NDST3 induces a more open, transcriptionally active chromatin state to facilitate a spontaneous recovery process, which we term as “cell resetting‐like state” (Figure 5F; Figure S29, Supporting Information). Immunostaining further corroborated these findings, revealing H3K27ac levels in restored DN and linking these epigenetic changes to the observed improvements in neuronal morphology and function (Figure 5G).

To identify the specific genes regulated by enhanced chromatin accessibility, we integrated CUT&RUN, ATAC‐seq, RNA‐Seq, and scRNA‐seq data. This integrative analysis revealed 11 genes that were significantly upregulated following NDST3 treatment, notably *Ncoa7*, which ranked highest (Figure 5H). Consistent with these findings, we observed increased H3K27 acetylation at the transcription start site (TSS) of *Ncoa7* gene in the NDST3‐treated PD group (Figures 5I,J). Additionally, we investigated the accumulation of H3K27ac at *Th* and *Ncoa7* loci using chromatin immunoprecipitation‐qPCR (ChIP‐qPCR). Consistent with the previous results, we confirmed that the TSS of the *Ncoa7* and *Th* genes are highly enriched in H3K27ac in the NDST3‐treated group (Figure S30A, Supporting Information).

To further elucidate the molecular relationships between NDST3, NCOA7, and H3K27ac, we conducted in vitro co‐immunoprecipitation (Co‐IP) experiments. NDST3 showed no direct binding to H3K27ac; however, it exhibited robust physical interaction with NCOA7, which was identified as a key target in both the CUT&RUN and scRNA‐Seq datasets (Figures S30B,C, Supporting Information). NCOA7 strongly interacted with H3K27ac, suggesting that NDST3 may indirectly influence H3K27 acetylation through its interaction with NCOA7 (Figures S30 B,C, Supporting Information).

To explore protein–protein interactions in more detail, we performed in silico simulations using PyMOL and established modeling tools. In parallel with the Co‐IP experiments, this indicated a strong binding affinity between NDST3 and NCOA7, and between NCOA7 and H3K27ac (Figure 5K). Collectively, these results suggest that NDST3 functions as an epigenetic reprogramming modulator that acts through NCOA7 to recruit H3K27ac and facilitate chromatin remodeling and transcriptional resetting.

To examine whether NDST3 modulates transcription factors, we performed motif enrichment analysis on CUT&RUN data by comparing the NDST3‐treated and 6‐OHDA‐induced PD groups. Motif enrichment analysis revealed that the top 10 significantly enriched motifs in the NDST3‐treated PD group corresponded to transcription factors and co‐activators that were potentially regulated by NDST3. Several identified motifs have been linked to transcription factors critical for neuronal development, differentiation, and repair, such as *Etv4*, *Emx2*, *Foxa3*, and *Atoh1*.^[^ ^46^, ^47^, ^48^, ^49^ ^]^ These results suggest that NDST3 may orchestrate a broader transcriptional regulatory network than Ncoa7 (Table 2, Supporting Information).

**Figure 6:**
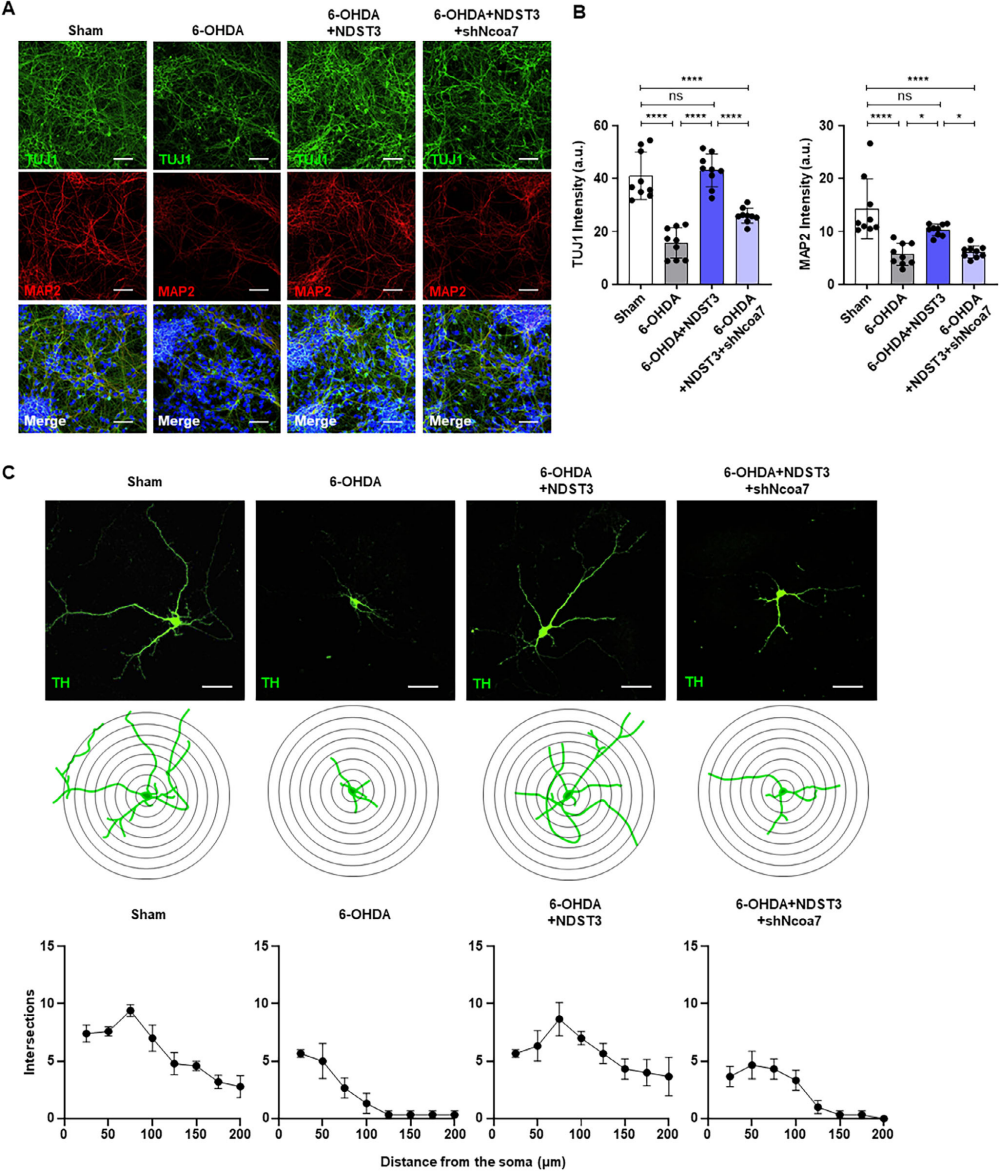
Epigenetic regulators mediate the restoration of damaged dopaminergic neurons. A) Immunofluorescence images showing TUJ1‐ and MAP2‐positive cells under each condition. Scale bar = 50 µm. B) Quantification of TUJ1 and MAP2 intensity is shown in Figure 6A. The data are presented as mean ± SEM (*n* = 9 wells per group). One‐way ANOVA with Tukey's multiple comparisons test. *p < 0.05 and ****p < 0.0001. C) Immunochemistry and Sholl analysis of TH‐labeled neurons, accompanying morphology for individual neurons (Top and middle panel). Scale bar = 50 µm. Sholl analysis indicating the number of intersections from the distance to soma (Bottom panel). The data are presented as mean ± SEM (*n* = 3–5 cells per group).

Given Ncoa7's role in transcriptional regulation, we hypothesized that it may function as a key epigenetic mediator of NDST3, facilitating spontaneous cellular maintenance in a damaged PD environment. To examine the role of NCOA7 in the NDST3‐mediated repair mechanism, we employed shRNA‐mediated knockdown of *Ncoa7* in neuronal cultures and confirmed the knockdown efficiency through RT‐qPCR and western blot analyses (Figure S31, Supporting Information). Strikingly, the loss of *Ncoa7* markedly diminished the restorative effects of NDST3, as evidenced by the impaired DN morphology and decreased dendritic intersections (**Figure** **6**A–C). These findings suggest that NDST3 mediates its epigenetic action partly through NCOA7 to enhance H3K27ac levels, activating cellular repair and maintenance pathways essential for DN recovery in PD.

NDST3 is characterized by N‐deacetylase/N‐sulfotransferase activity during heparan sulfate (HS) biosynthesis.^[^ ^50^, ^51^ ^]^ To determine whether the epigenetic function of NDST3 operates independently of its enzymatic activity, we treated NDST3 with small‐molecule inhibitors of heparan sulfate biosynthesis, specifically sodium chlorate and mitoxantrone.

The NDST3‐mediated enhancement of H3K27ac levels and the restoration of the DA neuronal marker Th were maintained despite these inhibitory treatments, showing no significant difference compared to NDST3 treatment alone (Figures S32A–D, Supporting Information). Furthermore, the enrichment of H3K27ac at the upstream region of the *Th* gene TSS remained unaltered under these inhibitory conditions (Figure S32E, Supporting Information). This finding supports the notion that NDST3 epigenetic activity is independent of its enzymatic function in HS biosynthesis.

### 2.7 Efficacy of ICM‐Administered NDST3 in the PD Model

To explore the translational potential of NDST3 as a cell‐resetting factor, we extended our investigation using a more clinically feasible PD gene therapy method, the intracisternal magna (ICM) method, as an alternative to the intraregional injection into the midbrain (Figure S33, Supporting Information). Remarkably, ICM delivery of NDST3 resulted in therapeutic outcomes comparable to those achieved by direct brain injection. NDST3 administered via the ICM route effectively ameliorated Parkinsonian symptoms, improved behavior, and facilitated the restoration of both pathological and molecular features characteristic of PD (Figures S33B–F, Supporting Information). The ICM method, which involves injection into the cerebrospinal fluid, offers a less invasive route and allows for the broad distribution of therapeutic agents throughout the central nervous system.^[^ ^52^, ^53^ ^]^

To ensure the safety of the ICM delivery strategy, particularly regarding potential off‐target effects on non‐DN and peripheral tissues, we performed a comprehensive toxicity assessment after systemic NDST3 administration. Histological evaluation using hematoxylin and eosin (H&E) staining was performed on various organs, including the liver, kidney, heart, lungs, brain, spinal cord, and spleen. Histological examination revealed no treatment‐attributable pathological abnormalities, such as inflammation, necrosis, or fibrosis, in the assessed tissues (Figure S34, Supporting Information). These findings indicate minimal off‐target toxicity and support the safety of ICM delivery. Collectively, this strategy not only demonstrates robust therapeutic efficacy but may also significantly enhance the feasibility and acceptance of NDST3‐mediated gene therapy for PD in future clinical applications.

## 3 Discussion

**Figure 7:**
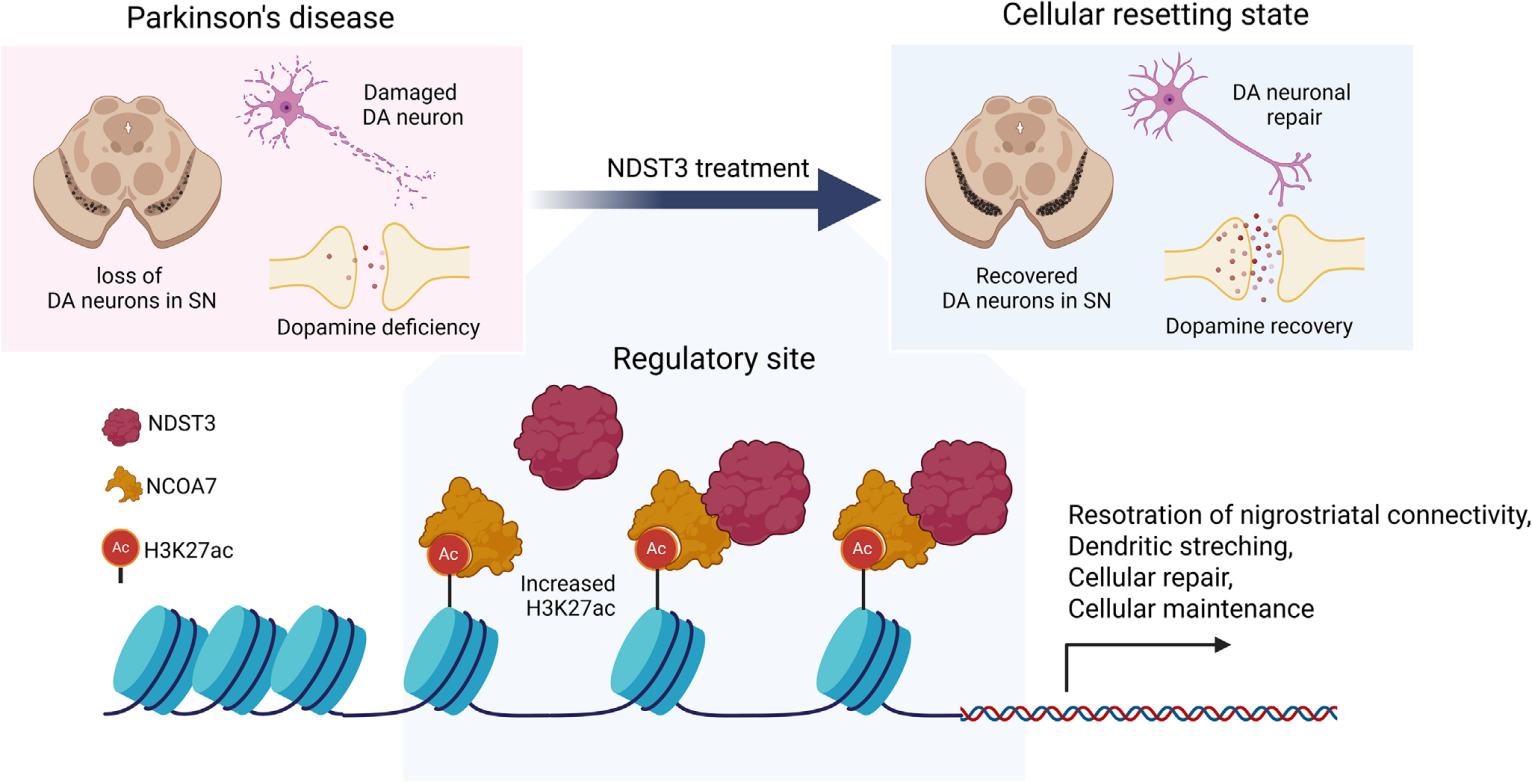
Overview of cell resetting mechanism in PD. Schematic illustration created with Biorender.com.

In this study, we introduce the concept of “Cell‐Resetting,” defined as a therapeutic factor that restores diseased cells to a normal state rather than merely compensating for lost function (**Figure** **7**). Here, we present NDST3 as a resetting factor in the context of PD and demonstrate how it can restore diseased neuronal cells.

Our two‐stage discovery pipeline was designed to enhance the relevance of NDST3 as a therapeutic target. First, using human midbrain organoid cultures, we performed an unbiased proteomic screen that identified NDST3 as the top hit among factors supporting neuronal health (several metabolic and epigenetic regulators were also identified in this screen; Figure S1, Supporting Information). Second, transcriptomic validation in PD mouse models confirmed that NDST3 treatment broadly reversed disease‐related changes in gene expression in vivo. By demanding efficacy across both proteomic and transcriptomic platforms, this pipeline minimized false positives and allowed us to identify NDST3 as a genuine modifier of PD. This cross‐platform, cross‐species strategy (from human organoids to mouse models) bolstered our confidence in NDST3's biological relevance and therapeutic tractability.

In the PD models, NDST3 treatment produced robust neurorestorative effects, revitalized DN, and improved motor function. For instance, NDST3‐treated mice exhibited significantly higher numbers of TH‐positive neurons and near‐normal restoration of nigrostriatal connectivity compared to untreated PD mice (Figure 2). Surviving DN in NDST3‐treated brains also developed more extensive dendritic stretches (indicative of structural regeneration) and showed a greater density of dopaminergic fibers (reflected by increased dopamine transporter labeling), signifying the restoration of nigrostriatal projections. These anatomical improvements translate into functional recovery. NDST3‐treated PD mice performed substantially better on motor behavioral tests, making fewer errors on a balance beam and hanging for longer on a wire‐hang test, approaching the performance levels of healthy controls (Figure 3).

Transcriptomic analyses revealed that NDST3 treatment largely normalized the gene expression profiles of PD neurons, shifting them closer to those of healthy control neurons. NDST3‐treated samples clustered closely with sham‐treated controls in global RNA‐Seq analyses, and numerous genes that were downregulated in the diseased state (including dopaminergic and synaptic genes) were reactivated in response to NDST3 (Figure 4). At the epigenetic level, NDST3 increases histone H3K27 acetylation at the promoters of key genes (e.g., *Ncoa7*), reflecting a shift toward an open chromatin state conducive to neuronal survival. Collectively, these multilevel molecular changes illustrate NDST3's unique ability to “reset” diseased neurons to a more resilient and healthy state. This approach contrasts with conventional PD therapies that focus on supplementing lost dopamine or replacing cells and represents a paradigm shift toward regenerating the patient's own neurons in situ.

Bulk RNA‐Seq profiling revealed that the NDST3‐treated PD group clustered almost indistinguishably from the sham controls, suggesting nearly complete transcriptomic rescue. Additional validation experiments were performed to ensure that this apparent recovery was genuine and not an experimental artifact. Single‐cell RNA‐Seq confirmed that NDST3 exerts a restorative influence at the cellular level of individual cells. The dopaminergic neuron cluster (Cluster 4) from NDST3‐treated mice showed elevated gene expression signatures, in contrast to the markedly degenerated profiles observed in untreated PD mice. Trajectory analysis further showed that NDST3‐treated neurons followed a distinct recovery trajectory rather than the degenerative path observed in PD controls. Consistently, immunohistochemical analysis of midbrain tissue verified the upregulation of representative dopaminergic markers, such as TH, DAT, and GIRK2, in NDST3‐treated PD mice, in line with the sequencing results. In addition, ChIP‐qPCR confirmed that NDST3‐treated mice had elevated levels of H3K27ac at the Ncoa7 promoter region. Furthermore, co‐immunoprecipitation experiments revealed that NDST3 was physically associated with the transcriptional coactivator NCOA7 in NDST3‐treated brains.

Crucially, knockdown of *Ncoa7* abolished NDST3's beneficial effects, confirming that NDST3's ability to normalize the transcriptome is mediated by NCOA7 and associated chromatin remodeling. In silico protein–protein docking analysis further supported a direct NDST3–NCOA7 interaction, reinforcing this mechanism. Consistent with a chromatin‐mediated mode of action, NDST3‐treated neurons showed increased chromatin accessibility in numerous gene regulatory regions (as evidenced by ATAC‐seq), indicating a genome‐wide relaxation of the epigenetic constraints. PD and aging brains exhibit aberrant patterns of histone acetylation (e.g., misallocated or globally elevated acetylation marks). By refocusing histone acetylation on pro‐survival gene loci, NDST3 may counteract the epigenetic dysregulation. These findings support the interpretation that the convergence of NDST3‐treated transcriptomic profiles with those of healthy sham mice reflects a genuine restoration of normal gene programs, rather than an experimental artifact.

We acknowledge the limitations of the toxin‐based PD models used in this study and clarify why the NDST3 dosing schedules differed. The 6‐OHDA lesion model causes rapid and extensive dopaminergic neuron loss without progressive pathology, whereas systemic MPTP intoxication results in a more gradual dopaminergic decline. Neither does toxin recapitulate human PD pathology, such as Lewy body formation; however, each model has complementary aspects of the disease. Accordingly, we administered NDST3 shortly after lesion induction in the 6‐OHDA model to rescue acute cell loss. In the MPTP model, NDST3 was delivered at a later time point to target the chronic degeneration phase. This experimental design ensured that NDST3 was tested in both acute injury and progressive chronic contexts. Despite the divergent nature of these models, NDST3 was effective in both, suggesting that its therapeutic action is robust across distinct paradigms of PD. For example, in MPTP‐treated mice, NDST3 treatment elevated nigral TH levels and significantly ameliorated motor deficits (Figure 3), highlighting the broad efficacy of this factor.

Mechanistically, NDST3 mediates its neurorestorative effects primarily through chromatin remodeling, predominantly by interacting with NCOA7 and subsequently regulating histone acetylation. In NDST3‐treated mice, Ncoa7 in the promoter region was highly enriched compared to that in the vehicle control, indicating that NCOA7 was induced by NDST3 (Figure S30, Supporting Information). Moreover, NDST3 physically interacted with NCOA7, as demonstrated by co‐immunoprecipitation of treated samples. NDST3 has recently been identified as a histone deacetylase that acts on α‐tubulin in the cytosol. However, in the nucleus, the NDST3–NCOA7 partnership promotes increased histone acetylation, highlighting a context‐dependent functional shift that is highly relevant to neurodegeneration. This NDST3–NCOA7 collaboration likely underlies the observed increase in H3K27ac levels at dopaminergic gene loci in NDST3‐treated neurons. Consistent with this epigenetic activation, NDST3‐treated neurons also exhibited greater chromatin accessibility in the regulatory regions, which was aligned with increased H3K27ac marks. Correspondingly, silencing *Ncoa7* negated NDST3's neurorestorative effects, demonstrating that NCOA7 is an essential mediator of NDST3 chromatin‐driven gene activation. Taken together, these results indicate that NDST3 “resets” neurons by recruiting NCOA7 to enhance the transcription of pro‐survival and repair pathways, revealing a novel epigenetic mechanism underlying neuronal recovery in PD.

NDST3 overexpression in wild‐type mice did not produce detectable toxicity or behavioral deficits. NDST3‐treated healthy mice performed normally in all motor tests and showed no abnormal histopathology, neuroinflammation, or off‐target gene expression compared with the controls. They exhibited neither weight loss nor other adverse effects. These observations suggest that NDST3 does not disrupt normal physiology and remains largely inert under baseline conditions, becoming active only in the context of neurodegenerative pathology. This selectivity is advantageous for therapeutic development, indicating a favorable safety profile and a wide therapeutic window for NDST3.

PD is not solely a neuron‐autonomous disease; glial and immune cells also contribute to neurodegeneration. Therefore, the effects of NDST3 are likely to extend to these cell types. In NDST3‐treated PD mice, microglia and astrocytes exhibited reduced levels of inflammatory markers and increased expression of neurotrophic or supportive genes compared with untreated PD mice. This shift indicates a more neurosupportive glial phenotype, consistent with the capacity of glia to adopt either pro‐ or anti‐inflammatory states in PD. Consistently, protein network analysis of NDST3‐regulated targets (STRING) showed significant enrichment of immune regulation and extracellular matrix pathways (Figure S24, Supporting Information). This finding suggests that NDST3 fosters a more permissive neuroenvironment, potentially by dampening harmful inflammation and modulating the recruitment of immune cells. Thus, NDST3's overall therapeutic effects are likely to involve both direct neuronal rescue and indirect immunomodulation. NDST3 not only acts on neurons intrinsically but also shifts glial cells toward neuroprotective phenotypes and attenuates inflammatory processes, improving the overall environment for neuron survival.

NDST3's robust efficacy and multimodal mechanism of action highlight a promising new therapeutic strategy for PD. By epigenetically reprogramming neurons to a healthier state, NDST3 addresses the underlying drivers of the disease rather than alleviating symptoms. From a translational standpoint, NDST3 can be delivered using a viral vector or by intrathecal infusion. In support of the latter route, we found that NDST3 administered into the cerebrospinal fluid (via intracisternal magna injection) was dispersed widely throughout the brain and alleviated neuropathology (Figure S33, Supporting Information). Intrathecal infusion methods have already been used in patients, underscoring the feasibility of this approach for the clinical delivery of NDST3. Moreover, NDST3's selective activity in diseased versus healthy tissues suggests that it can be administered early in the disease course to prevent neurodegeneration without causing off‐target effects. Furthermore, the “cell‐resetting” therapeutic paradigm exemplified by NDST3 may extend to other neurodegenerative disorders. Intriguingly, NDST3 expression is reduced in patients with several other neurodegenerative diseases (e.g., Alzheimer's disease, amyotrophic lateral sclerosis, and frontotemporal dementia), suggesting that augmenting NDST3 or similar factors may confer neuroprotective benefits beyond PD. Taken together, these findings suggest that NDST3 is a compelling therapeutic candidate for PD and illustrate the potential of epigenetic cell‐resetting strategies for combating neurodegeneration. Future studies are important to evaluate NDST3's long‐term effects and efficacy in higher‐order models; however, our current results lay a strong foundation for its clinical development.

## 4 Experimental Section

### Proteome Sample Preparation for Quantitative Mass Spectrometry (MS) Analysis

Each sample was retrieved from the respective culture system and pelleted by centrifugation (500 ×g for 3 min at 4 °C), and the supernatant was carefully removed. Each sample was frozen and stored in −80 °C until further protein sample preparation steps for MS analysis. Cell lysis buffer, 8 m urea with 50 mm ammonium bicarbonate buffer at pH 8.5 (Sigma–Aldrich, U5378 and 09830) was added to every sample used for quantitative MS analysis simultaneously. The samples were then sonicated using Q800R3 Sonicator (Qsonica, Q800R3‐110) at an amplitude of 60 % for 30 min with a pulse rate of 15 s on and 15 s off. BCA protein assay was performed on each sample to measure the protein amount within each sample. The protein concentration was determined utilizing Pierce BCA protein assay kits (Thermo Fisher Scientific, 23227). The dithiothreitol (DTT) (Sigma–Aldrich, 43815) was added to each sample to make a final concentration of 10 mm DTT followed by 37 °C 1 h incubation. The iodoacetamide (Sigma–Aldrich, I1149) was added to each sample to make a final concentration of 40 mm followed by 37 °C 1 h incubation in the dark. 50 mm ABC buffer was added to each sample to make the final concentration of urea 1 m. Trypsin (Thermo Fisher Scientific, MS grade, 90057) was added to a concentration 2 %, trypsin weight/ protein weight, and incubated at 37 °C for 16 h. Trifluoroacetic acid (TFA) (Sigma–Aldrich, 80457) was added to each sample to make 0.4% TFA, and a desalting step was performed utilizing C18 SPE cartridge (Sigma–Aldrich, 52601‐U) following the manufacturer's protocol. Desalted samples were eluted in 80 % acetonitrile (ACN) (Sigma–Aldrich, 100029) with 0.1% TFA in water (Sigma–Aldrich, 1153334000). Each sample was dried to completion using a concentrator (Eppendorf) 50 mm HEPES (pH 8.0) buffer (Sigma–Aldrich, 54457) was added to each sample. The peptide amount was measured using BCA protein assay kit, and the same amount of peptide was used for isobaric tagging with TMTpro 16plex label reagents (Thermo Fisher Scientific, A44521) following the manufacturer's protocol. Tagged samples were combined, and a desalting step was performed using C18 SPE cartridge (Sigma–Aldrich, 52601‐U) and reconstituted in 10 mm ABC buffer. The samples were fractionated by micro‐scale fractionation system based on Triversa Nanomate (Advion) as described in Yeon et al.^[^ ^54^ ^]^ The fractionation was performed using in‐house prepared capillary column, with 320 µm i.d. and length of 55 cm packed with Jupiter 3 µm C18 beads (Phenomex, 04A‐4263). Fractionation was based on a linear gradient with solvent A (10 mm ABC in water) and solvent B (10 mm ABC in 90 % ACN Applied gradient was isocratic 2 % solvent B from 0–14 min, solvent B was increased from 2% to 10 % during the next 2 min, and increased from 10% to 40 % for following 100 min. Eluting samples from the column were automatically concatenated into 24 fractions, and each fraction was dried to completion and reconstituted with 50 mm ABC buffer for LC‐MS/MS analysis.

### Liquid Chromatography and Tandem MS Analysis

Peptide separation and tandem MS analysis were done by Orbitrap Eclipse (Thermo Fisher Scientific) coupled to the nanoAcquity UPLC system (Waters). Liquid chromatography was performed with in‐house prepared columns, all of which were packed with 3 µm Jupiter C18 particles (Phenomenex, 04A‐4263). The analytical capillary column length of 100 cm and 75 µm i.d., and the trap column length of 3 cm and 150 µm i.d. The column temperature was maintained to be 45 °C with the column heater (Analytical Sales and Services, H‐2004C) during the entire run. A flow rate of 300 nL min^−1^ was applied to the UPLC system. Solvent A (0.1 % formic acid (Thermo Fisher Scientific, 28905) in water (Sigma, 1153334000)) and solvent B (0.1 % formic acid in ACN) was used with linear gradient that was set as follows: solvent B was raised from 5 % to 10 % for 0 to 5 min, solvent B was then raised from 10 % to 30 % for the next 200 min. The mass spectrometer was operated on synchronous precursor selection (SPS)‐MS3 mode with a total run time of 250 min. MS acquisition was done with following set up: MS1 ‐ 120K orbitrap resolution, 400 K auto gain control (AGC) target value, 30 % radio frequency (RF) and auto was used for maximum injection time setting; MS2 ‐ 15K orbitrap resolution, AGC target value was 50 K, 30 % HCD collision energy and auto was used for maximum injection time; MS3 ‐ 30K orbitrap resolution, AGC target value was 250 K, 55 % HCD collision energy and maximum injection time was 54 ms with enhanced resolution mode for TMT and TMTpro reagents (Turbo TMT). The mass spectrometry proteomics data were to be deposited in the ProteomeXchange Consortium via the PRIDE partner repository.^[^ ^55^ ^]^

### MS Data Processing and Proteome Identification and Quantification

Protein identification and quantification were performed with the Proteome Discoverer platform, version 2.1 (Thermo Fisher Scientific), using UniProt human reference proteome UP000005640 (last modified on November 25, 2020). The search and quantification were performed with the default processing workflow for TMTpro SPS MS3 analysis and the default consensus workflow for MS3 reporter quantification. Sepctra were searched for fully tryptic peptides with static modification for TMTpro on N‐terminal and lysine at 1 % false discovery rate at the protein level. Unique and razor peptides were used for quantification, and the threshold for co‐isolation was 50, and threshold for average reporter signal to noise was 10, and the SPS mass matches threshold of 65 %. Protein identification and scaled abundance obtained from the Proteome Discover (Thermo Fisher Scientific) search for each protein based on its TMTpro reporter ion intensity values were utilized for all the following analyses.

### Cell Line

ReNCell cells (Millipore, SCC008) were cultured according to the manufacturer's protocol in ReNCell NSC Maintenance Medium (EMD Millipore, SCM005) supplemented with 20 ng mL^−1^ FGF2 (Peprotech, 100‐18B) and 20 ng mL^−1^ EGF (Peprotech, AF‐100‐15). The cultures were maintained at 37 °C in a humidified incubator with 5 % CO_2._ Medium was replenished every other day, and cells were passaged every 3–4 days using Accutase (Sigma–Aldrich, A6964).

### Primary Dopaminergic Neurons

Midbrain tissues were isolated from E13.5 mouse embryos (Dae Han Biolink Inc.) following extraction from pregnant dams. Embryos were carefully removed from the uterine sacs and decapitated using small sterile scissors. The heads were placed in cold phosphate‐buffered saline (PBS; Hyclone, SH30256.01), and brains were rapidly dissected to isolate the midbrain region. Tissue was finely minced in cold PBS and subsequently digested with TrypLE (Gibco, 12605028) at 37 °C for 20 min. The cell suspension was neutralized in Plating Medium (Neurobasal medium (Gibco, 21103049), 2 % B27 supplement (Gibco, 17504044), 1 % l‐glutamine (Gibco, 25030081), 1 % Penicillin/Streptomycin (Hyclone, SV30010), 10 % Fetal bovine serum (FBS; Gibco, 10100147), Laminin (2µg mL^−1^; Sigma–Aldrich, L2020)). Following filtration through a 0.45 µm cell strainer (BD Falcon), cells were seeded onto poly‐d‐lysine (Sigma–Aldrich, P6407) coated plates. On the following day, the culture medium was replaced with maintenance medium (Neurobasal medium, 2 % B27 supplement, 0.25 % l‐glutamine, 1 % Penicillin/Streptomycin, 1 % FBS, Laminin).

### H_2_O_2_ Exposure

The primary cultured neurons were treated with freshly prepared H_2_O_2_ solution (500 µm) for 24 h. The H_2_O_2_ was prepared by diluting commercial H_2_O_2_ (Sigma‐Aldrich, 95321). Following the treatment, the neurons were washed and further incubated in the neuronal culture medium at 37 °C.

### Small Molecule Treatment

Primary dopaminergic neurons were prepared from the midbrain of E13.5 mouse embryos. The neurons were treated with either sodium chlorate (50 mm; Daejung Chemicals & Metals, 7547‐1405) or mitoxantrone (50 nm; Sigma–Aldrich, M6545) for 24 h. After the treatment, the neurons were washed and further incubated in neuronal culture medium at 37 °C.

### Lentivirus Production

Lentiviruses were generated by HEK293FT cells (Invitrogen, R70007) maintained in Complete medium (DMEM, 10 % FBS, 1 % l‐glutamine, and 1 % P/S). The 2 × 10^7^ cells were plated in a 15 cm culture dish one day prior to transfection. Cells were transfected with a lentiviral construct containing the Lenti‐CMV‐NDST3 vector using calcium phosphate coprecipitation. Medium was changed 24 h post‐transfection, and viral supernatants were collected after 72 h. The supernatants were centrifuged to remove cellular debris, filtered through a 0.45 µm filter, and viral particles were concentrated via ultracentrifugation. The resulting viral pellet was resuspended in cold PBS.

### Adeno‐Associated Virus (AAV) Production

In order to express the human NDST3 (hNDST3) gene using an AAV, the NDST3 gene DNA fragment that was to be amplified by PCR was inserted using infusion cloning (Clontech, In‐Fusion HD Cloning Kit) into the AAV‐CMV‐GFP vector (Addgene, 67634). Then, using a helper‐free HEK293 cell method to generate AAV, the AAV‐CMV‐hNDST3 construct was purified using a capsid from AAV serotype 9. Iodixanol gradient ultracentrifugation was employed in the purifying procedure at the KIST Virus Facility.

### Animal

Adult female ICR mice (CrljOri:CD1 strain from Dae Han Biolink Inc., 20–30 g body weight, 7 weeks old) and C57BL/6 mice (C57BL/6JBomTac strain from Dae Han Biolink Inc., 20–25 g body weight, 7 weeks old) were used for all experiments. All procedures, including housing, surgery, behavioral assays, and euthanasia, were performed in compliance with the Stand Up Therapeutics guidelines. All procedures in mice were performed in compliance with protocols approved by the Institutional Animal Care and Use Committee (IACUC) of Stand Up Therapeutics (23‐IACUC‐001∼006, 24‐IACUC‐005∼006).

### Parkinson's Disease Model

For 6‐hydroxydopamine (6‐OHDA)‐induced PD model, 8‐week‐old ICR mice were randomly divided into treatment groups receiving either saline or NDST3 following a unilateral injection of 6‐OHDA. Anesthesia was induced using tribromoethanol, and animals were positioned in a stereotaxic frame. A 6‐OHDA (10 µg uL^−1^; dissolved in 0.02 % ascorbate in saline) was injected into the substantia nigra over 4 min at a rate of 0.5 µL min^−1^ using a 26‐gauge 10‐µL Hamilton syringe. The stereotaxic coordinates used anteroposterior (AP) −3.1 mm, mediolateral (ML) +1.4 mm, and dorsoventral (DV)−4.4 mm. Five days after the 6‐OHDA lesion, 2 µL of NDST3 was injected into the substantia nigra. Baseline histological screening demonstrated that by day 5 post‐injection, TH‐positive neuronal loss in the SNpc reached a stable level of ≈80 %, consistent with successful PD induction. This was confirmed with DAB staining and quantitative analysis, as presented in Figure S7C,D, Supporting Information. For the MPTP‐induced PD model, C57BL/6 mice were injected intraperitoneal injection (i.p.) with 30 mg kg^−1^ per day MPTP for 8 days or saline once daily for 8 days (Tokyo Chemical Industry, M2690) following the modified protocol.^[^ ^56^, ^57^ ^]^ Control animals received saline injections. Four weeks after the final MPTP injection, 2 µL of NDST3 was injected into the substantia nigra. For CTB injection, ICR mice were anesthetized with tribromoethanol, positioned in a stereotaxic frame, and 1 % CTB (Invitrogen, Alex Fluor 647 Conjugate) was injected into the dorsal striatum for 6 min at a rate of 0.5 µL min^−1^ using a 26‐gauge 10‐µL Hamilton syringe. The stereotaxic coordinates used AP + 0.8 mm, ML + 2.5 mm, DV – 2.2 mm. Experimental validation revealed that maximal dopaminergic neuronal depletion occurs four weeks post‐MPTP treatment, as presented in Figure S11A, Supporting Information.

### Intracisternal Magna (ICM) Injection

Mice were anaesthetized with i.p. injection of tribromoethanol (240 mg kg^−1^). Throughout the procedure, the animal's body temperature was maintained at 37.5 °C using a thermostatically controlled heated blanket. Following the injection, animals were monitored daily, and no post‐surgical neurological abnormalities were observed. Animals were positioned in a stereotaxic frame. A 10 µL‐ Hamilton syringe equipped with a Luer lock needle was gently inserted into the cisternal membrane, ensuring a secure seal. A total of 5 µL of saline or NDST3 (1 × 10^10^ GC head^−1^) was infused into the cisterna magna at a rate of 10 µL min^−1^. To prevent reflux, the needle was left in place for an additional 10 min before withdrawal.

### Real‐Time qPCR Analyses

Total RNA was extracted using RNA extraction Kit (Qiagen, 74106) following the manufacturer's instructions. RNA concentration and purity were assessed using a NanoDrop Spectrophotometer. Complementary DNA was synthesized from 200 ng of total RNA. Reactions were run on QuantStudio 3 (ThermoFisher Scientific), and gene expression levels were quantified using relative Ct normalized to housekeeping controls.

### Immunocytochemistry

Cells fixed with 4% paraformaldehyde (PFA) were permeabilized in 0.1% Triton X‐100 in PBS, followed by blocking with 1% bovine serum albumin (BSA). The cells were incubated with primary antibodies for the neuronal markers TUJ1 (Abcam, ab78078, Lot# 1007761‐10), MAP2 (Cell Signaling Technology, 4542S, Lot# 4), and TH (Merck Millipore, AB152, Lot# 4127053). After overnight incubation, the cells were incubated with appropriate fluorescent secondary antibodies (Invitrogen A11001, Lot# 2318440; Invitrogen, A11012, Lot# 2360065) or horseradish peroxidase‐conjugated secondary antibodies (Invitrogen, 65‐6120, Lot# AB405344) and counterstained with 4′,6‐diamidino‐2‐phenylindole (DAPI). The cells were then visualized under a versatile confocal microscope (Olympus).

### Immunohistochemistry

Brains were collected post‐mortem and fixed via immersion in 4 % PFA for 24 h at 4 °C. The following day, tissue was transferred to PBS and stored until sectioning. Brain slices (40 µm thickness) were prepared using CryoStar NX70 cryostat (Thermo Fisher Scientific). Sections were permeabilized in 1 % Triton X‐100 for 15 min and blocked in 10 % serum for 1 h at room temperature. Slices were incubated with primary antibodies targeting dopaminergic neuron markers TH (Merck Millipore, AB152, Lot# 4127053; Merck Millipore, MAB318, Lot#3990619), GIRK2 (Abcam, ab259909, Lot# GR3401320‐4), NDST3 (Novus Biologicals, NBP2‐19501, Lot# 40723), DAT (Merck Millipore, MAB369) and histone modification marker H3K27ac (Abcam, AB4729, Lot# 1059037‐6). After overnight incubation, sections were incubated with appropriated fluorescent secondary antibodies (Invitrogen, A11034, Lot# 2861864; Invitrogen, A11001, Lot# 2318440; Invitrogen, A11012, Lot# 2360065; Invitrogen, A11005, Lot# 2326075) or horseradish peroxidase‐conjugated secondary antibodies (Invitrogen, 65‐6120, Lot# AB405344; Invitrogen, 31470, Lot# ZH397123) and counterstained with DAPI. The number of positive cells in selected fields was calculated using a versatile confocal microscope (Olympus, CKX53), and independent animal sections were performed.

### Whole Cell Patch‐Clamp Recording of Primary DA Neurons

Electrophysiological recordings were performed on randomly selected primary DA neurons using the whole‐cell patch‐clamp technique. Cells were bathed in artificial cerebrospinal fluid (ACSF) containing (in mm): 124 NaCl, 2.5 KCl, 1.2 NaH_2_PO_4_, 24 NaHCO_3_, 5 HEPES, 12.5 glucose, 2 MgSO_4_, and 2 CaCl_2_ (pH 7.4). Recordings were carried out using a Multiclamp 700B amplifier (Molecular Devices), connected to a DigiDATA interface, and data were acquired with pClamp software (v10.6) at a sampling frequency of 10 kHz and filtered at 2 kHz. Patch pipettes (resistance: 4–8 MΩ) were filled with an internal solution composed of (in mm): 120 K‐gluconate, 10 KCl, 2 Mg‐ATP, 0.5 Na‐GTP, 0.5 EGTA, 20 HEPES, and 10 phosphocreatine (pH adjusted to 7.3). After achieving whole‐cell configuration, the RMP was recorded under current‐clamp mode. To assess passive membrane properties such as input resistance, capacitance, and time constant, recordings were conducted in voltage‐clamp mode with the membrane potential held at –60 mV. The ability of neurons to generate APs was tested by applying 1‐s current injections in 20 pA increments, ranging from –160 pA to +160 pA, under current‐clamp conditions.

### In Vivo Extracellular Recording of SNpc Neurons

Extracellular single‐unit recordings were carried out in SNpc neurons, following previously established protocols.^[^ ^58^, ^59^ ^]^ Mice were anesthetized with urethane (1.5 g kg^−1^, intraperitoneally). To target SNpc neurons, a carbon‐fiber glass microelectrode (resistance 0.4–12 MΩ; Carbostar‐1, Kation Scientific, Minneapolis, MN, USA) was positioned stereotaxically into the ipsilateral SNpc using the following coordinates: AP –3.1 mm, ML +1.4 mm, DV –4.0 to –4.5 mm. Neuronal signals were amplified and band‐pass filtered between 0.1 and 10 kHz (ISO‐80; World Precision Instruments, Sarasota, FL, USA). Spike detection was performed, and events were grouped into 1‐s bins for further temporal analysis. Single‐unit signals were recorded and processed using the CED 1401 Micro3 data acquisition system alongside Spike2 software (Cambridge Electronic Design, Cambridge, UK). Following 10 min stabilization period, neuronal firing activity was recorded for an additional 10 min.

### Ex Vivo Cell‐Attached Recording of Spike Activity in Brain Slices

Recording electrodes were fabricated from borosilicate glass capillaries (resistance 2.5–6 MΩ) and backfilled with a sodium‐based extracellular solution (in mm): 124 NaCl, 2 KCl, 1.25 NaH_2_PO_4_, 26 NaHCO_3_, 1.2 MgSO_4_, and 2 CaCl_2_, titrated to pH 7.4 using KOH. To visualize neurons, differential interference contrast (DIC) microscopy was employed. A slight positive pressure was applied to the recording pipette while approaching the cell, and negative pressure was briefly used to establish a high‐resistance seal (typically 10 MΩ to 1 GΩ) between the pipette and the cell membrane. Spontaneous action potentials were recorded in the cell‐attached configuration using a Multiclamp 700B amplifier (Molecular Devices, Sunnyvale, CA), with data sampled at 10 kHz via an Axon 1550B digitizer and processed using pClamp 10.6 software (Molecular Devices). Although voltage clamp mode was technically used (held at 0 mV), neurons were not actually clamped during recordings. Cell‐attached spike recordings were performed after obtaining a stable firing baseline (5–10 min). Neurons that failed to maintain consistent baseline activity were excluded from further analysis.

### Sample Preparation of Mouse Brain

Collected mouse brain samples were treated according to the previous method.^[^ ^60^ ^]^ Brain samples were transferred to a centrifuge tube and homogenized using a homogenizer by adding 20 mL of distilled water per 1 g of brain sample. Dopamine‐D3 (50 µL) as an internal standard and ice‐cold 250 mm formic acid (100 µL) were added to 100 µL of mouse brain homogenate. After vortexing for 20 s, the mixed samples were centrifuged at 14500 g for 5 min at 4 °C. The supernatant was filtered with 0.2 µm PTFE membrane filter and transferred to an amber colored vial.

### Liquid Chromatography‐Tandem Mass Spectrometry (LC‐MS/MS) Condition

A LC‐MS/MS system consisted of a Shimadzu Nexera LC‐40 series (Shimadzu, Nakagyo‐ku, Kyoto, Japan) coupled to a SCIEX Triple Quad 3500 system (AB Sciex, Concord, Canada). To separate dopamine from matrix interference, chromatographic separation was accomplished by LC combined with a Waters ACQUITY UPLC BEH Phenyl column (100 × 2.1 mm, 1.7 µm). The column temperature was maintained at 40 °C, with a flow rate of 400 µL min^−1^. A 5 µL sample volume was injected into LC‐MS/MS system. The eluents consisted of 0.1 % formic acid in water (mobile phase A) and acetonitrile (mobile phase B). The eluents were applied in gradient as follows: 0–0.5 min, 5 % B; 0.5–0.6 min, 5–99 % B; 0.6–5.0 min, 99 % B; 5.0–5.1 min, 99–5 % B; and 5.1–8.0 min, 5% B. MS detection was carried out by electrospray ionization in positive mode using multiple reaction monitoring (MRM) mode. Quantitative and qualitative MRM transitions were set at 154>137 and 154>91, respectively. Instrument parameters were optimized via direct infusion: ion spray voltage, 5000 V; curtain gas, 20 psi; nebulizer gas, 50 psi; auxiliary gas, 50 psi; temperature, 500 °C; and collision‐activated dissociation gas, 9. Nitrogen served as both the nebulizing and collision gases. Data acquisition and analysis of the MRM chromatogram were performed using Analyst software (v1.7.3).

### Multi‐Omics Sample Preparation

For multi‐omics analysis of ATAC‐seq, scRNA‐seq, RNA‐seq, and CUT&RUN, 8‐week‐old ICR mice were randomly divided into treatment groups receiving either saline or NDST3 following a unilateral injection of 6‐OHDA. Anesthesia was induced using tribromoethanol, and animals were positioned in a stereotaxic frame. A 6‐OHDA (10 µg µL^−1^; dissolved in 0.02 % ascorbate in saline) was injected into the substantia nigra over 4 min at a rate of 0.5 µL min^−1^ using a 26‐gauge 10‐µL Hamilton syringe. The stereotaxic coordinates used AP −3.1 mm, ML +1.4 mm, and DV −4.4 mm. Five days after the 6‐OHDA lesion, 2 µL of NDST3 was injected into the substantia nigra. For spatial transcriptomics (Visium), 8‐week‐old ICR mice were injected 6‐OHDA in the substantia nigra over 4 min at a rate of 0.5 µL min^−1^ using a 26‐gauge 10‐µL Hamilton syringe. The stereotaxic coordinates used AP −3.1 mm, ML ±1.4 mm, and DV −4.4 mm. Five days after the unilateral lesion, 2 µL of NDST3 was injected into the substantia nigra.

### Bulk RNA‐Seq Preparation

Total RNA concentration was calculated by Quant‐IT RiboGreen (Invitrogen, R11490). To assess the integrity of the total RNA, samples are run on the TapeStation RNA screentape (Agilent, 5067‐5576). Only high‐quality RNA preparations, with RIN greater than 7.0, were used for RNA library construction. A library was independently prepared with 1ug of total RNA for each sample by Illumina TruSeq Stranded mRNA Sample Prep Kit (Illumina, Inc., USA, RS‐122‐2101). The first step in the workflow involves purifying the poly‐A‐containing mRNA molecules using poly‐T‐attached magnetic beads. Following purification, the mRNA is fragmented into small pieces using divalent cations at elevated temperature. The cleaved RNA fragments are copied into first‐strand cDNA using SuperScript II reverse transcriptase (Invitrogen, 18064014) and random primers. This is followed by the second strand cDNA synthesis using DNA Polymerase I, RNase H, and dUTP. These cDNA fragments then go through an end repair process, the addition of a single “A” base, and then ligation of the adapters. The products are then purified and enriched with PCR to create the final cDNA library. The libraries were quantified using KAPA Library Quantification kits for Illumina Sequencing platforms according to the qPCR Quantification Protocol Guide (KAPA BIOSYSTEMS, KK4854) and qualified using the TapeStation D1000 ScreenTape (Agilent Technologies, 5067‐5582). Indexed libraries were then submitted to an Illumina NovaSeqX (Illumina, Inc.), and the paired‐end (2×100 bp) sequencing was performed by Macrogen Inc. Adapter sequences and low‐quality bases were removed using Trimmomatic (v0.38) with recommended default settings to retain high‐quality read data. Quality‐trimmed RNA‐seq reads were aligned against the mm10 reference genome using HISAT2 v2.1.0 with default parameters. Transcript assembly and quantification were conducted using StringTie (v2.1.3b), generating gene and transcript‐level expression.

### Volcano Plot

The volcano plot was created using differentially expressed genes from bulk RNA‐seq data with the ggplot2 package in R. The plot visualized −log10(*p*‐value) against fold change, with significance indicated by a red line at −log10(0.05). Fold change values greater than 10 or less than −10 were capped at 10 and −10, respectively, and *p*‐values below 10^−10^ were adjusted to 10^−10^ to maintain a consistent scale for better visualization.

### Validation of Markers

To identify glutamatergic, GABAergic, cholinergic, and dopaminergic pathways, the selection of markers was validated through a review of established literature and pathway databases.^[^ ^61^, ^62^, ^63^, ^64^, ^65^, ^66^ ^]^ KEGG Pathway maps for glutamatergic, GABAergic, and cholinergic synapses (map04724, map04725, map04727, map04728) were analyzed to identify markers associated with neuronal synapses.

### Bulk ATAC‐seq Library Preparation

Bulk ATAC‐seq library were prepared starting with 100 000 cells. Cells were lysed on ice using cold lysis buffer to isolate nuclei. Nuclei were quantified using the LUNA‐FL Automated Fluorescence Cell Counter (Logos Biosystems), and their integrity was confirmed via light microscopy. 50 000 nuclei were used transposition reaction using the Tagment DNA TDE1 Enzyme and Buffer Kit (Illumina) and incubated at 37 °C for 30 min. Following the amplification reaction, DNA was purified with a Qiagen MinElute PCR Purification Kit and amplified using the Macrogen amplification kit. To reduce PCR bias related to GC content and fragment size, the ideal cycle number was determined via a PCR side reaction, where a linear plot of Rn versus cycle number identified the cycle corresponding to 1/4 of the maximum fluorescence intensity. The remaining PCR was performed to the cycle number determined by quantitative PCR (qPCR), and the amplified libraries were purified. Library quantification was carried out using qPCR following the KAPA qPCR Quantification Protocol Guide, while quality was assessed using the Bioanalyzer (Agilent Technologies). The prepared libraries were sequenced on the Illumina NovaSeq platform to generate high‐quality sequencing data.

### ATAC‐seq Data Analysis

Raw sequencing reads were first preprocessed using Trim Galore (v0.6.10) to remove adapter sequences and trim low‐quality bases from both ends. Additionally, low‐quality bases were filtered using a sliding window trimming approach, where any window of size 4 with a mean quality score below 15 was excluded. Reads shorter than 36 bp were also discarded to ensure high‐quality data. Cleaned reads were aligned to the mouse reference genome (mm10) using Bowtie2 (v2.5.1) with the following parameters: –very‐sensitive –no‐discordant –no‐mixed –no‐unal –mm.^[^ ^67^ ^]^ The resulting SAM files were sorted and indexed using SAMtools (v1.17).^[^ ^68^ ^]^ Reads mapped to the mitochondrial genome were removed from the indexed BAM files, and duplicate reads were eliminated using the MarkDuplicates tool in Picard (v2.27.5). To identify accessible chromatin regions, peaks calling was performed with MACS2 (v2.1.1.20160309) using the following command:^[^ ^69^ ^]^ macs2 callpeak ‐t ATAC‐seq.bam ‐g hs –bdg –nolambda –keep‐dup all –broad. The MACS2 algorithm infers the length of ATAC‐seq fragments while accounting for local genomic biases. Peaks overlapping with ENCODE blacklisted regions were removed to reduce false positives. For downstream annotation, the ChIPseeker package (v1.22.1)^[^ ^70^ ^]^ within R (v4.2.2) was used to map the enriched peaks to nearby genes and transcripts, facilitating batch annotation of the peak regions.

### scRNA‐seq Library Preparation

Tissue samples were enzymatically dissociated into a single‐cell suspension using Multi Tissue Dissociation kit 1 (Miltenyi Biotec) in combination with the gentleMACS Dissociator, following the manufacturer's protocol. Post dissociation, myelin debris was removed via Percoll density gradient centrifugation, and red blood cells were eliminated using Red Blood Cell Lysis Solution (Miltenyi Biotec). Cell viability and concentration were assessed using the LUNA‐FL Automated Fluorescence Cell Counter (logos biosystems). Cell processing and quality control were guided by the 10× Genomics Single Cell Protocols Cell Preparation Guide and the Guidelines for Optimal Sample Preparation flowchart (Documents CG00053 and CG000126). Single‐cell libraries were generated using the Chromium Next GEM Single Cell 3′ v3.1 protocol (10× Genomics, CG000315) on the Chromium Controller. Cell suspensions were diluted in nuclease‐free water to target 10 000 cells per sample. The cell mixture was combined with the Single Cell 3′ v.3.1 master mix, loaded alongside Gel Beads and Partitioning Oil into a Chromium Next GEM Chip G. Within each droplet, RNA transcripts from individual cells were reverse‐transcribed and tagged with unique cell‐ and molecular barcodes. Following emulsion disruption, cDNA was recovered and subjected to amplification. The amplified cDNA underwent end‐repair, A‐tailing, and Adapter ligation. After library enrichment via PCR, the resulting libraries were purified and quantified using qPCR according to the KAPA library Quantification Guide. Quality assessment was performed using the 4200 TapeStation (Agilent Technologies). Final libraries were sequenced on an Illumina HiSeq platform (Illumina), following the read configuration specified in the 10× Genomics protocol.

### scRNA‐Seq Data Analysis

Single‐cell gene expression analysis was conducted using Cell Ranger software suite (10× Genomics; v7.0.1). Raw BCL files generated by the Illumina HiSeq X Ten were demultiplexed into FASTAQ files using “cellranger mkfastq” pipeline. These FASTQ files were then processed with the “cellranger count”, which aligned reads to the mouse reference genome (mm10), quantified gene expression using unique molecular identifiers (UMIs), and assigned reads to individual cells based on barcodes. The “cellranger count” step also included initial cell calling, clustering, and computation of differential gene expression. When libraries were sequenced across multiple runs, outputs were merged using “cellranger aggr” to generate a combined count matrix normalized for sequencing depth across runs, thereby minimizing potential batch effects arising from technical variations in sequencing. The aggregated gene‐barcode matrices were imported into Seurat v4.3.0 for downstream analysis. Gene expressed in fewer than three cells, and cells with fewer than 200 detected genes were excluded. To minimize the influence of technical artifacts and multiplets, cells with more than 8000 detected genes or mitochondrial gene content exceeding 10 % were filtered out. This quality control step resulted in a final dataset comprising 25113 genes across 27 497 cells. Gene expression data were log‐normalized and scaled across all cells. Dimensionality reduction was performed using principal component analysis (PCA). To further assess and minimize batch effects, PCA was performed on the integrated dataset, confirming no significant separation driven by sequencing batches or runs (see Figure S18A for PCA visualization). As PCA indicated minimal batch effects, no additional batch correction (e.g., Harmony) was applied to avoid unnecessary data distortion; however, a sensitivity analysis using Harmony integration showed no substantial changes in clustering outcomes. The top statistically significant components were selected for clustering and Uniform Manifold Approximation and Projection (UMAP) embedding. Cluster‐specific marker genes were identified using Wilcoxon rank‐sum test, with the following thresholds: min.pct=0.25, logfc.threshold=0.25, and only positively enriched genes retained. To explore the functional relevance of identified markers, gene set enrichment and functional annotation were performed using g:Profiler tool (https://biit.cs.ut.ee/gprofiler/), separately analyzing gene lists from distinct comparison groups; Leukocytes (Cluster 1) by *Cebpb*, *Wfdc17*, *Plcg2*, and *Ccl9*; Astrocytes (Cluster 2) by *Gfap*, *Aldh1l1*, *Sox2*, *S100b*, *Ndrg2*, *Slc1a2*, and *Slc1a3*; Monocytes (Cluster 3 by *Mrc1*, *C1qc*, *Lyz2*, and *Sash1*; DA neuron/progenitor (Cluster 4 and 8) by *Nr4a2*(*Nurr1*), *Zeb1*, and *Camk4*; Fibroblasts (Cluster 5) by *Acta2*, *Dmd*, *Dstn*, *Col3a1*, and *Col1a2*; Microglia (Cluster 6) by *Aif1*/*Iba1*, *Tmem119*, *Cd68*, *P2ry12*, *Fcrls*, *Sall1*, and *Gpr34*; Endothelial (Cluster 7) by *Acvrl1*, *Cldn5*, *Cd34*, *Cdh5*, and *Pecam1*; Undefined (Cluster 9) by *Bank1*, *Fam107b*, *Satb1*, *Dmxl1*, and *H2 Eb1* (Figures S19 and S20, Supporting Information).

### Batch Effect Assessment

To evaluate whether batch correction was necessary for the integrated 6‐OHDA and 6‐OHDA + NDST3 datasets, a comprehensive batch effect assessment was performed using multiple quantitative metrics and visual inspection of principal component analysis. Data normalization was performed using the NormalizeData function with default parameters (normalization.method = “LogNormalize”, scale.factor = 10 000). Highly variable features were identified using the FindVariableFeatures function, followed by data scaling using ScaleData. Principal component analysis was conducted using RunPCA, and the first 30 principal components were retained for downstream analysis. Several established metrics were employed to quantitatively assess batch mixing. The mixing ratio was computed by comparing inter‐batch distances to intra‐batch distances in PCA space. For computational efficiency, 200 cells were randomly sampled from each batch and calculated pairwise Euclidean distances using the first 30 principal components. The mixing ratio was defined as the ratio of mean inter‐batch distance to mean intra‐batch distance, where values approaching 1.0 indicate perfect mixing and values >1.5 suggest significant batch separation. the Euclidean distance was calculated between batch centroids in the 30D PCA space. Smaller distances indicate better batch integration, with distances >20 considered indicative of substantial batch effects. Batch separation was quantified using silhouette analysis on a subset of 1000 randomly selected cells in the first 10 principal components. Silhouette scores were calculated using batch labels as cluster assignments, where scores approaching 0 indicate good mixing and scores >0.2 suggest problematic batch separation. To assess batch effects across individual principal components, two‐sample *t*‐tests were performed comparing 6‐OHDA and 6‐OHDA+NDST3 cells for each of the first 10 principal components. *p*‐values were calculated and transformed to −log10 scale, with values >2 (corresponding to *p *< 0.01) considered indicative of significant batch effects in that component. PCA plots were generated showing the first two principal components (PC1 versus PC2) with cells colored by batch identity (6‐OHDA versus 6‐OHDA+NDST3). Visual inspection focused on the degree of batch mixing versus separation, with well‐integrated datasets showing intermingled rather than segregated batch clusters.

### Doheatmap Analysis

An R‐package was used for K‐clustering of scRNA data from PD and PD+NDST3 into 9 groups and calculated the weighted expression levels as the average log2 fold change. Then, the sorted results were extracted by magnitude and the top 1000 entries. Subsequently, visualized the data using the R‐package's doheatmap function.^[^ ^71^ ^]^

### UMAP and Feature Plot Analysis

UMAP and Feature Plot analysis were conducted by merging separate Seurat objects for PD and PD+NDST3 into a single Seurat object to create a comprehensive background map. Subsequently, barcodes for 6‐OHDA and 6‐OHDA+NDST3 were separated for UMAP generation. Clustering utilized K‐clustering, and the RunUMAPfunction was applied with dimsset to 1:10. The Feature Plot for Nurr1 was specified with a minimum expression value of 2.5. The diagrams were created using the ggplot2 package from R. (https://petti‐lab.github.io/scrnaseq.tutorials.github.io/)

### GO Enrichment Analysis

scRNA‐seq data were used to identify biological processes via GO term enrichment. The data were processed with David Bioinformatics Resources, generating a CSV of enriched GO terms. In R, the GO term names were refined for clarity and converted *p*‐values to numeric for visualization. Using ggplot2, a dot plot to display the GO terms was created.^[^ ^72^ ^]^

### Single‐Cell Trajectory Analysis

Clusters identified in single‐cell RNA‐Seq were subjected to trajectory analysis using the Monocle package.^[^ ^73^, ^74^ ^]^ To characterize cellular progression, cells were ordered in pseudotime, and dimensionality reduction was performed through the discriminative dimensionality reduction via learning a tree (DDRTree) method. First, differentially expressed genes that were identified between the disease model group and the NDST3‐treated group with a q‐value < 0.01 were employed for the first round of dimensionality reduction to define the root state. Subsequently, a list of genes in the category of interest was filtered by q‐value < 0.01 of differential expression level, and only genes expressed in > 10 cells were retained for pseudotime definition. The second round of the DDRTree method was then applied to characterize the progression of gene expression patterns.

### Cell–Cell Communication Analysis

Changes in the number of interactions and strength of interactions among different cell types after the drug treatment were evaluated using the CellChat computational framework.^[^ ^75^ ^]^ Interactions between ligands and receptors (L–R pairs) were identified and evaluated for each pair of cell types based on the CellChat mouse database. Statistically significant L–R pairs with permutation test *p* < 0.05 were used for downstream analysis. Increased or decreased ligand–receptor pairs after the drug treatment were further identified by applying Wilcoxon test (*p* < 0.05).

### Visium Spatial Gene Expression and Tissue Optimization

Spatial gene expression analysis was conducted using the Visium CytAssist platform, which transfers spatially resolved gene expression signals from tissue sections to Visium CytAssist Spatial Gene Expression v2 Slides. This system facilitates the transfer of gene expression probes from the tissue onto the Visium slide. Initial tissue slide preparation adhered to the guidelines stipulated in the Visium CytAssist Spatial Gene Expression Tissue Preparation Guide (Document CG000636), covering sectioning, section placement, and RNA quality assessment (RNA Integrity Number, RIN, ≥ 4). Frozen brains were embedded in Optimal Cutting Temperature (O.C.T) compound and cryosectioned at 10 µm thickness. Subsequent procedures employed the Visium Spatial Gene Expression and Tissue Optimization (TO) Slide & Reagent Kits in accordance with the manufacturer's instructions (10x Genomics), utilizing all recommended reagents. The process entailed methanol fixation, H&E staining, imaging, and de‐staining of tissue slides. Post‐destaining, probe hybridization was promptly carried out as per the Visium CytAssist Spatial Gene Expression Reagent Kits User Guide (CG000495). This involved the hybridization of probe pairs to their respective gene targets and subsequent ligation. The ligation products, now single‐stranded, were transferred from the tissue to a Capture Area on the Visium slide within the CytAssist instrument. Following the capture of ligation products, slides were removed, and probe extension was performed to incorporate a UMI, Spatial Barcode, and partial Read1 sequence. Following extension, the products were enzymatically released from the slide, collected, and subjected to qPCR to determine the optimal number of cycles for indexing in the Sample Index PCR step. Final libraries were purified using SPRIselect magnetic beads and quantified via qPCR in accordance with the KAPA qPCR Quantification Protocol Guide. Quality assessment was performed using the 4200 TapeStation (Agilent Technologies). Sequencing was carried out on the NovaSeq platform (Illumina), with read length configurations in accordance with 10× Genomics recommendations for Visium Spatial Gene Expression libraries.

### Preprocessing and Analysis of Spatial Transcriptome Data

Spatial transcriptome datasets were processed using the Space Ranger (10X Genomics, v2.0.1). Raw files generated from the Illumina sequencing platform were demultiplexed into FASTQ files for each sample using the “mkfastq” command. The “count” command was deployed for the analysis of Visium spatial expression libraries. The image processing workflow was bifurcated into two stages: initial alignment of the tissue image using the fiducial grid to ascertain orientation and position, followed by identification of the tissue‐covered region on the slide. Sequencing reads were aligned to the mouse reference genome (mm10‐2020‐A) using the STAR aligner (v2.5.1b), which enabled accurate mapping of spatially barcoded reads. Gene expression was quantified per spot using UMIs and 10x barcodes, yielding a spatially resolved expression matrix. The integration of image processing and gene expression profiling results was visualized on the corresponding tissue image.

### Spatial Transcriptomics Analysis

Spatial transcriptomics data generated on the 10x Genomics platform were analyzed using the Load10x_spatial function within the R programming environment. PCA was conducted employing the SCT method, with dimensions set to 1:30 for neighborhood analysis. Spatial coordinates and expression levels for NDST3 were extracted via the Loupe Browser 7 (10× Genomics) and subsequently coded for visualization in Blender 4.0 using Python scripting. The brain's hemispheres were delineated, necessitating adjustments to the *x*‐coordinate values to reflect this division. Expression levels for each barcode were then visualized using a gradient representation to highlight spatial gene expression patterns.

### Protein–Protein Docking

Sequences and structures for NDST3, NCOA7, and the H3 segment (K27 acetylated) were obtained from UniProt/PDB. In PyMOL, waters and non‐standard hetero atoms were removed, alternate locations were resolved, poorly resolved termini were trimmed, continuity of each chain was ensured, and residue numbering was standardized. The acetyl‐lysine at position 27 was represented as ALY so that downstream tools recognized the modification. Using GalaxyPepDock, the H3K27ac peptide (FASTA with K27→ALY) was docked to NCOA7 treated as the receptor, and selected the top, most converged cluster representative. From that peptide–protein complex, NCOA7 residues having any atom within 6 Å of the peptide were marked as “active” interface residues. Guided protein–protein docking was then performed in HADDOCK, docking NDST3 to NCOA7 with the above NCOA7 actives (passive residues were auto‐assigned by HADDOCK). The best cluster was chosen by HADDOCK score, Z‐score, cluster size, and convergence. To assemble the ternary complex, the two binary solutions were superposed on NCOA7, a duplicate copy was removed, and NCOA7, NDST3, and H3K27ac were kept. The ternary model was refined with HADDOCK's explicit‐solvent “refine‐only” protocol to relieve clashes and optimize packing. All figures and measurements were generated in ChimeraX and PyMOL. Buried surface area was computed as ΣSASA (monomers) – SASA (complex). Atomic contacts were counted for heavy‐atom pairs within 5.0 Å, and hydrogen bonds were defined by donor–acceptor distance ≤3.5 Å and angle ≥120°. Unless otherwise stated, default parameters were used throughout. After HADDOCK refinement, the top 20 clusters exhibited strong convergence to a single binding interface. Interface Root‐Mean‐Square Deviation (RMSD) values clustered within 0.0–1.10 Å (median 1.027 Å), while HADDOCK scores remained consistently favorable (–550.0 to –500.7; median –526.7). Within this tightly converged ensemble, van der Waals interactions were uniformly stabilizing (median –302.1) and electrostatics broadly favorable (median –1446.6), with only weak monotonic variation versus i‐RMSD. Importantly, the fraction of common contacts (FCC) showed a moderate negative association with HADDOCK Score (ρ≈–0.47), indicating that lower‐scoring models share a more consistent interfacial contact pattern. Collectively, these observations support a physically plausible, well‐converged ternary pose dominated by favorable packing supplemented by electrostatic contributions.

### Western Blot Analysis and Co‐Immunoprecipitation Assay (Co‐IP)

Primary dopaminergic neurons were subjected to Co‐IP using an IP Kit (Thermo Fisher Scientific, 26146), following the manufacturer's protocol. Cells were lysed in IP lysis buffer. The lysates were pre‐cleared with protein A/G agarose beads and then incubated with the primary antibody (NCOA7; Invitrogen, PA5‐26543 or NDST3; Novus Biologicals, NBP2‐19501) at 4 °C overnight. Immunocomplexes were captured with protein A/G beads, washed several times with lysis buffer, and eluted with SDS sample buffer by boiling. Eluates were separated by SDS‐PAGE and transferred to nitrocellulose membranes (Cytiva). Membranes were blocked with 5% BSA or non‐fat milk (followed by datasheet) in TBST for 1 h at room temperature, followed by incubation with primary antibodies (NCOA7 (Invitrogen, PA5‐26543); NDST3 (Novus Biologicals, NBP2‐19501); H3K27ac (Abcam, ab4729); and beta actin (Abcam, ab8226)) overnight at 4 °C. After washing, membranes were incubated with HRP‐conjugated secondary antibodies for 2 h at room temperature. Protein bands were visualized using a chemiluminescence imaging system (Azure biosystems, Azure 280).

### Statistical Analysis

All statistical analyses were performed using GraphPad Prism (v8.0). For data pre‐processing, normalization was applied where appropriate. Normality was checked using the Shapiro–Wilk test, and homogeneity of variances was assessed to ensure the assumptions for the parametric test were met. For comparisons between two independent groups affected by a single variable, the unpaired two‐tailed Student's *t*‐test was used. Student's *t*‐test was used to analyze data for immunoblot assay in NDST3 expression and knockdown efficiency of NCOA7. For comparisons among multiple groups, one‐way analysis of variance (ANOVA) followed by Tukey's multiple comparisons test was applied. One‐way ANOVA with Tukey's multiple comparisons test was used to analyze the number of positive cells, frequency of spontaneous firing, dopamine neuronal firing rates, errors per step in the challenging beam traversal test, fall latency in the wire‐hanging test, T‐turn and T‐total in pole test, mRNA expression levels, ipsilateral turns, tail suspension test and relative intensity in western blot analyses. For experiments involving two independent variables, a two‐way ANOVA was performed, followed by appropriate tests. Two‐way ANOVA with Tukey multiple comparisons was used for the analysis of instantaneous firing frequencies and number of APs in primary DA neurons across a range of step current injections, and error count in the challenging beam traversal test. Two‐way ANOVA with Dunnett's multiple comparisons test was used for the analysis of mean firing rate. A chi‐square test was used for the analysis of the proportion of full AP, incomplete AP, and no AP. To ensure sufficient statistical power for behavioral assays and immunohistochemistry, power analysis was performed. The tests achieved high statistical power (1−β > 0.80) with the given sample sizes (*n* = 20–23, depending on the assay) at α = 0.05. The sample size for each analysis is explicitly stated in the figure legends, and *n* refers to biological replicates, such as independent animals or cultures. Data are presented as mean ± standard error of the mean (SEM) or as individual data points. Statistical analyses for multi‐omics and gene expression in human brain tissues were performed using R statistical software (v4.4.2) with the following packages: readxl, ggplot2, effectsize, dplyr, tidyr, cowplot, and openxlsx. For gene expression in human brain tissue, gene expression data for 20 target genes were extracted from normalized datasets. Outliers were identified and removed using the interquartile range (IQR) method with an aggressive threshold (IQR multiplier = 1.0). Data points falling outside Q1 − 1.0×IQR or Q3 + 1.0×IQR were excluded. Differential gene expression between PD patients and control subjects was assessed using the Wilcoxon rank‐sum test (Mann–Whitney U test) for each brain region (caudate and putamen) separately. Cohen's d was calculated to determine the magnitude of differences between groups, with values of 0.2, 0.5, and 0.8 representing small, medium, and large effect sizes, respectively. Statistical significance was set at *p *< 0.05. No multiple comparison correction was applied as each gene was considered an independent hypothesis. The statistical analyses were performed using R statistical software (v4.4.2) with the following packages: readxl, ggplot2, effectsize, dplyr, tidyr, cowplot, and openxlsx. Statistical significance was set at *p* < 0.05, with significance levels indicated as follows: *p < 0.05, **p < 0.01, ***p < 0.001, and ****p < 0.0001. Results with *p*‐values greater than 0.05 were considered not significant and are indicated as such in the figures. Details of sample size were included in figure legends.

## Conflict of Interest

J.Y. is a scientific primary researcher and founder of Stand Up Therapeutics Inc. Y.C., H.I., S.Y., H.S.S., and C.K. are currently employed at Stand Up Therapeutics Inc. Other authors declare that they have no competing interests.

## Author Contributions

Y. C., Y. N. and H. I. contributed equally to this work. J.Y. conceived the study and supervised the project. Y.C. and J.Y. designed the experiments. Y.C. and I.M. performed most in vitro and in vivo experiments with the contribution of H.S.S., C.K., H.Y.K., S.E.L., W.L., Y.H., J.K., W.Y.C., W.S. G.Y., S.Y., G.Y.K., and H.J.P. performed the bioinformatic analyses. Y.C., Y.N., H.I., J.S.K., and J.Y. wrote the manuscript. All authors commented on the manuscript.

## Supporting information



Supporting Information

Supporting Information

## Data Availability

All data needed to evaluate the conclusions in the paper are present in the paper and/or the Supporting Information. Human postmortem RNA‐seq data provided by C.R. Hale are publicly available in the Gene Expression Omnibus (GSE205450). The RNA‐seq, CUT&RUN, ATAC‐seq, scRNA‐seq, spatial transcriptomics, and proteomics data are available to the public on GitHub (https://github.com/STULab911/Epigenetic‐modification‐and‐Parkinson‐s‐disease) and Gene Expression Omnibus (GSE308116). All the original LC‐MS/MS datasets and related identification files used to support this paper have been deposited to the ProteomeXchange Consortium (http://proteomecentral.proteomexchange.org) via the PRIDE partner repository with the dataset identifier, PRIDE: PXD067971, Reviewer Token: ti7UqmDhAuTZ.
