# Supplementary material for: NDST3‐Induced Epigenetic Reprogramming Reverses Neurodegeneration in Parkinson's Disease
Source: Adv Sci (Weinh). 2025 Nov 21;13(14):e07323. doi: 10.1002/advs.202507323 (PMC12970244; doi:10.1002/advs.202507323)
Supplement: Supplementary file 1 — Supporting Information [file ADVS-13-e07323-s001.pdf]

SUPPORTING INFORMATION

NDST3-Induced Epigenetic Reprogramming Reverses Neurodegeneration in Parkinson’s Disease.

Yujung Chang, Yongwoo Na, Hyeonjoo Im, Garam Yang, Seungseon Yang, Hyun Soo Shim, Chunggoo Kim, GyeungYun Kim, Hyeok Ju Park, Hee Young Kim, Seung Eun Lee, Wonwoong Lee, Yoon Ha, Sungho Park, Jieun Kim, Won-Young Cho, Woong Sun, Jong-Seo Kim* and Junsang Yoo*

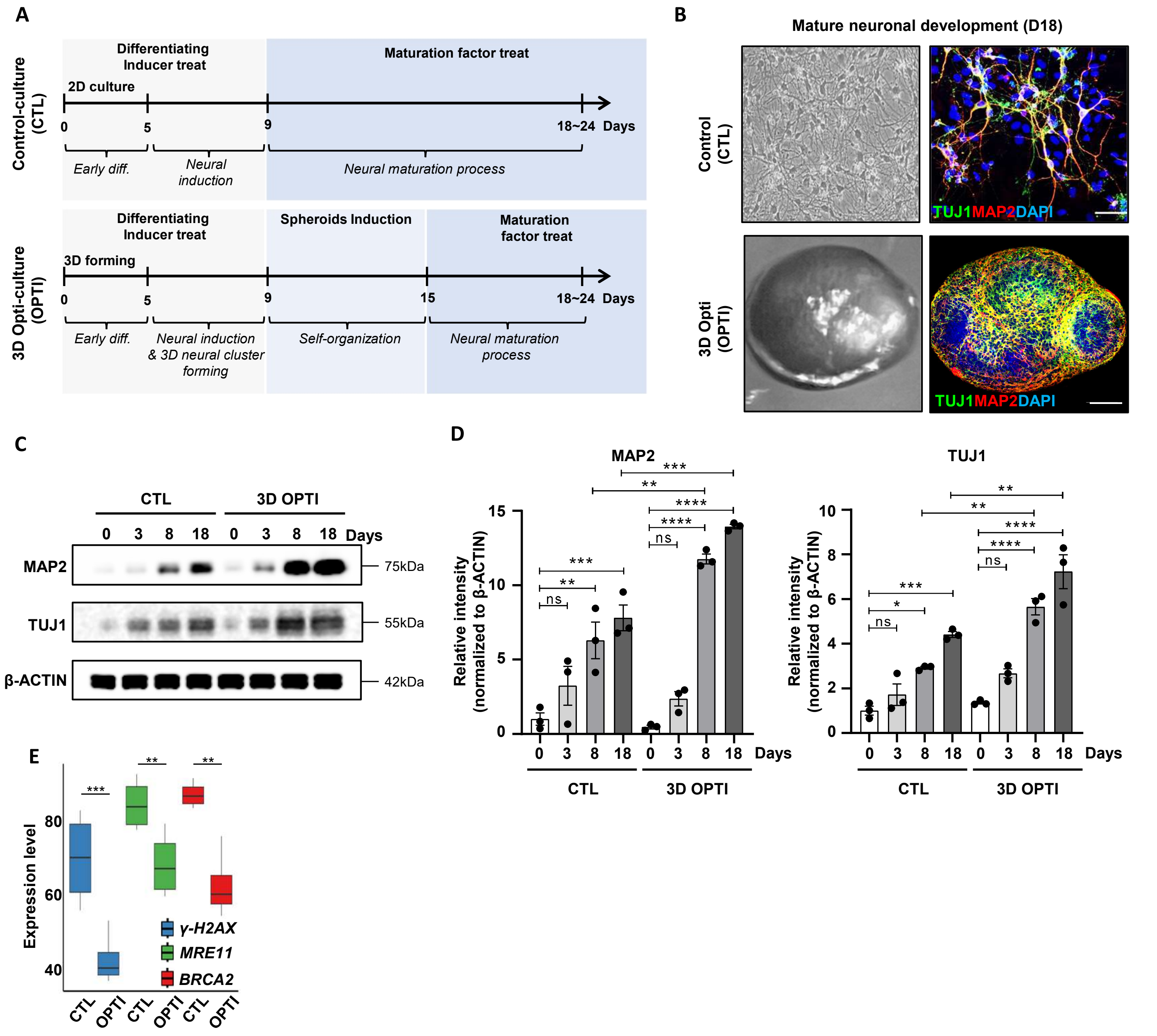

Figs S1. Confirmation of control culture and 3D OPTI-culture. (A) Methodological flow for the screening process under control condition and OPTI-culture. (B) Representative image of TUJ1^{+} and MAP2^{+} cells under control condition and OPTI-culture condition, including bright-field microscopy (Left) and immunofluorescence imaging (Right). (C) Immunoblot analysis showing MAP2 and TUJ1 expression in lysates from CTL and 3D OPTI-culture. Identification of specific MAP2 (75 kDa) and TUJ1 (55 kDa) proteins, with β-ACTIN serving as a loading control. (D) Intensity quantification as shown in Fig S1C. Data are presented as mean ± SEM. (n = 3 wells per group) One-way ANOVA with Tukey's multiple comparisons test. *$p < 0.05$, **$p < 0.01$, ***$p < 0.001$, ****$p < 0.0001$ and ns = not significant. (E) mRNA expression analysis of markers associated with DNA double-strand breaks.

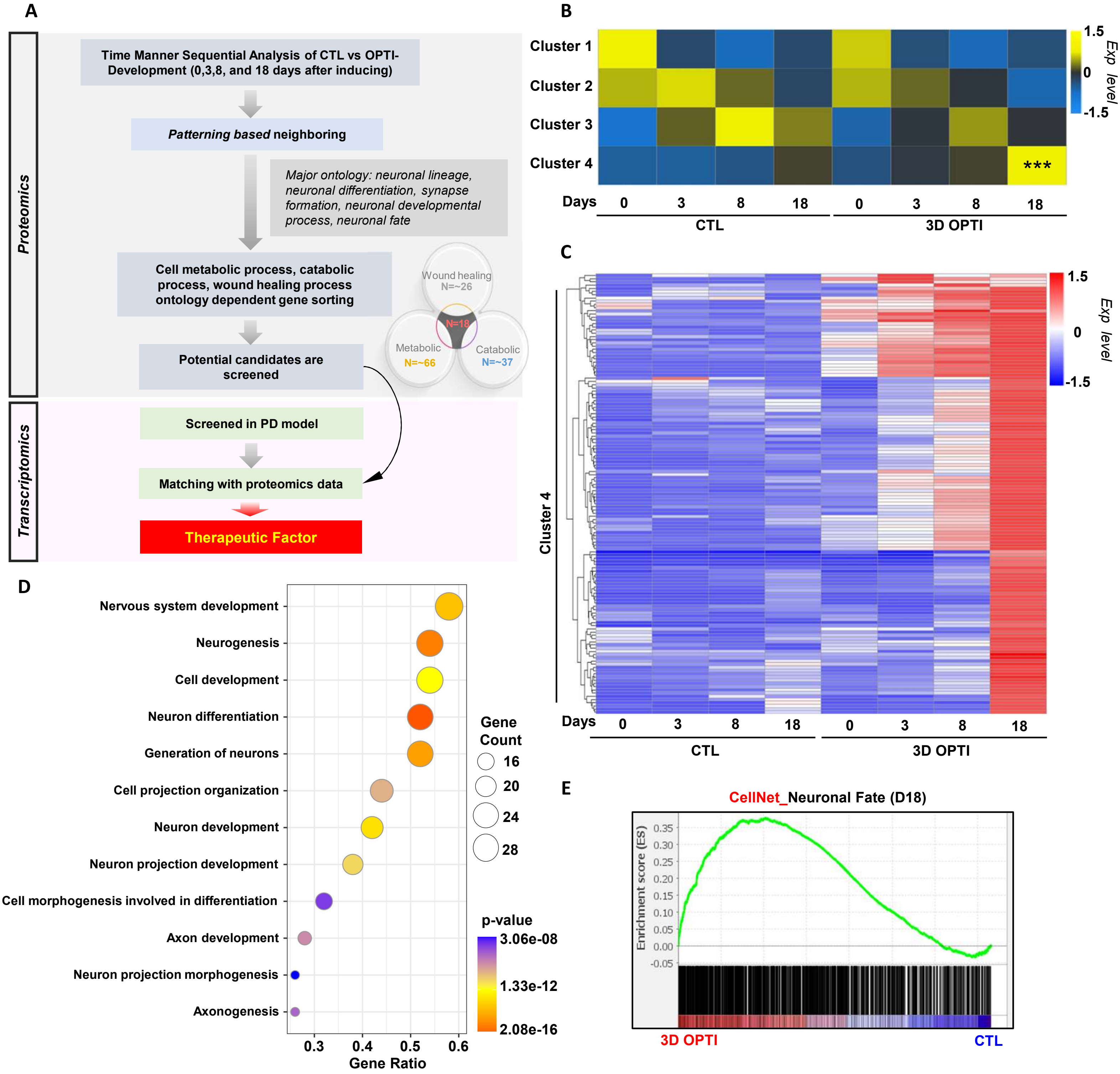

Figs S2. Identification and characterization of epigenetic modulators as regulators of therapeutics genes for Parkinson's disease therapy. (A) Progress in screening methodologies for the selection of therapeutic genes. (B) Heatmap displaying K-means clustering groups of protein expression in differentiated cells under control and OPTI-culture condition. Cluster 4: Statistical significance denoted as ***$p < 0.001$. (C) Heatmap illustrating expression patterns of Cluster 4 in differentiated control and 3D OPTI-culture. (D) Analysis of biological processes from gene ontology analysis with gene sets in Cluster 4. (E) Cell Network-based Gene Set Enrichment Analysis (GSEA) highlighting differential expression patterns between control and 3D OPTI-culture in neuronal differentiation.

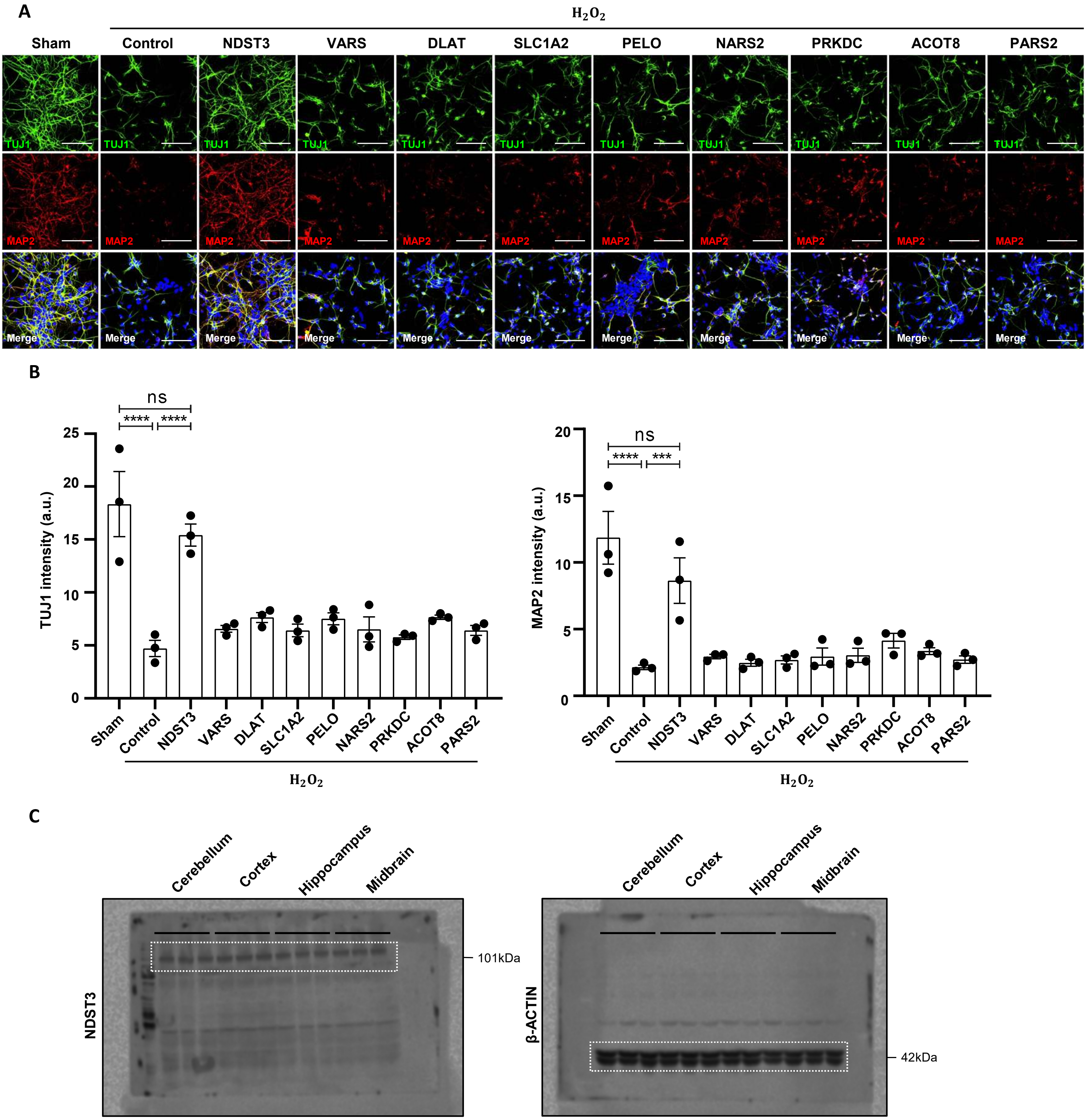

Figs S3. Confirmation of therapeutic candidate factors under H_2O_2 treatment. (A) Representative immunofluorescence images of TUJ1 and MAP2 in the primary neurons. Scale bar = 100 μm. (B) Quantification of TUJ1 and MAP2 intensity shown in Figure S3A. Data are presented as mean ± SEM. (n = 3 wells per group) One-way ANOVA with Tukey's multiple comparisons test. ***p < 0.001, ****p < 0.0001 and ns = not significant. (C) Immunoblot analysis showing NDST3 expression in wild type mouse brain. Identification of specific bands for NDST3 (101 kDa) protein with β-ACTIN serving as a loading control. Uncropped membrane images are shown.

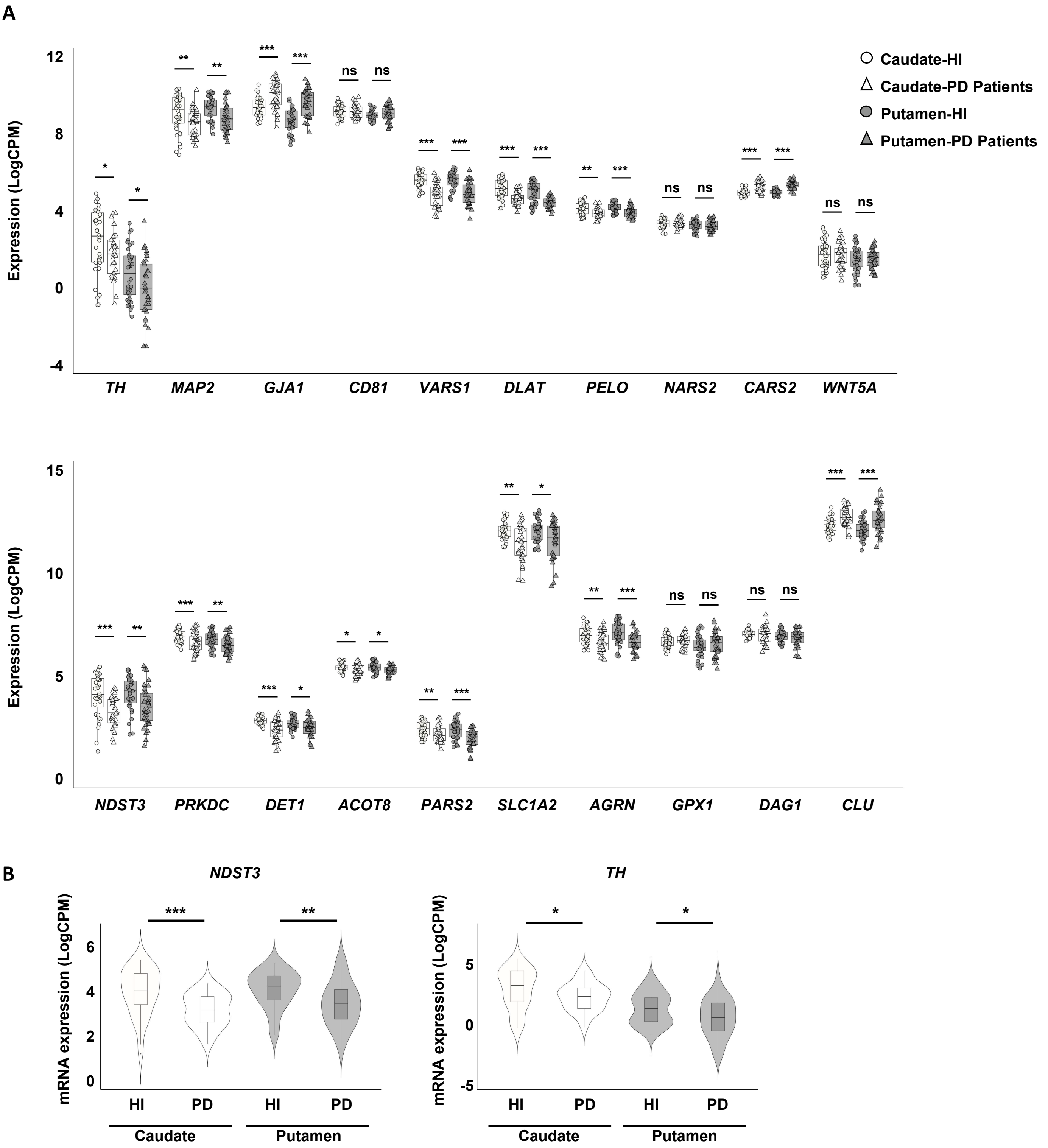

Figs S4. Confirmation of mRNA expression in human patients. (A) mRNA expression analysis of mature DA neuronal factors with 18 candidates that were screened as potential epigenetic regulators in the caudate and putamen regions of healthy individuals (HI) and PD patients. Wilcoxon rank-sum test (n = 29 – 40 individual brain sample per group). *p < 0.05, **p < 0.01, ***p < 0.001 and ns = not significant. (B) mRNA expression analysis of NDST3 and TH in the caudate and putamen regions of healthy individuals (HI) and PD patients. Wilcoxon rank-sum test (n = 31 – 38 individual brain sample per group). *p < 0.05, **p < 0.01 and ***p < 0.001.

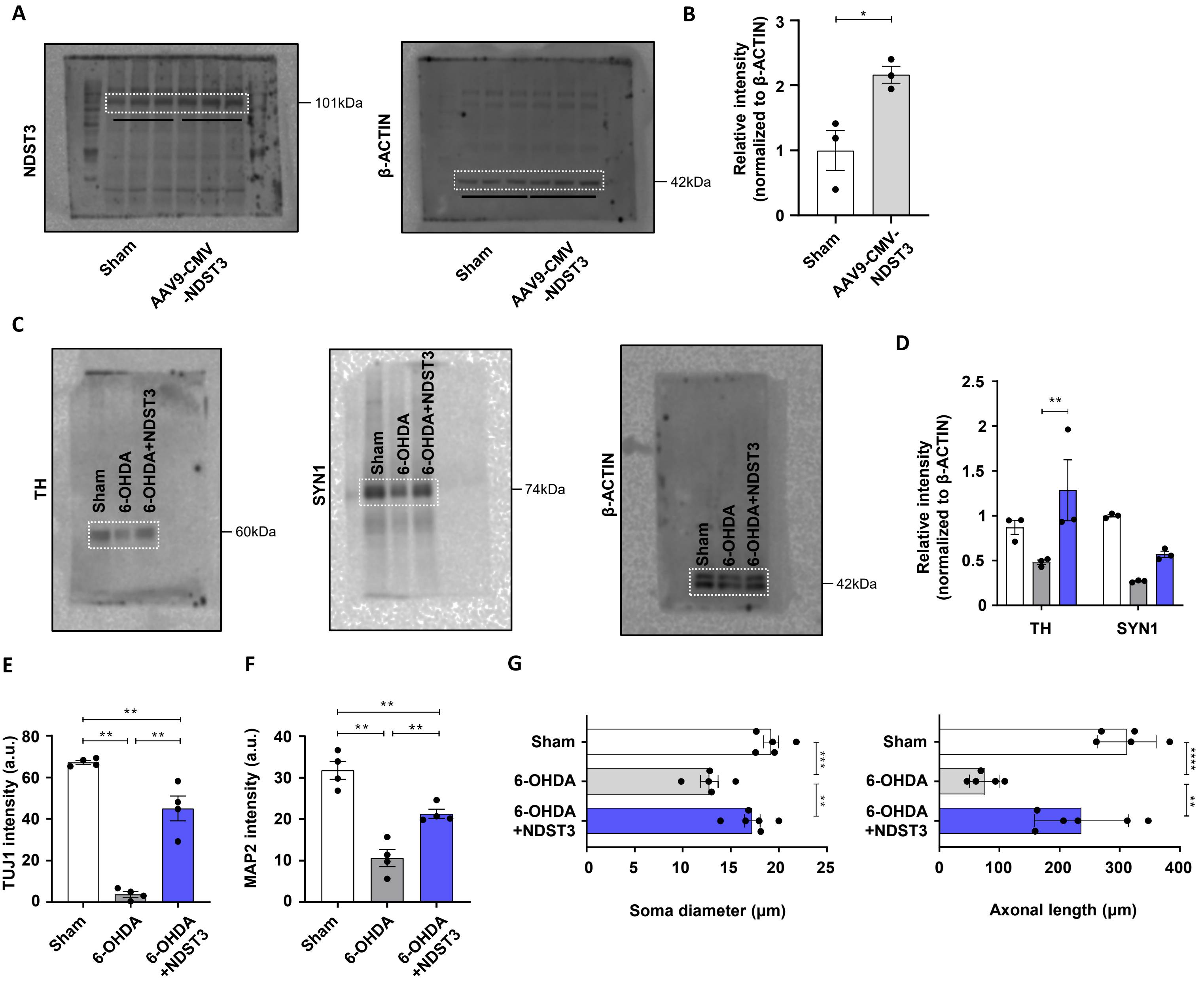

Figs S5. Neuronal marker expression and morphology in 6-OHDA-induced PD model and NDST3 treatment. (A) Immunoblot analysis showing NDST3 expression in primary dopaminergic neuron lysates from sham, NDST3 overexpressed group. Identification of specific bands for NDST3 (101 kDa) protein with β-ACTIN serving as a loading control. Uncropped membrane images are shown. (B) Quantification of the relative intensity of NDST3 as shown in Fig S5A. Data are presented as mean ± SEM (n = 3 wells per group). Student T-test. $^{*}p < 0.05$. (C) Immunoblot analysis showing TH and SYN1 expression in primary dopaminergic neuron lysates from sham, 6-OHDA-induced PD model, and NDST3-treated PD model. Identification of specific bands for TH (60 kDa) and SYN1 (74 kDa) proteins, with β-ACTIN serving as a loading control. Uncropped membrane images are shown. (D) Quantification of the relative intensity of TH and Synapsin1 as shown in Fig S5C. Data are presented as mean ± SEM. (n = 3 wells per group). One-way ANOVA with Tukey's multiple comparisons test. ** $p < 0.01$. (E-F) Intensity quantification of TUJ1 and MAP2 as depicted in Fig. 1D. Data are presented as mean ± SEM. (n= 4 wells per group) One-way ANOVA with Tukey's multiple comparisons test. $^{**}p < 0.01$. (G) Soma diameter and axonal length as in Fig 1E. Data are presented as mean ± SEM (n = 5 – 6 cells per group). One-way ANOVA with Tukey's multiple comparisons test. $^{**}p < 0.01$, $^{***}p < 0.001$, and $^{****}p < 0.0001$.

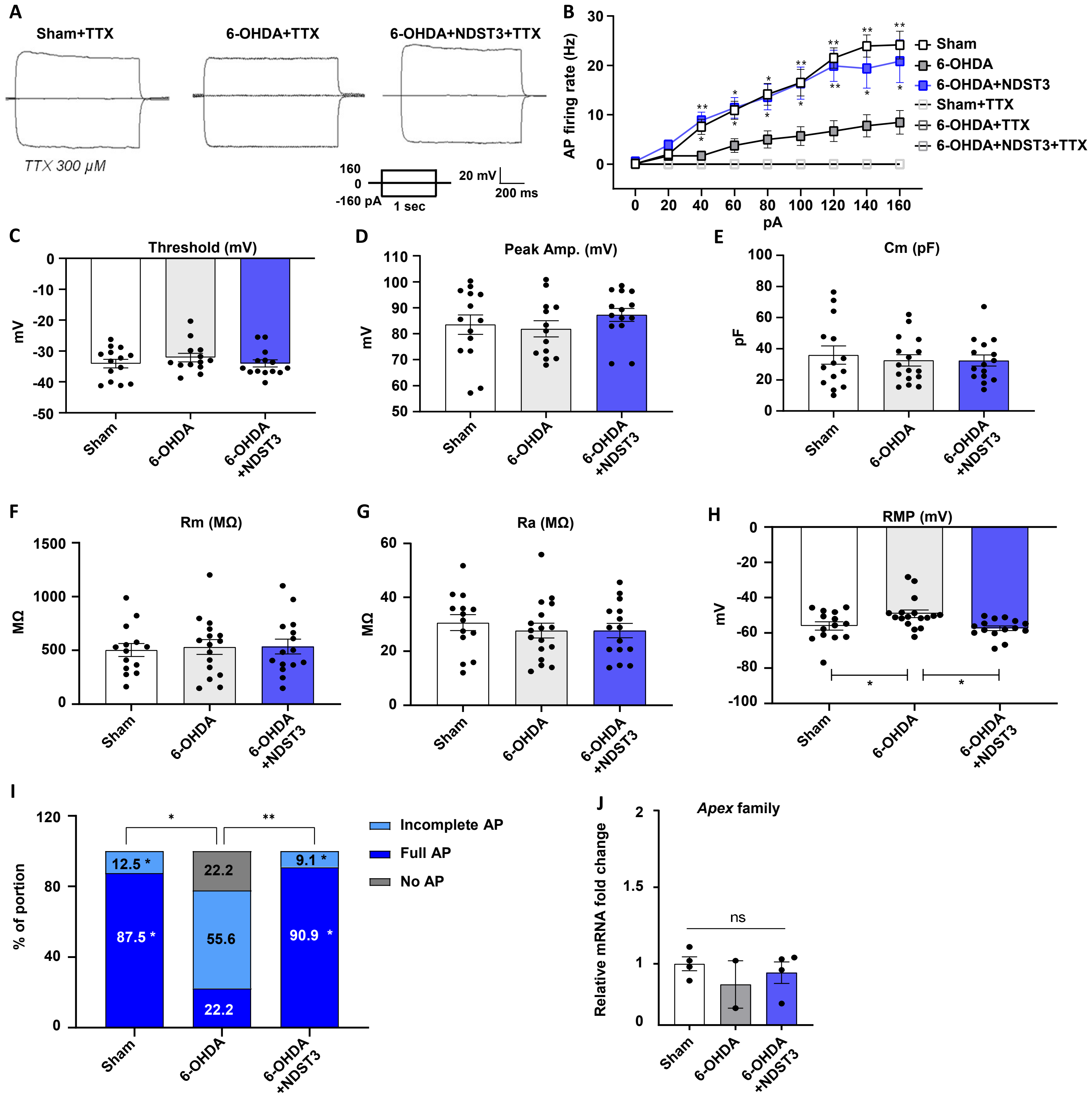

Figs S6. NDST3 treating in a Parkinson's model restored DA neuronal function. (A) Representative neuronal property confirmation via TTX (300 μM). (B) Comparison of responses (number of APs evoked by a 1 sec stimulus) for the primary DA neurons in different groups across a range of step current injections from 0 to 160 pA. Data are presented as mean ± SEM (n = 13 – 15 cells per group). Two-way ANOVA Repeated measures with Dunnett multiple comparisons test. $*p < 0.05$, $**p < 0.01$. (C) Scatter plots with bars show analyses of the Threshold in sham, 6-OHDA-induced PD model, and NDST3-treated PD model. Data are presented as mean ± SEM (n = 13 – 14 cells per group). (D) Scatter plots with bars show analyses of the Peak amplitude in sham, 6-OHDA-induced PD model, and NDST3-treated PD model. Data are presented as mean ± SEM (n = 13 – 14 cells per group). (E) Scatter plots with bars show analyses of the membrane capacitance (Cm) in sham, 6-OHDA-induced PD model, and NDST3-treated PD model. Data are presented as mean ± SEM (n = 14 – 16 cells per group). (F) Scatter plots with bars show analyses of the membrane resistance (Rm) in sham, 6-OHDA-induced PD model, and NDST3-treated PD model. Data are presented as mean ± SEM (n = 14 – 16 cells per group). (G) Scatter plots with bars show analyses of the access resistance (Ra) in sham, 6-OHDA-induced PD model, and NDST3-treated PD model. Data are presented as mean ± SEM (n = 14 – 17 cells per group). (H) Scatter plots with bars show analyses of the Resting membrane potential (RMP) in sham, 6-OHDA-induced PD model, and NDST3-treated PD model. Data are presented as mean ± SEM (n = 14 – 17 cells per group). One-way ANOVA with Tukey's multiple comparison test, $*p < 0.05$. (I) Analysis of proportion of full AP, incomplete AP and no AP in each condition (sham, 6-OHDA-induced PD model, and NDST3-treated PD model). Data are presented as relative proportion of AP ; Chi-square test., $*p < 0.05$ and $**p < 0.01$. (J) Gene expression of *Apex* family in sham, 6-OHDA-induced PD model, and NDST3-treated PD model. Data are presented as mean ± SEM (n = 3 wells per group). One-way ANOVA with Tukey's multiple comparisons test. ns = not significant.

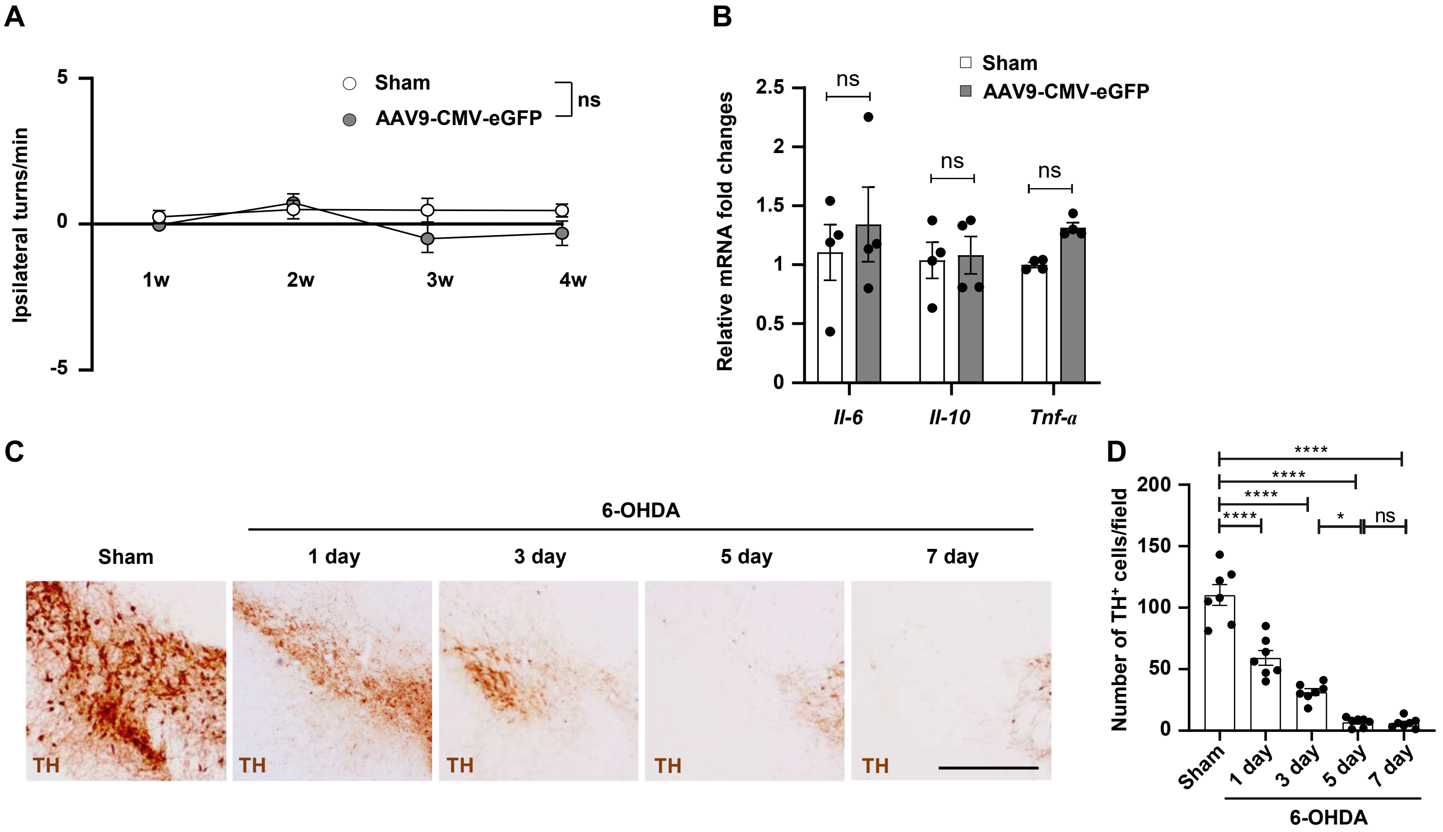

Figs S7. Confirmation of AAV9-CMV-eGFP treatment in wild-type mice and validation of toxin-derived PD model. (A) Ipsilateral turns per minute in apomorphine induced rotation test of sham and AAV9-CMV-eGFP control. Data are presented as mean ± SEM (n = 4 – 5 independent animal per group). Two-way ANOVA with Sidak's multiple comparisons test, ns = not significant. (B) Relative mRNA expression levels of inflammatory markers, *Il-6, Il-10, and Tnf-α*, following AAV9-CMV-eGFP administration. Data are presented as mean ± SEM (n = 4 independent animal per group). Two-way ANOVA with Sidak's multiple comparisons test, ns = not significant. (C) DAB-TH staining in SN, with (D) corresponding quantitative analysis. Scale bar = 500 μm. Data are presented as mean ± SEM (n = 7 independent animal per group). One-way ANOVA with Tukey's multiple comparisons test. *p < 0.05, ****p < 0.0001, and ns = not significant.

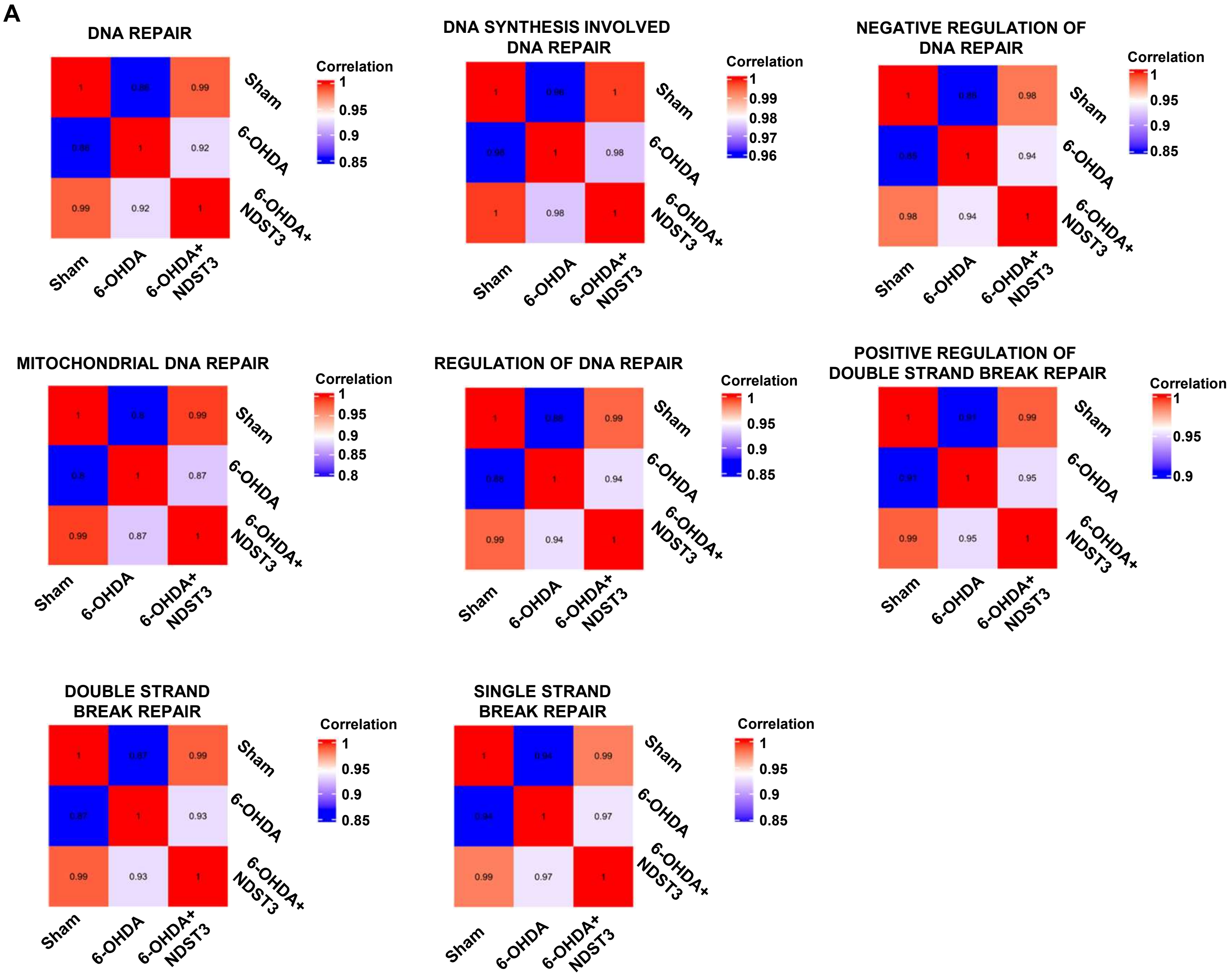

Figs S8. Hierarchical clustering and Pearson correlation. (A) Pearson correlation matrix among each group.

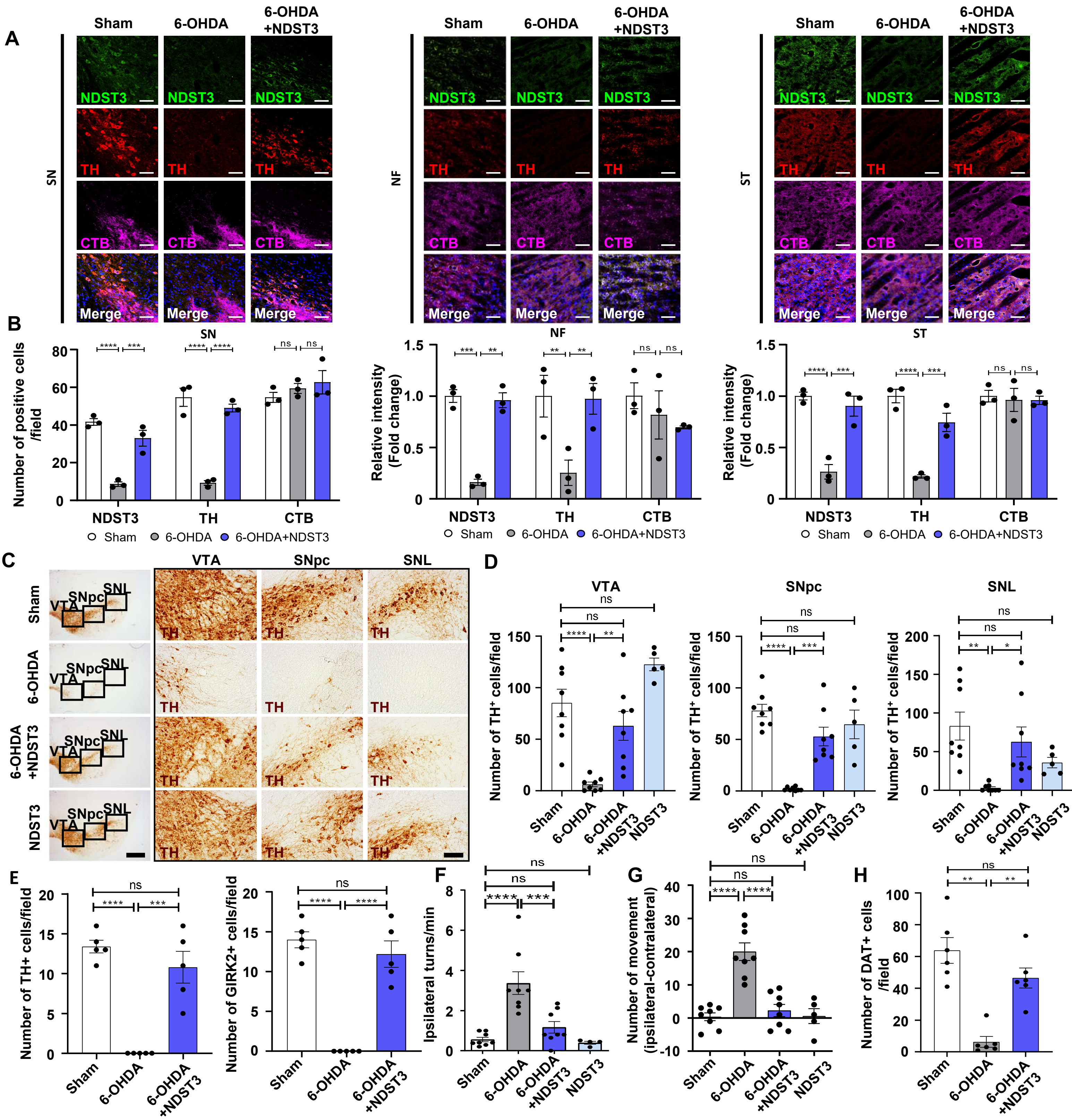

Figs S9. Efficacy of NDST3 in striatum and substantia nigra in 6-OHDA-induced PD model and NDST3 treatment. (A) Representative immunofluorescence images of NDST3, TH and CTB in the substantia nigra (SN), nigrostriatal fiber (NF), and striatum (ST) region. Scale bar = 50µm. (B) Quantification of the relative intensity and number of positive cells as in Fig S9A. Data are presented as mean ± SEM (n = 3 independent animal per group). One-way ANOVA with Tukey's multiple comparisons test. **p < 0.01, ***p < 0.001, ****p < 0.0001 and ns = not significant. (C-D) DAB-TH staining in ventral tegmental area (VTA), substantia nigra pars compacta (SNpc), and substantia nigra pars lateralis (SNL), with corresponding quantitative analysis. Data are presented as mean ± SEM (n = 5 – 8 independent animal per group). One-way ANOVA with Tukey's multiple comparisons test. *p < 0.05, **p < 0.01, ***p < 0.001, ****p < 0.0001 and ns = not significant. Scale bar = 500µm, 100 µm (Magnification image). (E) Quantification of the number of TH (left panel) and GIRK2 (right panel) as depicted in Fig. 2D . Data are presented as mean ± SEM (n = 5 independent animal per group). One-way ANOVA with Tukey's multiple comparisons test. ***p < 0.001, ****p < 0.0001 and ns = not significant. (F) Ipsilateral turns per minute in apomorphine induced rotation test of sham, 6-OHDA-induced PD model, and NDST3-treated PD model. Data are presented as mean ± SEM (n = 4 – 8 independent animal per group). One-way ANOVA with Tukey's multiple comparisons test. ***p < 0.001, ****p < 0.0001 and ns = not significant. (G) Number of body bending movements in tail suspension test. Data are presented as mean ± SEM (n = 5 – 8 independent animal per group). One-way ANOVA with Tukey's multiple comparisons test. ****p < 0.0001 and ns = not significant. (H) Quantification of number of DAT+ cells as shown in Fig. 2F. Data are presented as mean ± SEM (n = 6 independent animal per group). One-way ANOVA with Tukey's multiple comparisons test. **p < 0.01 and ns = not significant.

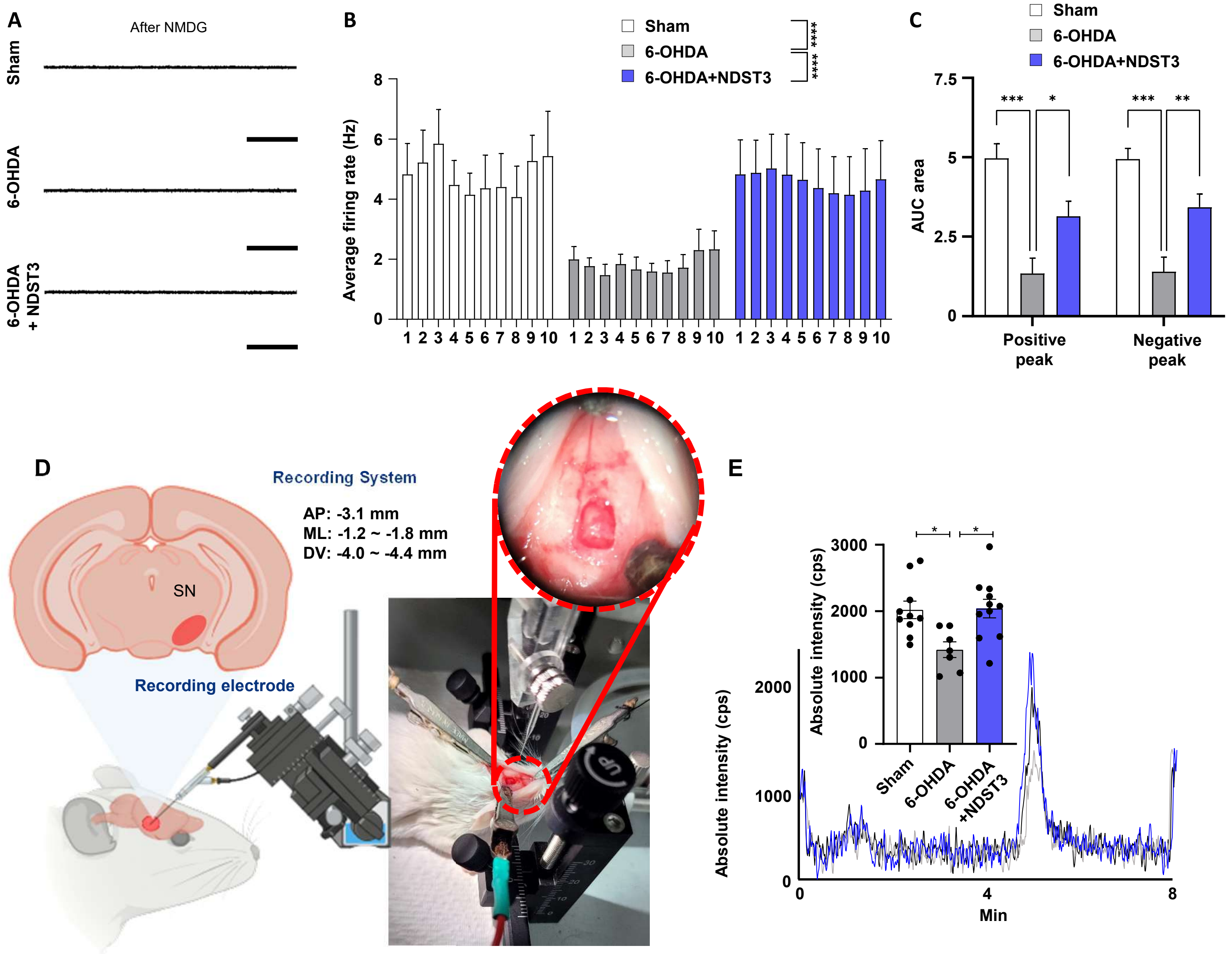

Figs S10. Electrophysiological analysis of dopaminergic neurons in 6-OHDA-induced PD model and NDST3 treatment. (A) Representative traces of spontaneous firing currents recorded from DA neurons of the SNpc in brain slices from each condition. The firing currents confirmed via NMDG solution. (B) Mean firing rate for 10 minutes of DA neurons recorded from SN in NDST3-treated PD models. Data are presented as mean ± SEM (n = 4 – 6 independent animal per group). Two-way ANOVA with Dunnett's multiple comparisons test. ****p < 0.0001. (C) Restoration of the reduced waveform area under the curve (AUC) in the PD mouse model by NDST3 administration. Data are presented as mean ± SEM (n = 4 – 6 independent animal per group). One-way ANOVA with Tukey's multiple comparisons test. *p < 0.05, **p < 0.01, ***p < 0.001. (D) Schematic diagram of *in vivo* single-unit recording. Schematic illustration created with Biorender.com. (E) Dopamine detection in the striatum post-6-OHDA treatment and NDST3 administration. Quantification of absolute intensity of dopamine. Data are presented as mean ± SEM (n = 7 – 11 independent animal per group). One-way ANOVA with Tukey's multiple comparisons test. *p < 0.05.

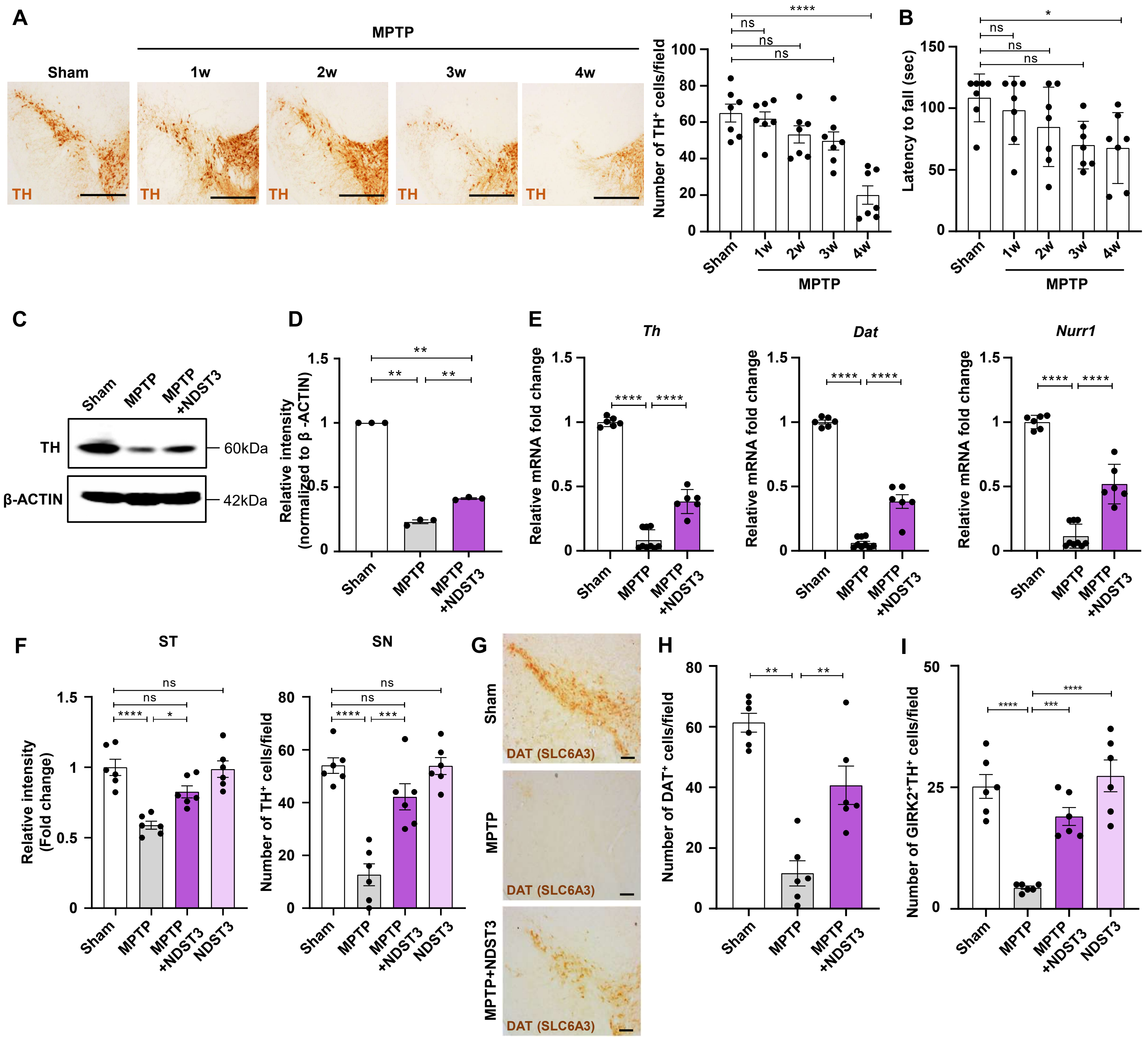

Figs S11. Efficacy of NDST3 in MPTP-induced PD model. (A) DAB-TH staining in SN with corresponding quantitative analysis. Data are presented as mean ± SEM (n =7 independent animal per group). One-way ANOVA with Tukey's multiple comparisons test. ****$p < 0.0001$. Scale bar = 500 µm. (B) Fall latency in the wire-hanging test. The data are presented as mean ± SEM (n = 7 independent animal per group). One-way ANOVA with Tukey's multiple comparisons test. *$p < 0.05$. (C) Immunoblot analysis showing TH expression in lysates from sham, MPTP-induced PD model, and NDST3-treated PD model. Identification of specific TH (60 kDa) protein, with β-ACTIN serving as a loading control. (D) Intensity quantification as shown in Fig S11C. Data are presented as mean ± SEM (n = 3 independent animal per group). One-way ANOVA with Tukey's multiple comparisons test. **$p < 0.01$. (E) mRNA expression analysis of DA neuronal markers (*Th*, *Dat*, and *Nurr1*). Data are presented as mean ± SEM (n = 6 – 9 independent animal per group). One-way ANOVA with Tukey's multiple comparisons test. ****$p < 0.0001$. (F) Quantification of relative intensity and number of TH-positive cells as shown in Fig. 3G. Data are presented as mean ± SEM (n = 6 independent animal per group). One-way ANOVA with Tukey's multiple comparisons test. *$p < 0.05$, ***$p < 0.001$, ****$p < 0.0001$ and ns = not significant. (G) Representative image of DAB-DAT staining in SN, with (H) the corresponding quantitative analysis shown in fig S11E. Data are presented as mean ± SEM (n = 6 independent animal per group). One-way ANOVA with Tukey's multiple comparisons test. **$p < 0.01$. Scale bar = 100 µm. (I) Quantification of GIRK2 and TH-positive cells as shown in Fig. 3H. Data are presented as mean ± SEM (n = 6 independent animal per group). One-way ANOVA with Tukey's multiple comparisons test. ***$p < 0.001$ and ****$p < 0.0001$.

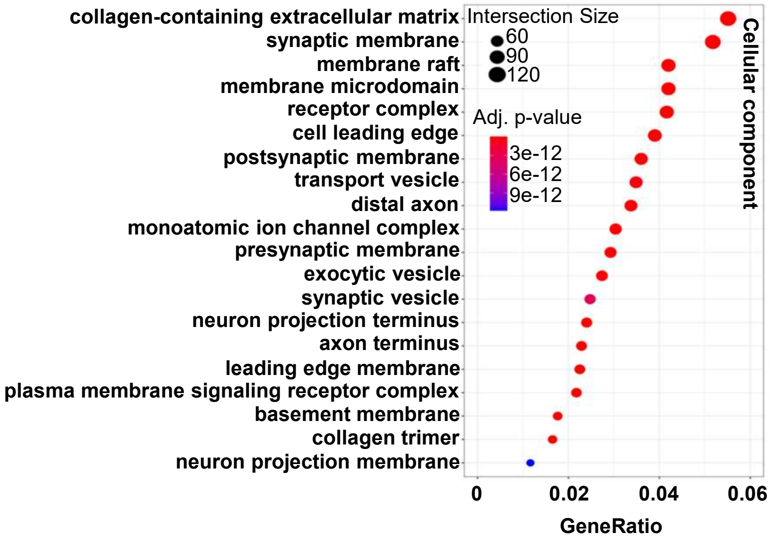

Figs S12. RNA-seq analysis of NDST3-treated PD model. Dot plot of top 20 GO cellular component terms from GO enrichment analysis for 6-OHDA+NDST3 vs. 6-OHDA.

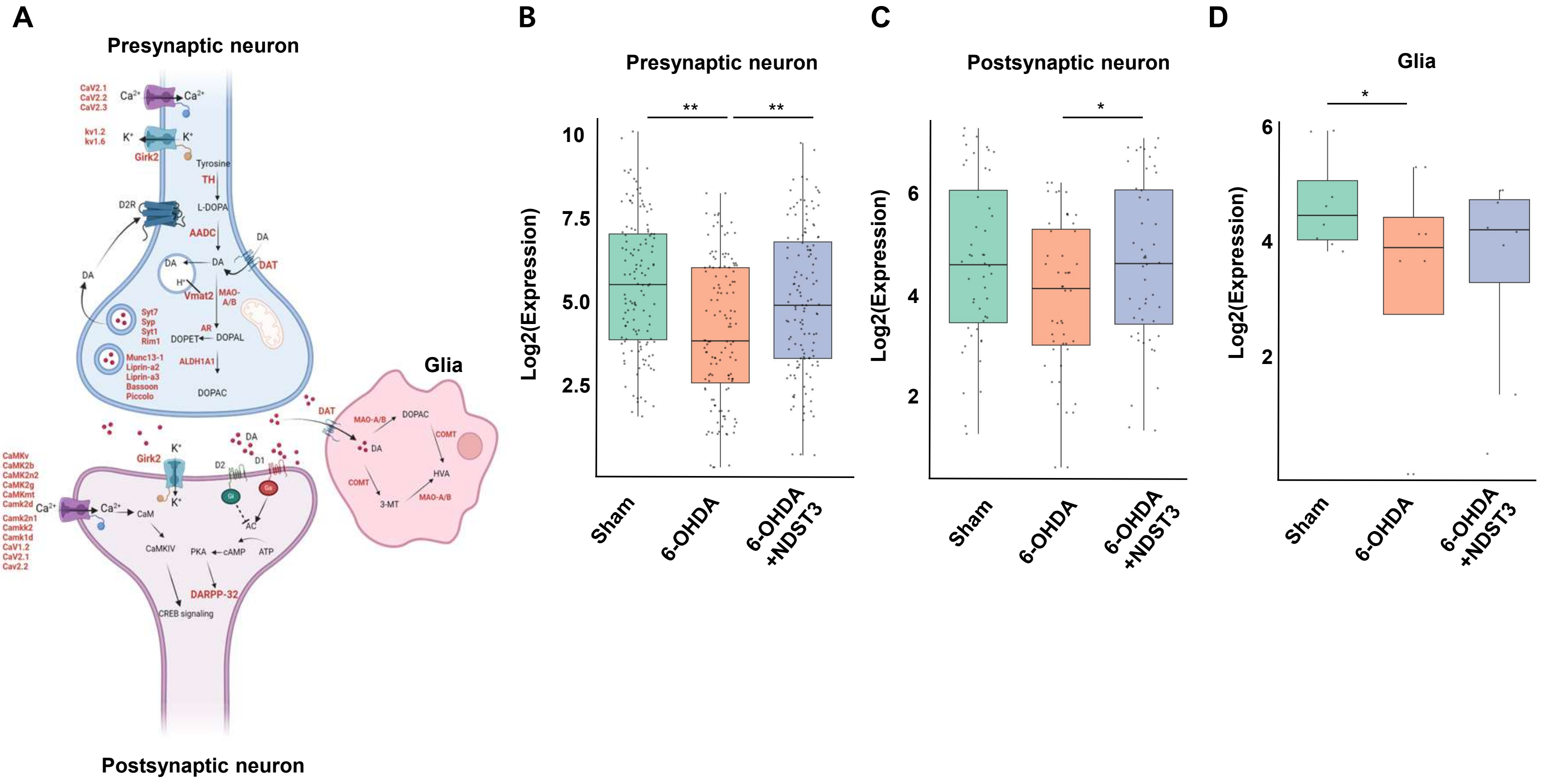

Figs S13. Dopaminergic circuitry. (A) Schematic representation of dopaminergic circuitry. GSVA analysis of (B) dopaminergic presynaptic terminal, (C) postsynaptic terminal, and (D) glia, indicating statistical significance as *p < 0.05, and **p < 0.01. Schematic illustration created with Biorender.com.

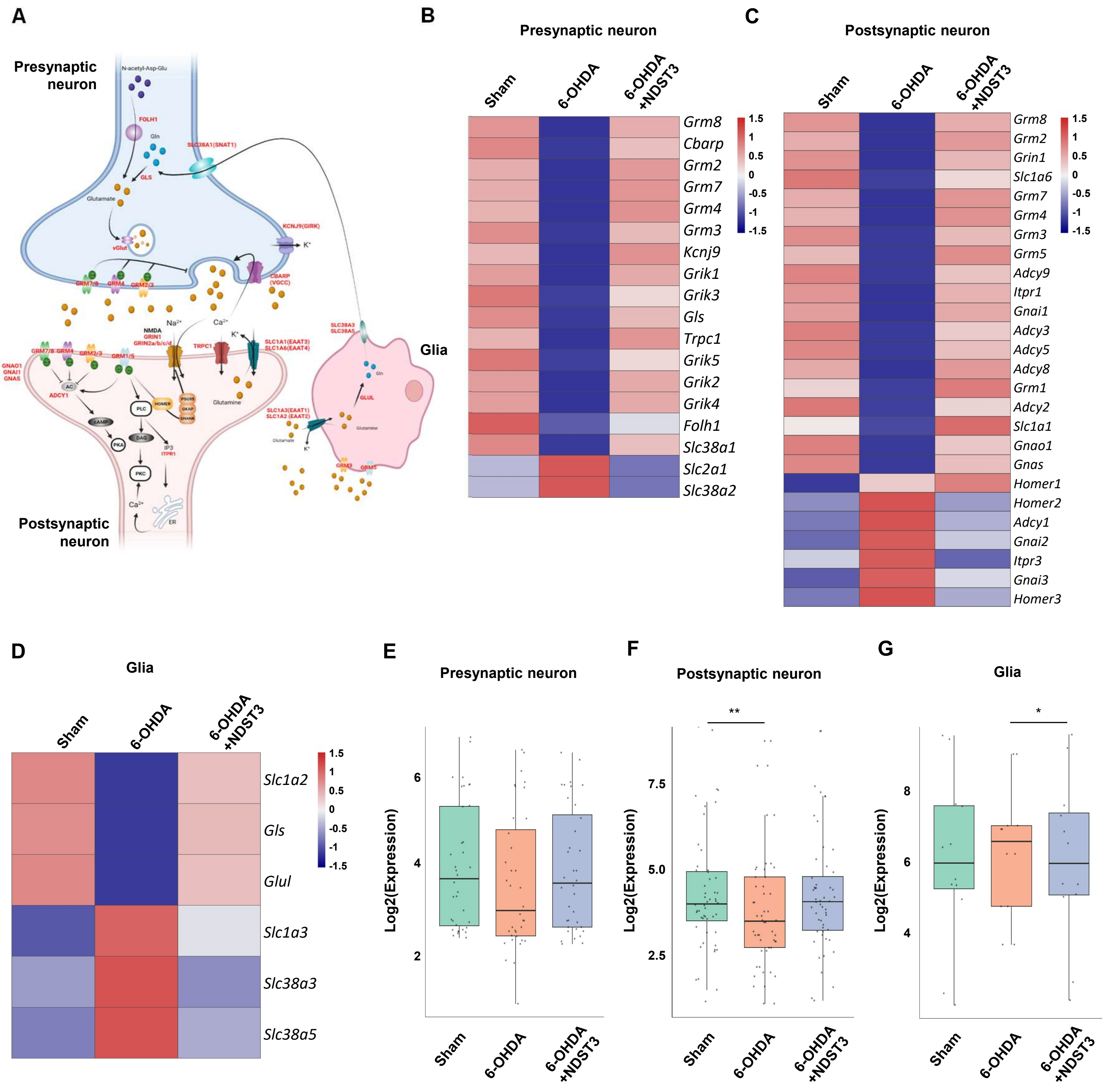

Figs S14. Glutamatergic circuitry. (A) Schematic representation of glutamatergic circuitry. Heatmap representing gene expression patterns of (B)glutamatergic presynaptic terminals, (C) postsynaptic terminal and (D) glia in each group (sham, 6-OHDA, and 6-OHDA + NDST3). GSVA analysis of (E) glutamatergic presynaptic terminal, (F) postsynaptic terminal, and (G) glia, indicating statistical significance as *p < 0.05, and **p < 0.01. Schematic illustration created with Biorender.com.

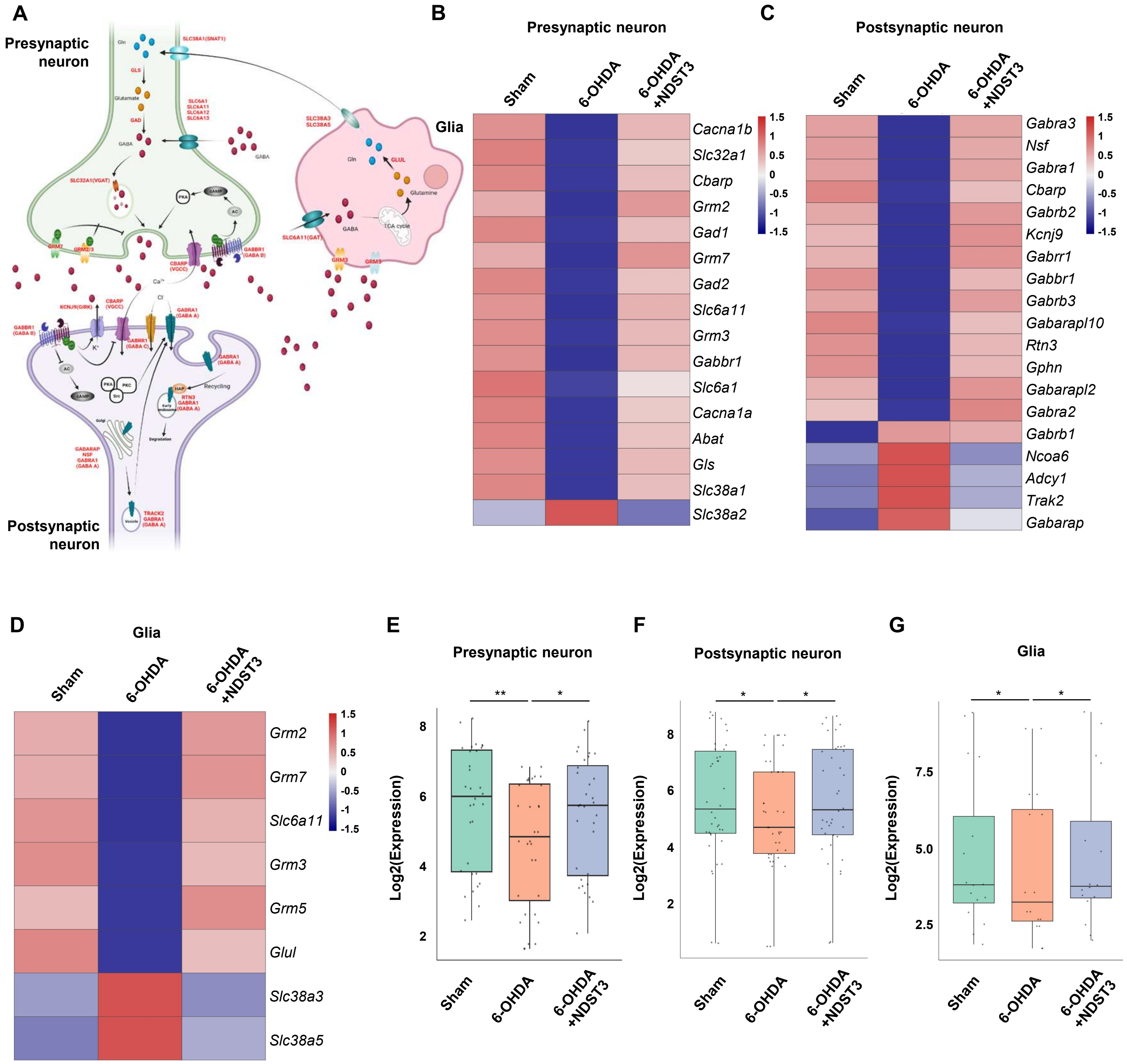

Figs S15. GABAergic circuitry. (A) Schematic representation of GABAergic circuitry. Heatmap representing gene expression patterns of (B) GABAergic presynaptic terminals, (C) postsynaptic terminal and (D) glia in each group (sham, 6-OHDA, and 6-OHDA + NDST3). GSVA analysis of (E) GABAergic presynaptic terminal, (F) postsynaptic terminal, and (G) glia, indicating statistical significance as *p < 0.05, and **p < 0.01. Schematic illustration created with Biorender.com.

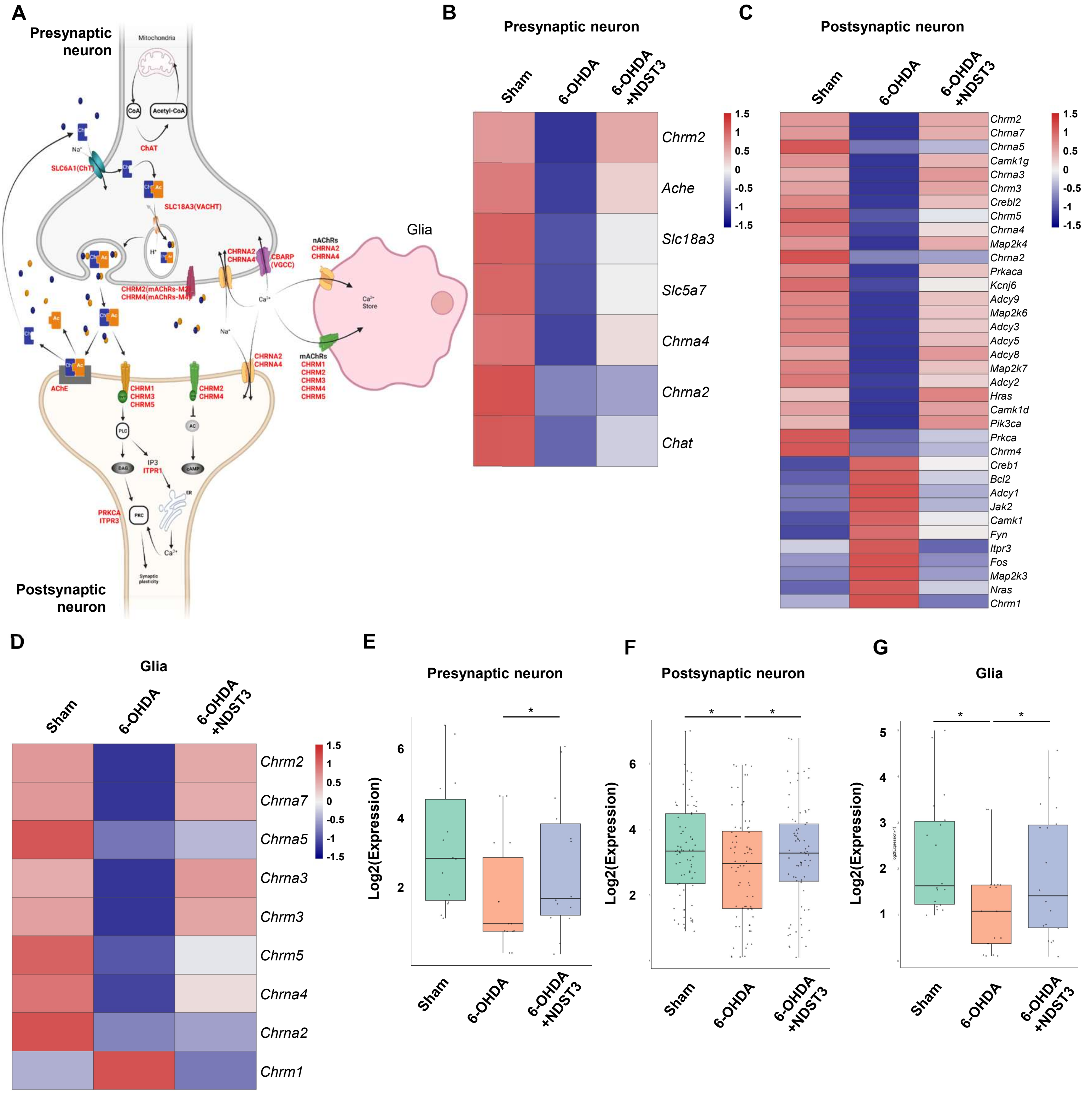

Figs S16. Cholinergic circuitry. (A) Schematic representation of cholinergic circuitry. Heatmap representing gene expression patterns of (B) cholinergic presynaptic terminals, (C) postsynaptic terminal and (D) glia in each group (sham, 6-OHDA, and 6-OHDA + NDST3). GSVA analysis of (E) cholinergic presynaptic terminal, (F) postsynaptic terminal, and (G) glia, indicating statistical significance as $*p < 0.05$. Schematic illustration created with Biorender.com.

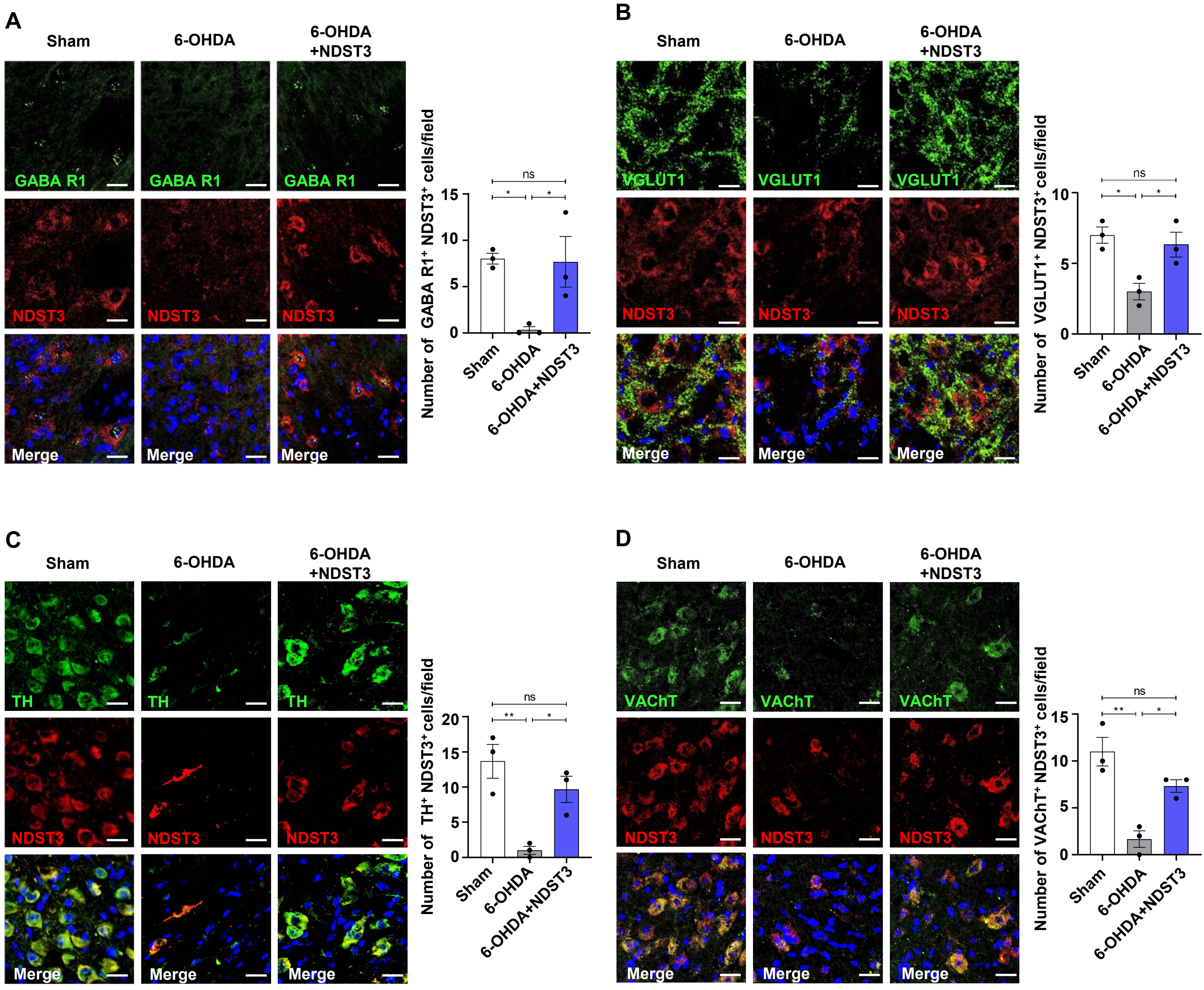

Figs S17. Confirmation of NDST3 expression in specific neurons. (A) Representative immunofluorescence images and quantification of GABA R1 and NDST3 in SN. Scale bar = 20 μm. Data are presented as mean ± SEM (n = 3 independent animal per group). One-way ANOVA with Tukey's multiple comparisons test. *p < 0.05 and ns = not significant. (B) Representative immunofluorescence images and quantification of VGLUT1 and NDST3 in SN. Scale bar = 20 μm. Data are presented as mean ± SEM (n = 3 independent animal per group). One-way ANOVA with Tukey's multiple comparisons test. *p < 0.05 and ns = not significant. (C) Representative immunofluorescence images and quantification of TH and NDST3 in SN. Scale bar = 20 μm. Data are presented as mean ± SEM (n = 3 independent animal per group). One-way ANOVA with Tukey's multiple comparisons test. *p < 0.05, **p < 0.01 and ns = not significant. (D) Representative immunofluorescence images and quantification of VAChT and NDST3 in SN. Scale bar = 20 μm. Data are presented as mean ± SEM (n = 3 independent animal per group). One-way ANOVA with Tukey's multiple comparisons test. *p < 0.05, **p < 0.01 and ns = not significant.

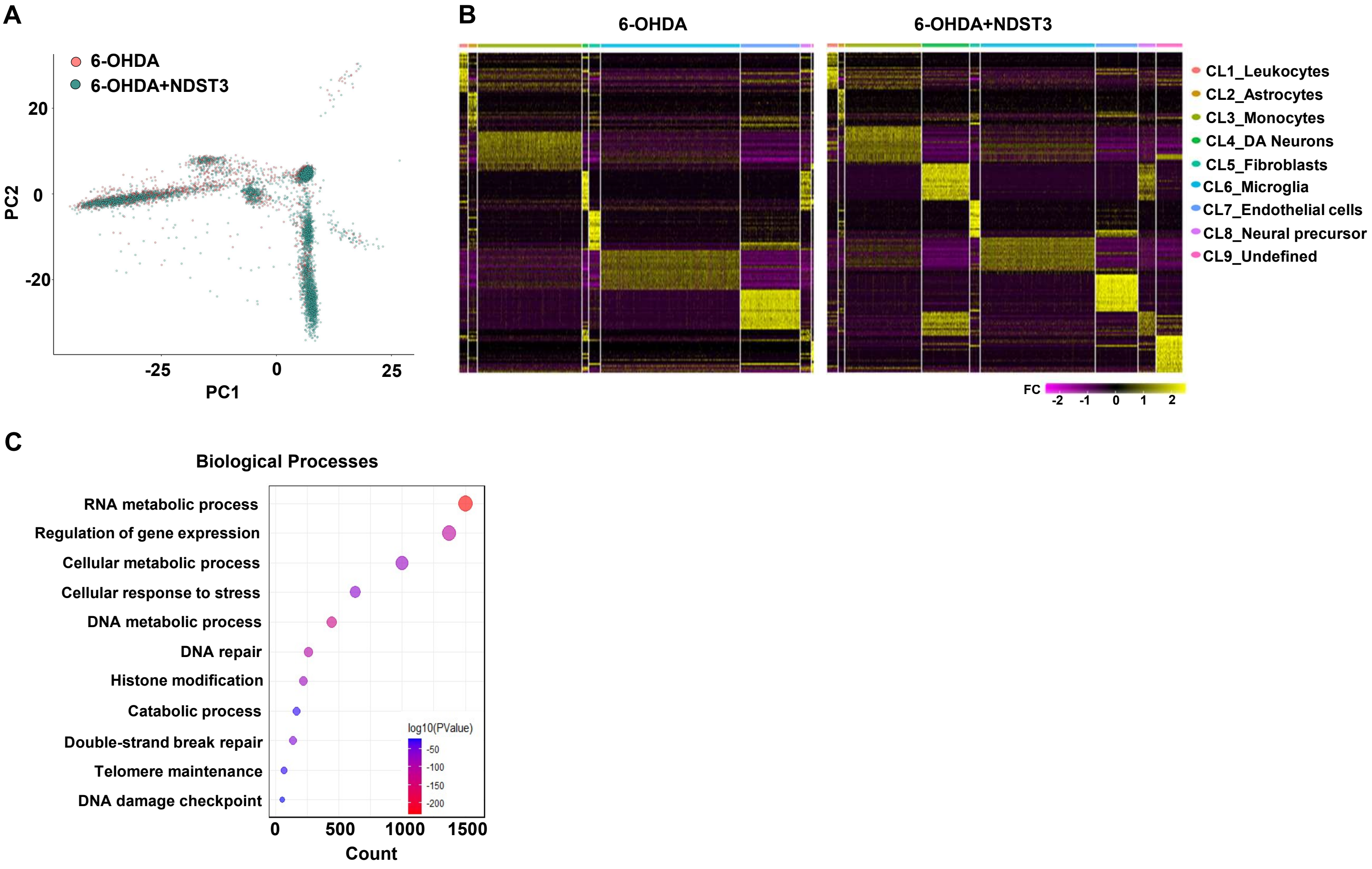

Figs S18. scRNA-seq analysis of NDST3-treated PD model. (A) PCA analysis of scRNA-seq data in 6-OHDA-induced PD model (pink) and NDST3-treated PD model (cyan). (B) Heatmap analysis of each cluster in 6-OHDA-induced PD model (left panel) and NDST3-treated PD model (right panel). (C) Dot plot displaying top 11 GO biological process terms from GO enrichment analysis for NDST3-treated PD model versus 6-OHDA-induced PD model.

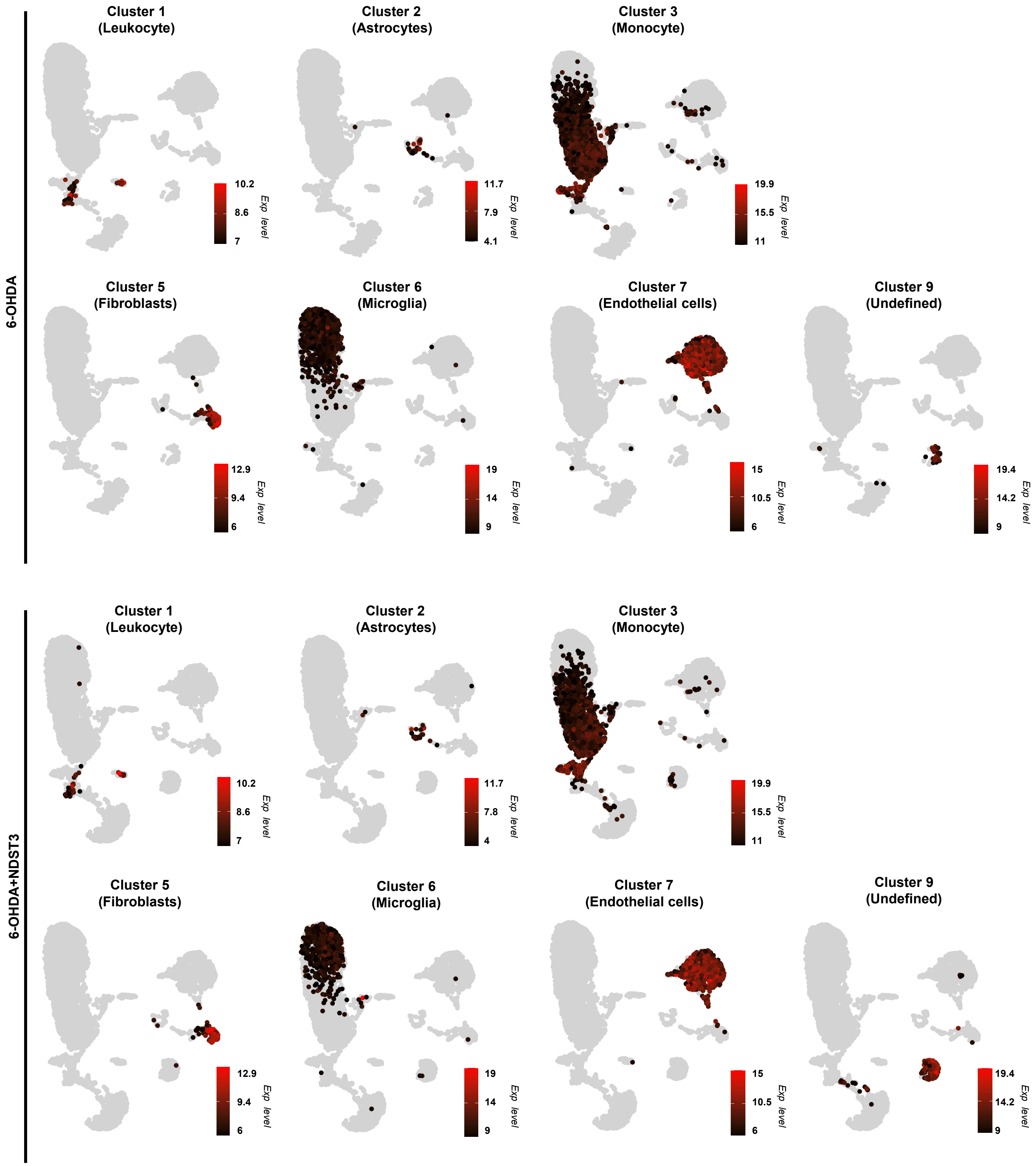

Figs S19. Feature plot of each cluster for gene expression.

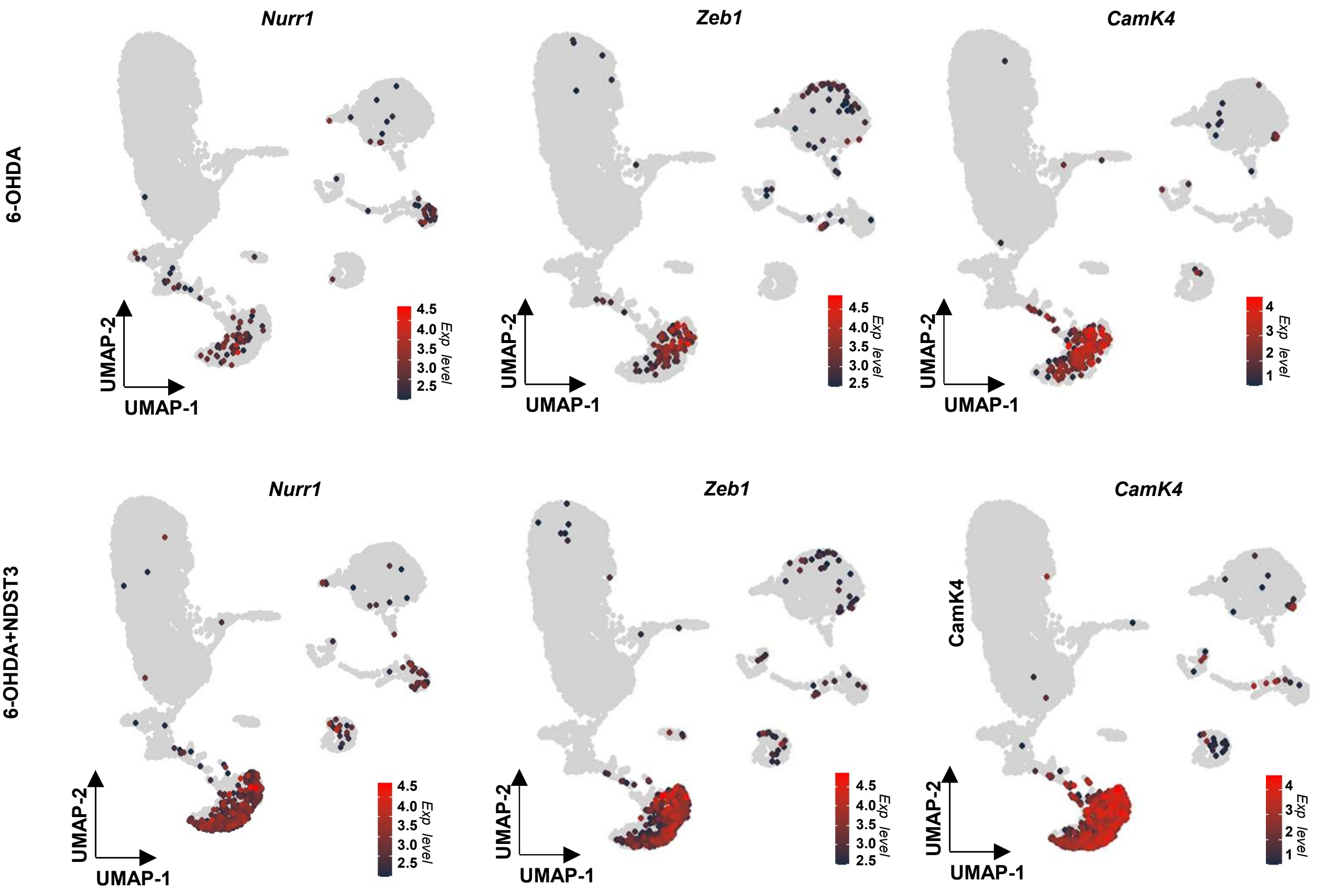

Figs S20. Single cell profiling in PD model. Feature plot analysis of *Nurr1*(Left panel), *Zeb1* (middle panel), and *CamK4* (Right panel) expression in 6-OHDA-induced PD model and NDST3-treated PD model.

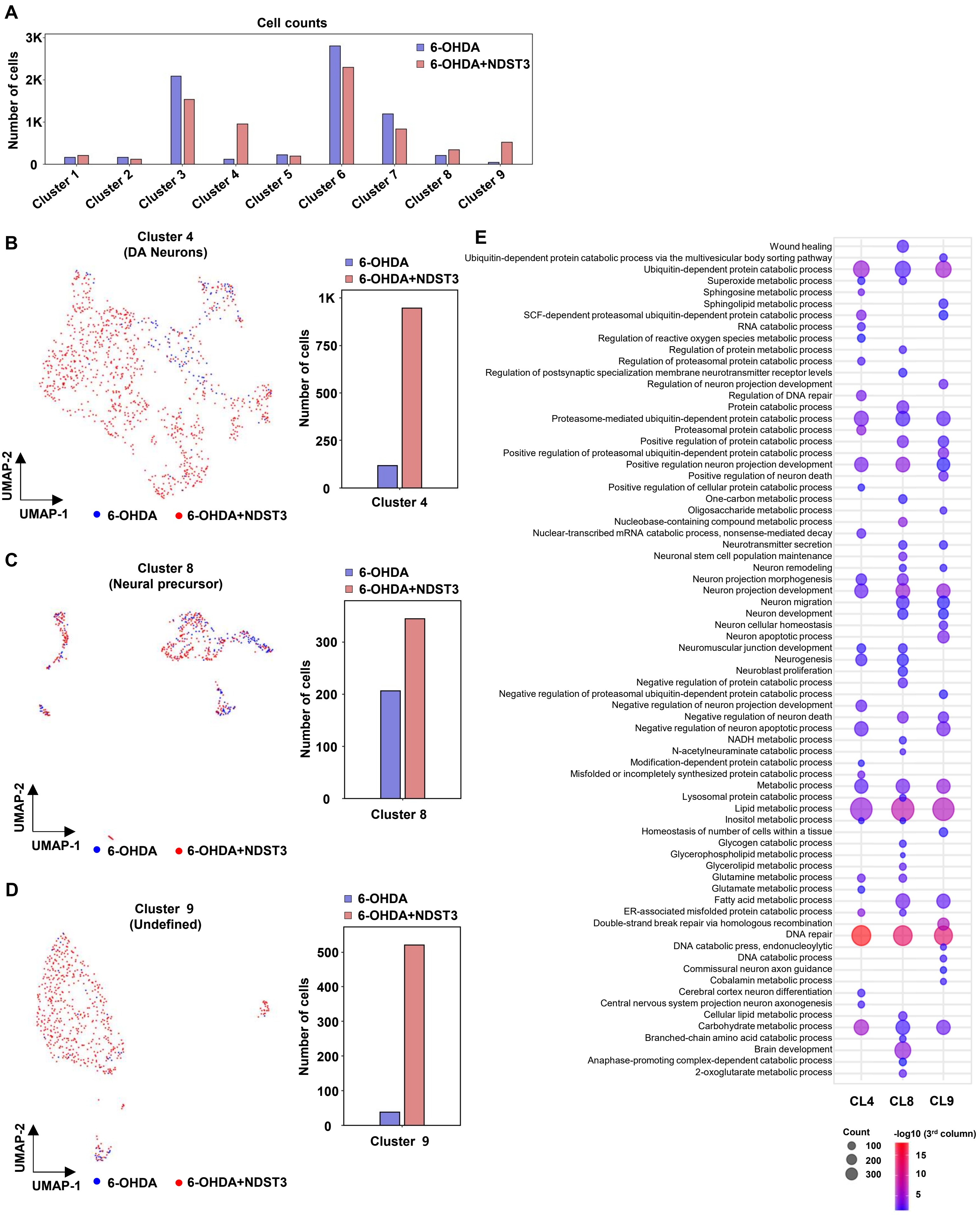

Figs S21. scRNA-seq analysis of clusters 4, 8, and 9. (A) Number of cell per clusters. (B) Uniform manifold approximation and projection (UMAP) visualization and quantification of cluster 4 cells in 6-OHDA-induced PD model and NDST3-treated PD model. (C) UMAP visualization and quantification of cluster 8 representation in 6-OHDA-induced PD model and NDST3-treated PD model. (D) UMAP visualization and quantification of cluster 9 representation in 6-OHDA-induced PD model and NDST3-treated PD model. (E) Gprofiler analysis highlighting biological process terms from GO enrichment in cluster 4, 8, and 9.

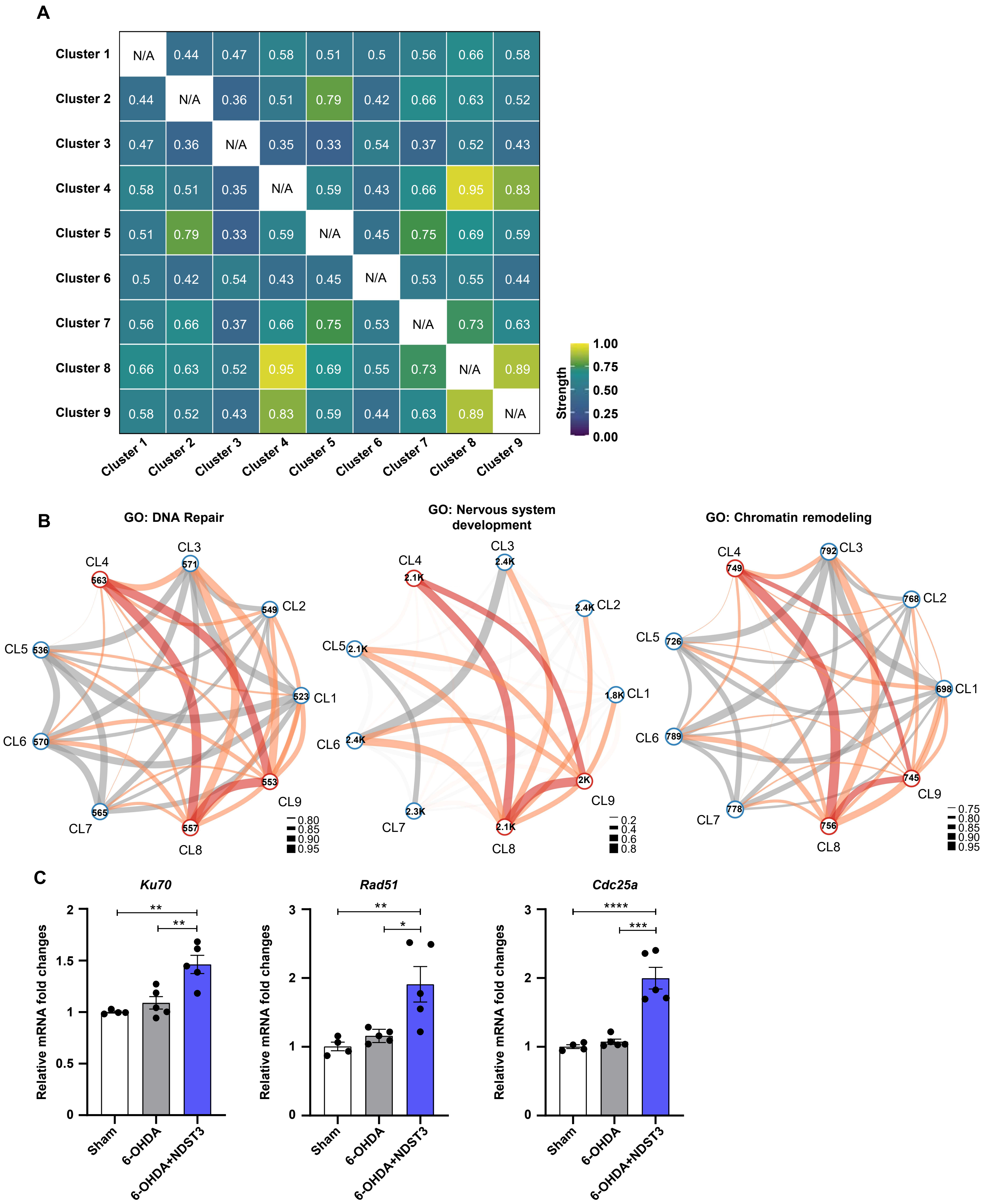

Figs S22. Interaction network within each clusters. (A) Cluster to cluster node strength plot. (B) STRING interaction network analysis of each cluster. Dot represents number of genes. (C) Gene expression level of DNA damage repair marker (*Ku70, Rad 51 and Cdc25a*) in sham, 6-OHDA-induced PD model, and NDST3-treated PD model. Data are presented as mean ± SEM (n = 4 – 5 independent animal per group). One-way ANOVA with Tukey's multiple comparison test. *p < 0.05, **p < 0.01, ***p < 0.001, and ****p < 0.0001.

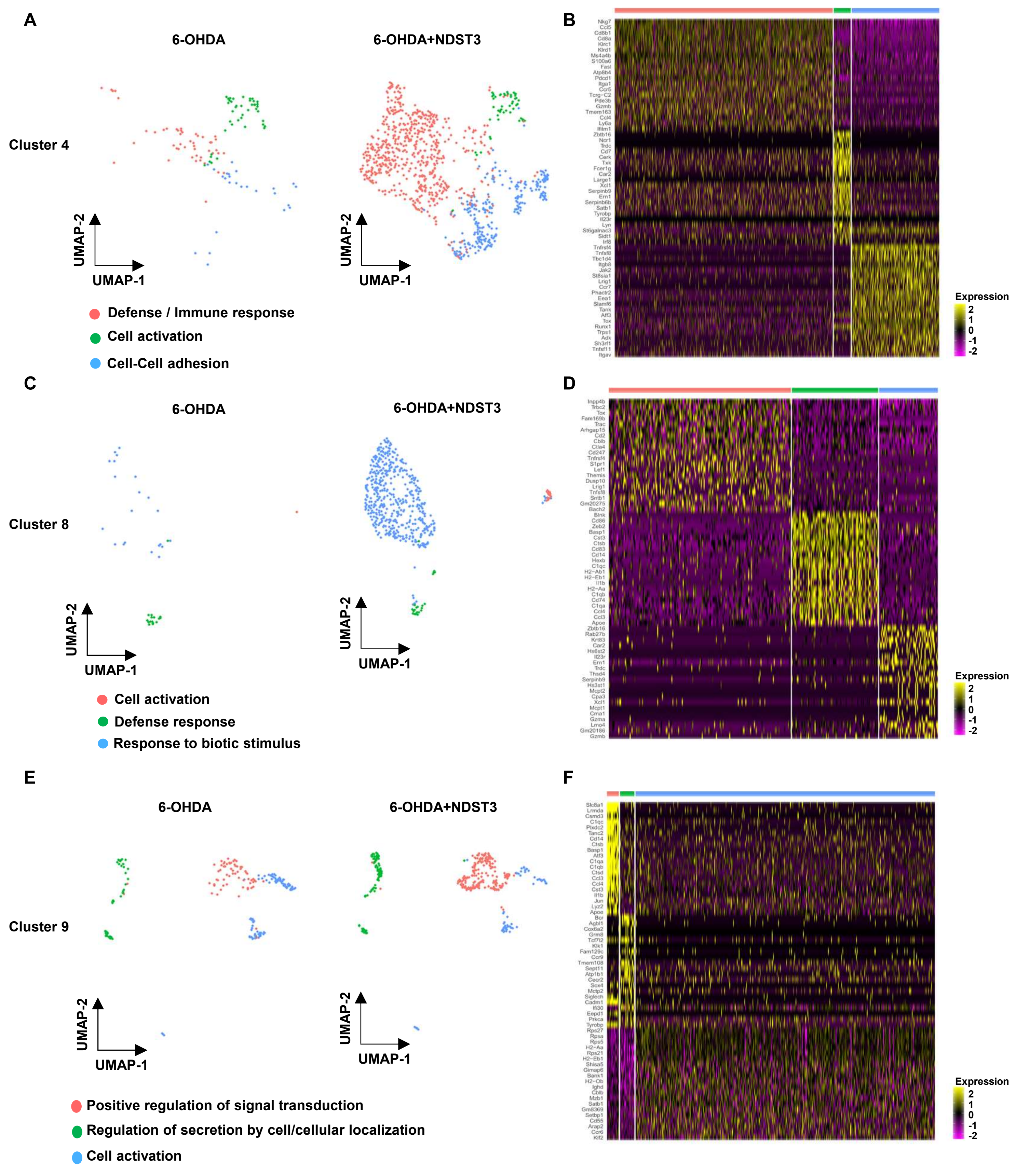

Figs S23. UMAP analysis of cluster 4, 8, and 9. (A) UMAP analysis of cluster 4 in 6-OHDA-induced PD model and NDST3-treated PD model. 3 divisions (Defense / immune response, cell activation, and cell-cell adhesion) are categorized both in 6-OHDA-induced PD model and NDST3-treated PD model. (B) Heatmap analysis of cluster 4 in NDST3-treated PD model for defense / immune response, cell activation, and cell-cell adhesion processes. (C) UMAP analysis of cluster 8 in 6-OHDA-induced PD model and NDST3-treated PD model. 3 divisions (Cell activation, defense response, and response to biotic stimulus) are categorized both in 6-OHDA-induced PD model and NDST3-treated PD model. (D) Heatmap analysis of cluster 8 in NDST3-treated PD model for cell activation, defense response, and response to biotic stimulus processes. (E) UMAP analysis of cluster 9 in 6-OHDA-induced PD model and NDST3-treated PD model. 3 divisions (Positive regulation of signal transduction, regulation of secretion by cell/cellular localization, and cell activation) are categorized both in 6-OHDA-induced PD model and NDST3-treated PD model. (F) Heatmap analysis of cluster 9 in NDST3-treated PD model for positive regulation of signal transduction, regulation of secretion by cell/cellular localization, and cell activation processes.

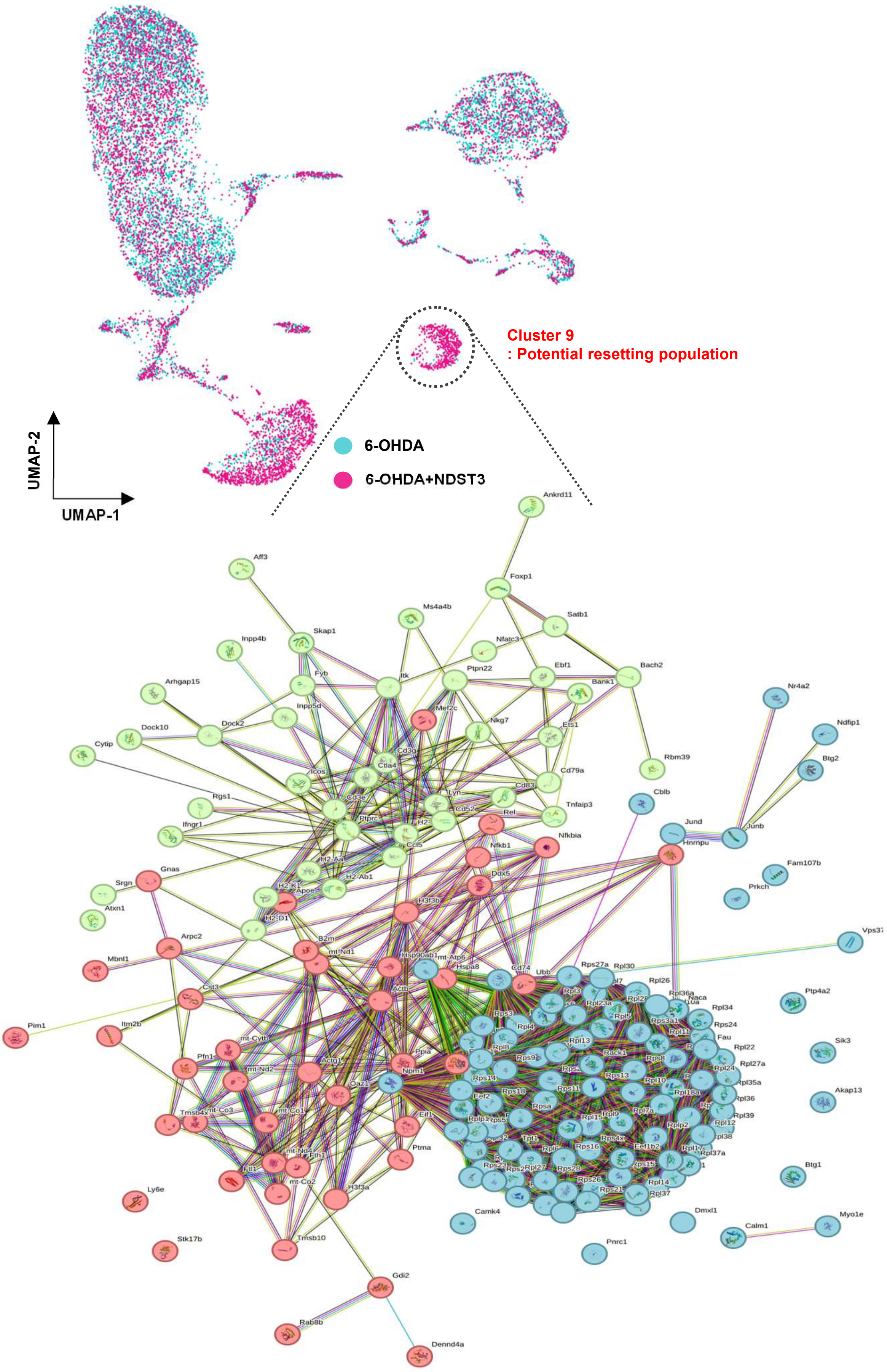

Figs S24. UMAP analysis of cluster 9, which can be potential resetting population and its STRING network analysis.

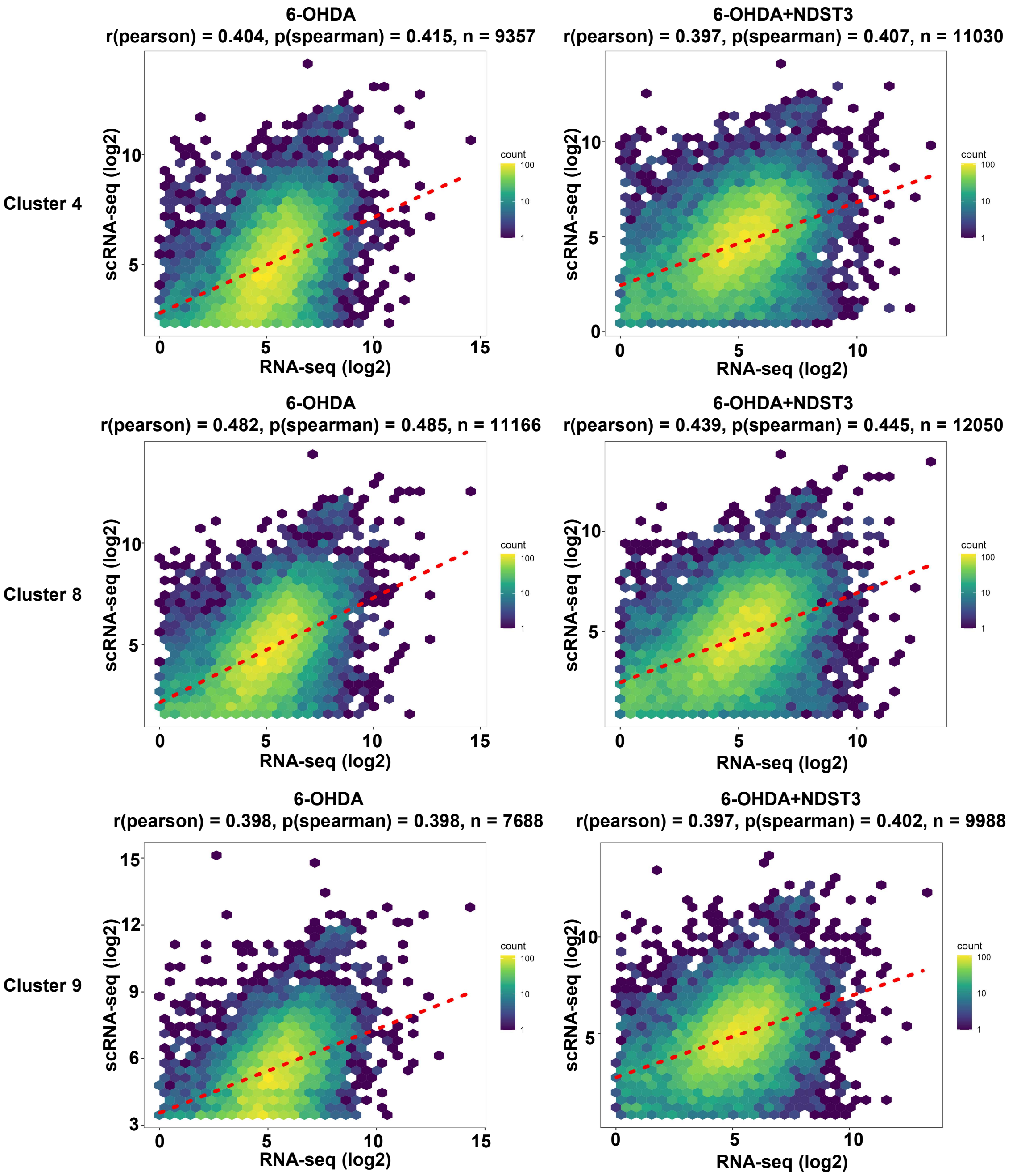

Figs S25. Hexbin plot comparing expression in RNA-seq and scRNA-seq. The red line indicates the linear regression.

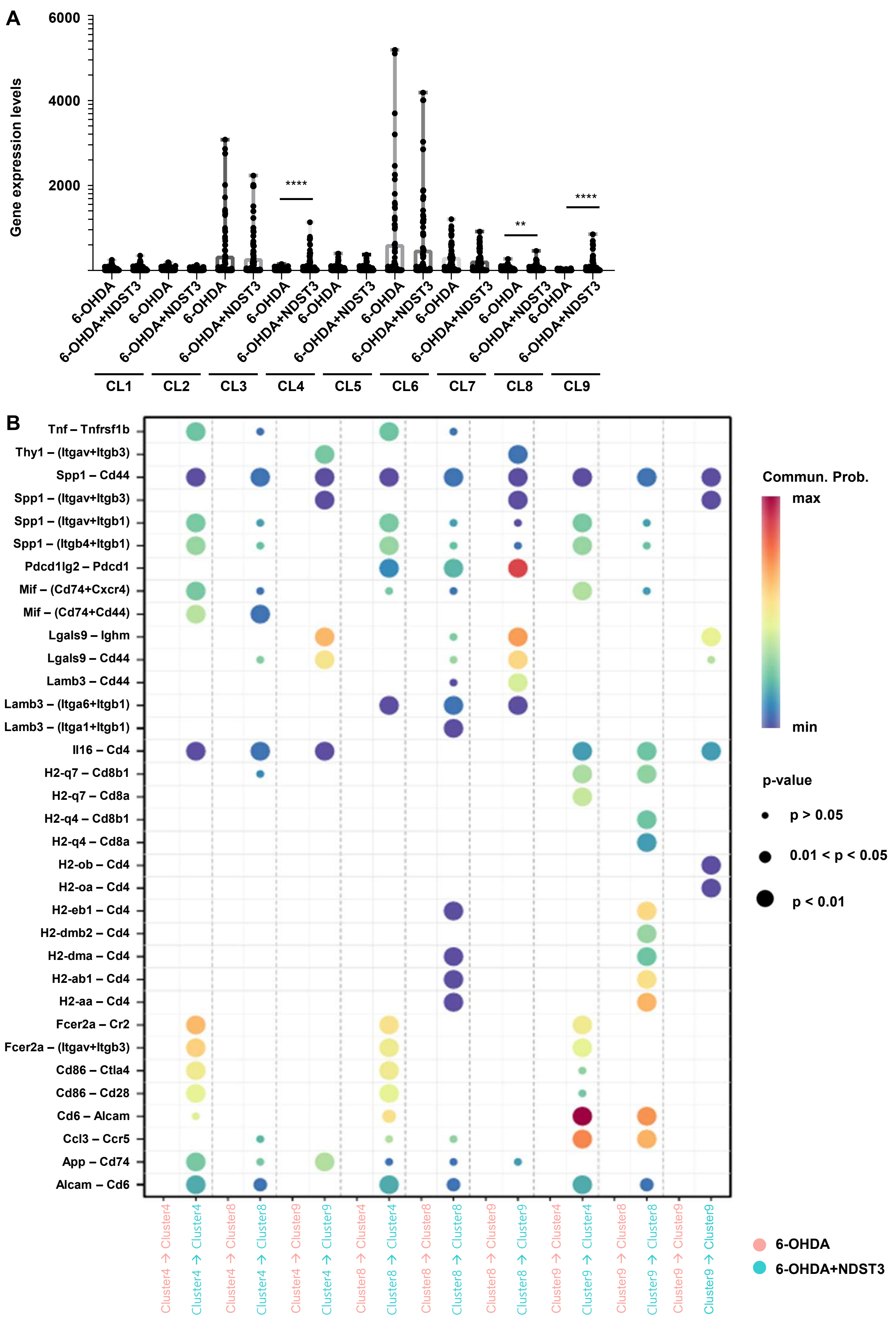

Figs S26. Molecular dynamics of dopaminergic synapses and ligand-receptor interactions in PD model. (A) Expression levels of pre and post dopaminergic synaptic related genes in each of clusters defined by scRNA-seq, indicating statistical significance as $^{**}p < 0.01$, and $^{****}p < 0.0001$. (B) Comparison of the significant ligand-receptor pairs between 6-OHDA-induced PD model and NDST3 treated PD model, which contribute to the signaling.

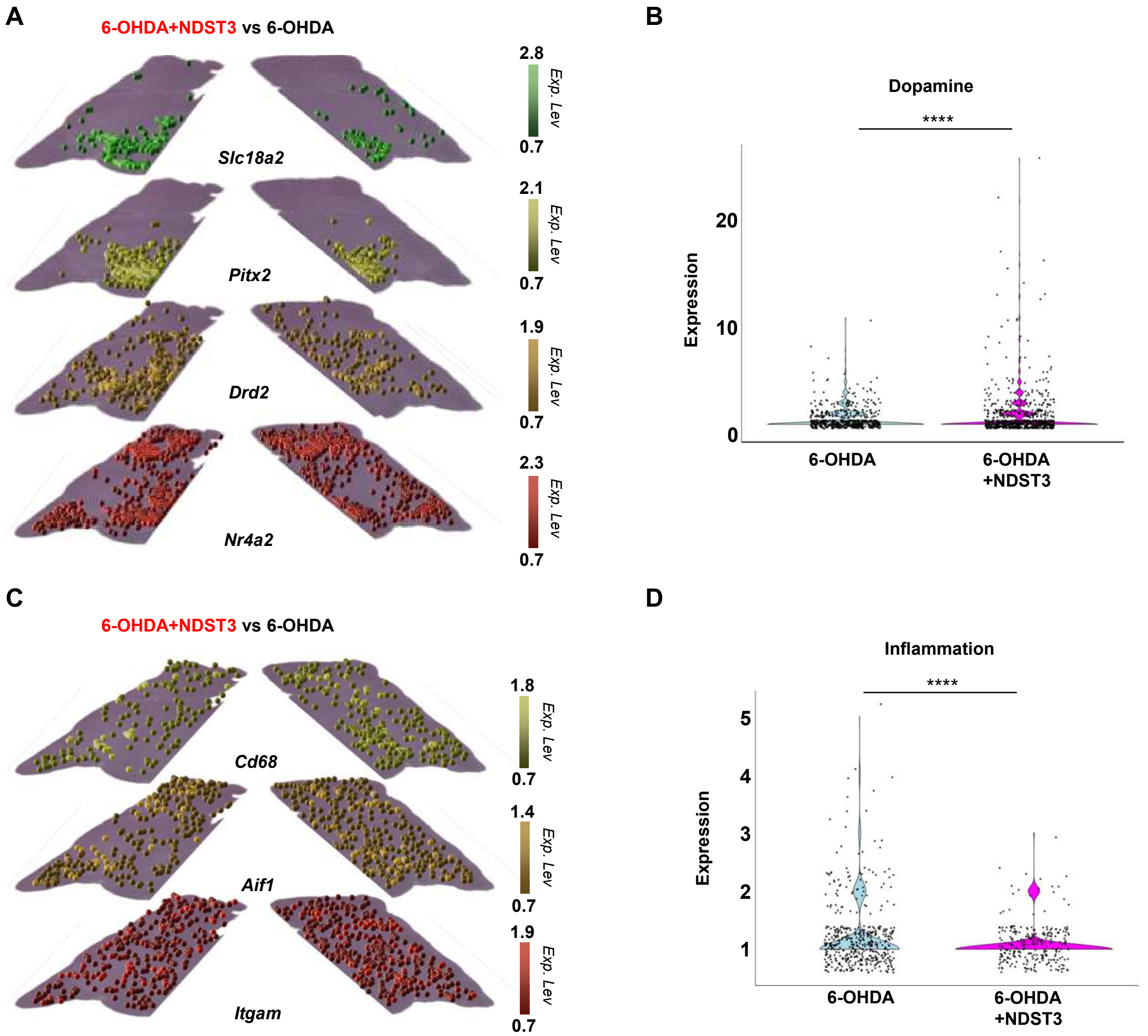

Figs S27. Spatial transcriptomics in PD model using Visium. (A) Spatial RNA transcription analysis which shows localization of cell resetting and dopaminergic lineage markers expression. (B) Quantification of expression levels of dopaminergic lineage markers in 6-OHDA-induced PD model and NDST3-treated PD model, indicating statistical significance as ****$p < 0.0001$. (C) Spatial RNA transcription analysis which shows localization of inflammation markers expression. (D) Quantification of expression levels of inflammation markers in 6-OHDA-induced PD model and NDST3-treated PD model, indicating statistical significance as ****$p < 0.0001$.

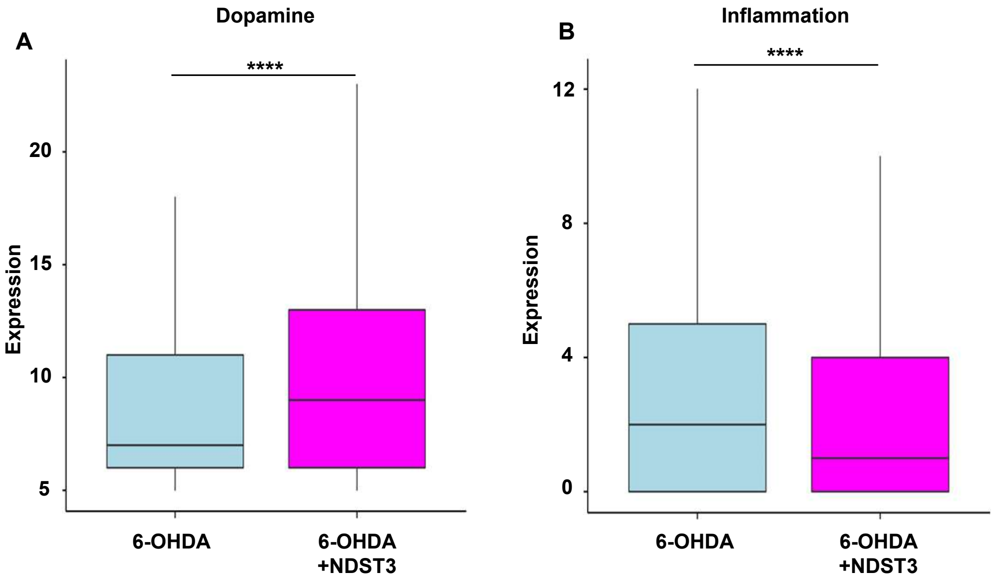

Figs S28. Expression level quantification of scRNA-seq result for factors that were validated in spatial RNA transcriptomics. (A) Box plot representing the quantification of dopamine related factors' expression level for 6-OHDA-induced PD model and NDST3-treated PD model, indicating statistical significance as ****$p < 0.0001$. (B) Box plot representing the quantification of inflammatory response related factors' expression level for 6-OHDA-induced PD model and NDST3-treated PD model, indicating statistical significance as ****$p < 0.0001$.

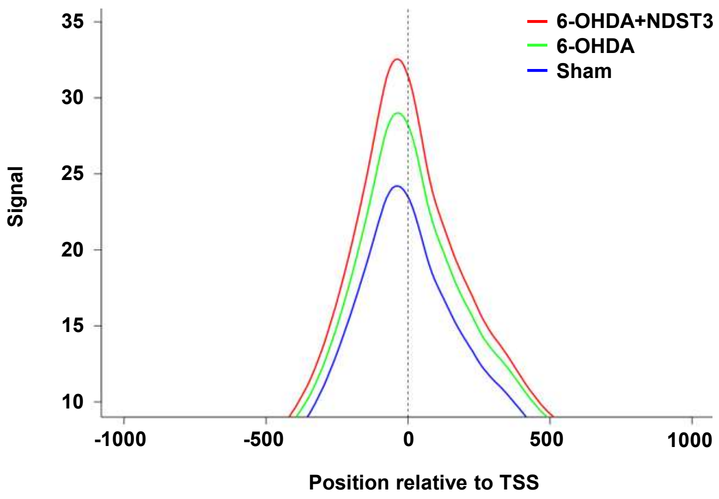

Figs S29. Metaplot showing the CUT&RUN signals at TSSs in sham, 6-OHDA-induced PD model, and NDST3-treated PD model.

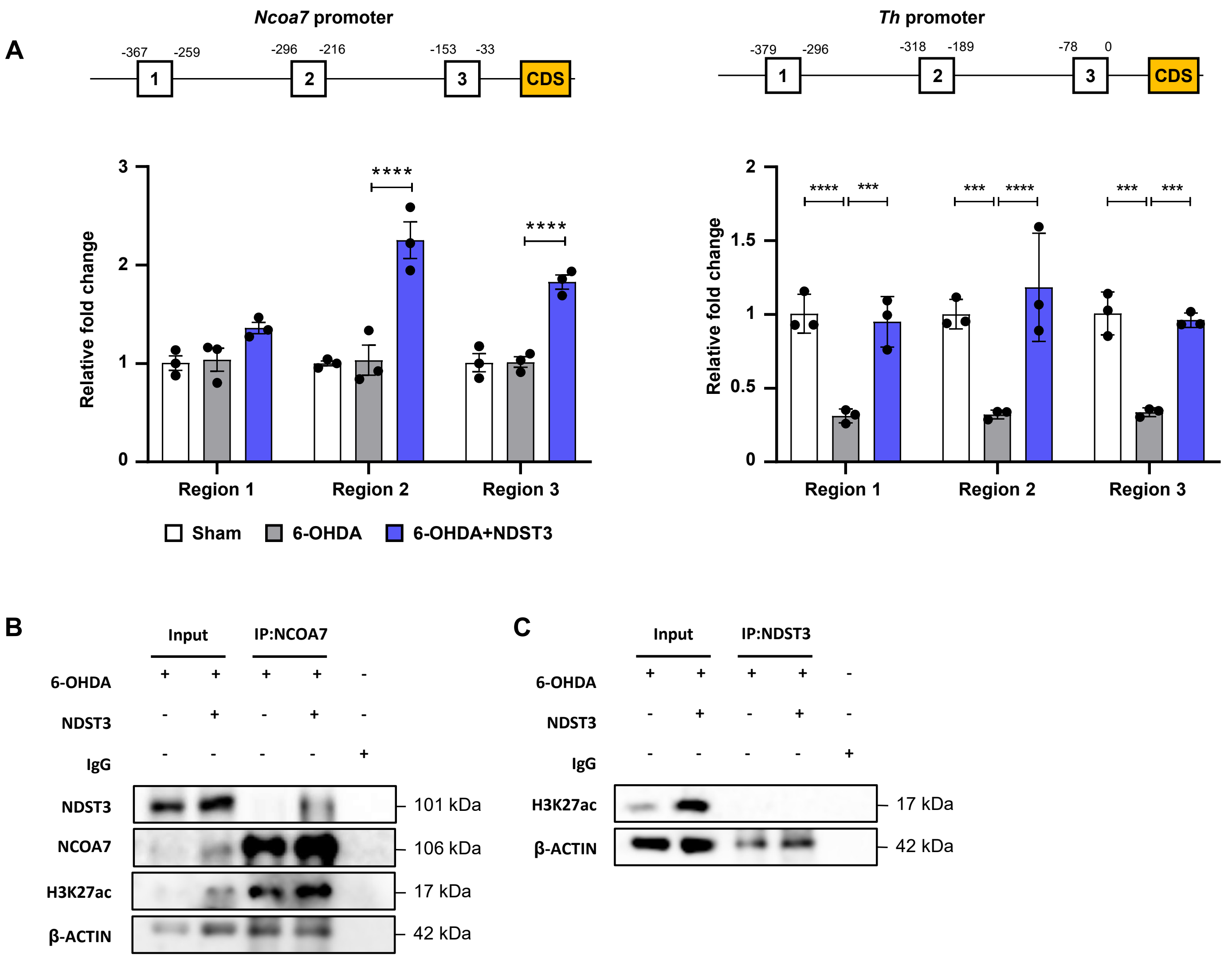

Figs S30. Confirmation of interaction among NDST3, NCOA7 and H3K27ac. (A) Enrichment of H3K27ac on chromatin regions of mouse *Ncoa7* and *Th* locus by ChIP-qPCR analysis in the primary dopaminergic neuron. Data represent mean ± SEM (n = 3 independent animal per group). One-way ANOVA with Tukey's multiple comparison test, ***p < 0.001 and ****p < 0.0001. (B-C) Co-immunoprecipitation assay for NCOA7 and NDST3. Identification of specific NDST3 (101 kDa), NCOA7 (106 kDa) and H3K27ac (17 kDa) proteins , with β-ACTIN serving as a loading control.

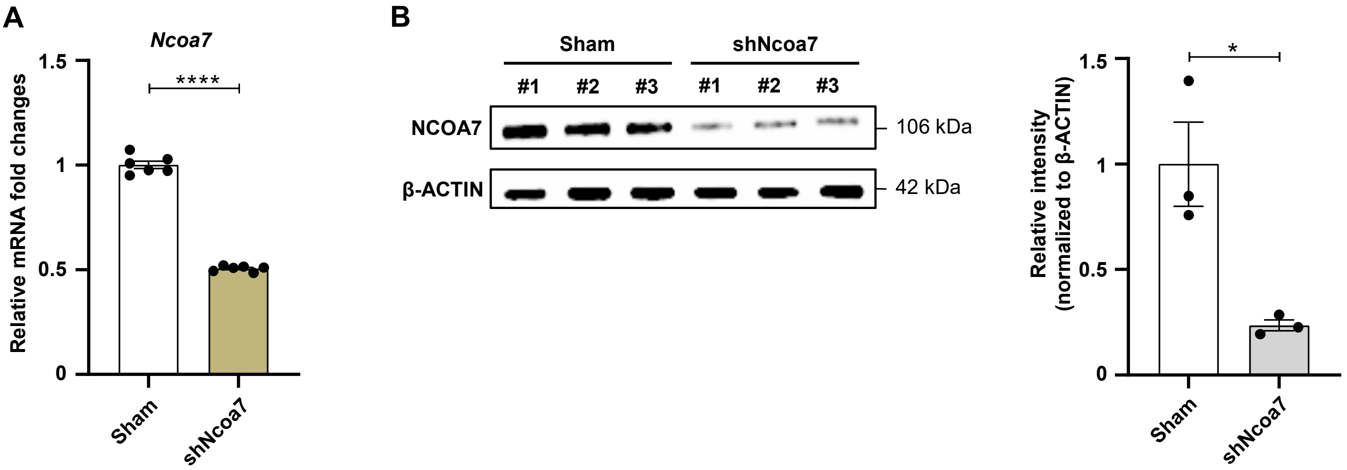

Figs S31. Confirmation of knockdown efficiency of Ncoa7 in the primary dopaminergic neuron. (A) Gene expression level of *Ncoa7* in the primary dopaminergic neuron with or without shNcoa7. Data are presented as mean ± SEM (n = 6 wells per group). Student T-test. ****$p < 0.0001$. (B) Immunoblot analysis showing NCOA7 expression in primary dopaminergic neuron lysates from sham, shNcoa7-treated group. Identification of specific bands for NCOA7 (106 kDa) proteins, with β-ACTIN serving as a loading control. Data are presented as mean ± SEM (n = 3 wells per group). Student T-test. *$p < 0.05$.

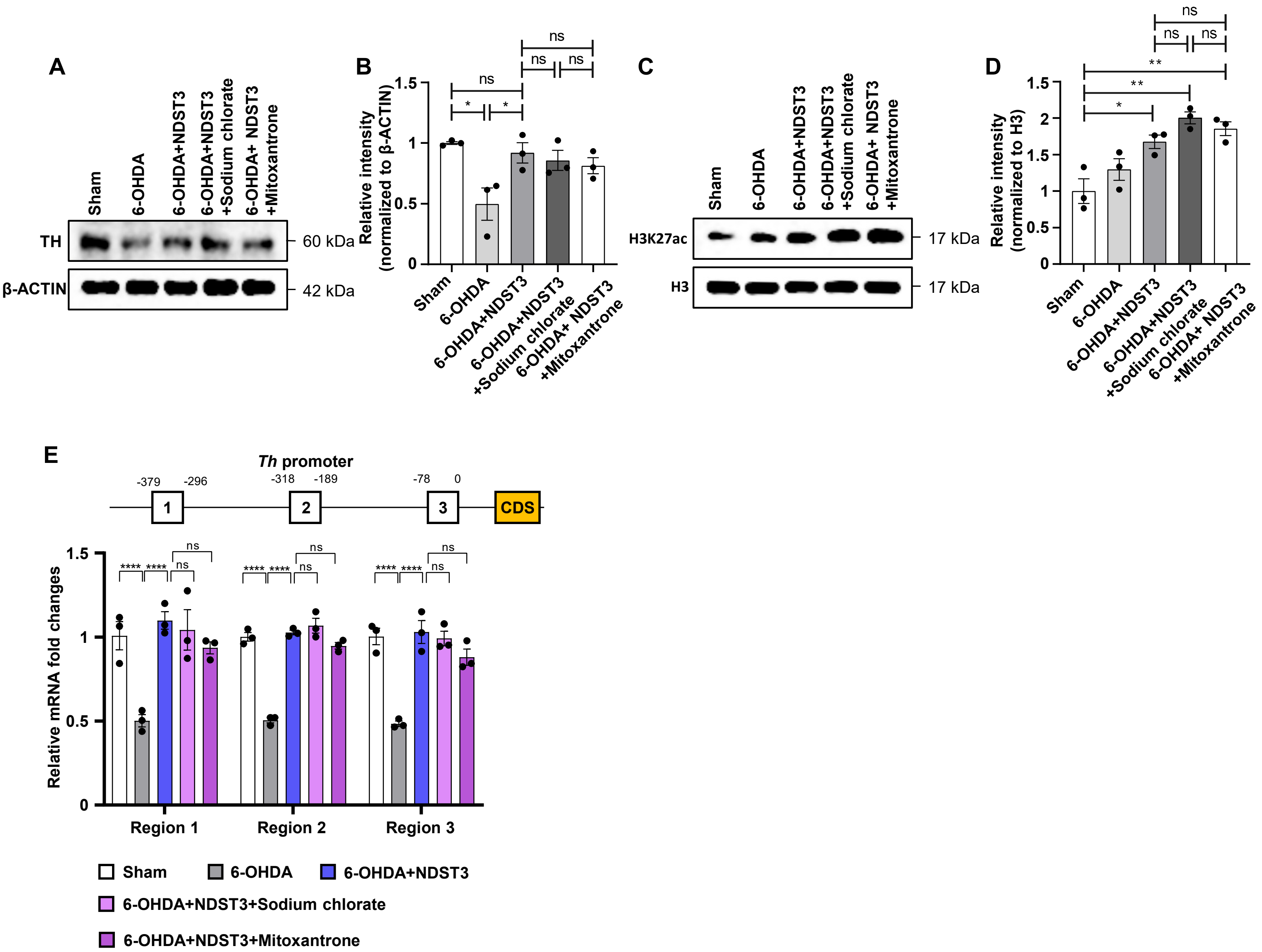

Figs S32. Confirmation of NDST3 enzymatic function in HS biosynthesis. (A) Immunoblot analysis showing TH expression in primary dopaminergic neuron lysates from NDST3-treated group with inhibitors targeting HS biosynthesis. Identification of specific bands for TH (60 kDa) proteins, with β-ACTIN serving as a loading control. (B) Quantification of the relative intensity of TH as shown in Fig S32A. Data are presented as mean ± SEM (n = 3 wells per group), One-way ANOVA with Tukey's multiple comparison test, $*p < 0.05$ and ns = not significant. (C) Immunoblot analysis showing H3K27ac expression in primary dopaminergic neuron lysates from NDST3-treated group with inhibitors targeting HS biosynthesis. Identification of specific bands for H3K27ac(17 kDa) proteins, with H3 serving as a loading control. (D) Quantification of the relative intensity of H3K27ac as shown in Fig S32C. Data are presented as mean ± SEM (n = 3 wells per group). One-way ANOVA with Tukey's multiple comparison test, $*p < 0.05$, $**p < 0.01$ and ns = not significant. (E) Enrichment of H3K27ac on chromatin regions of mouse *Th* locus by ChIP-qPCR analysis in the primary dopaminergic neuron. Data represent mean ± SEM (n = 3 wells per group). One-way ANOVA with Tukey's multiple comparison test, $****p < 0.0001$ and ns = not significant.

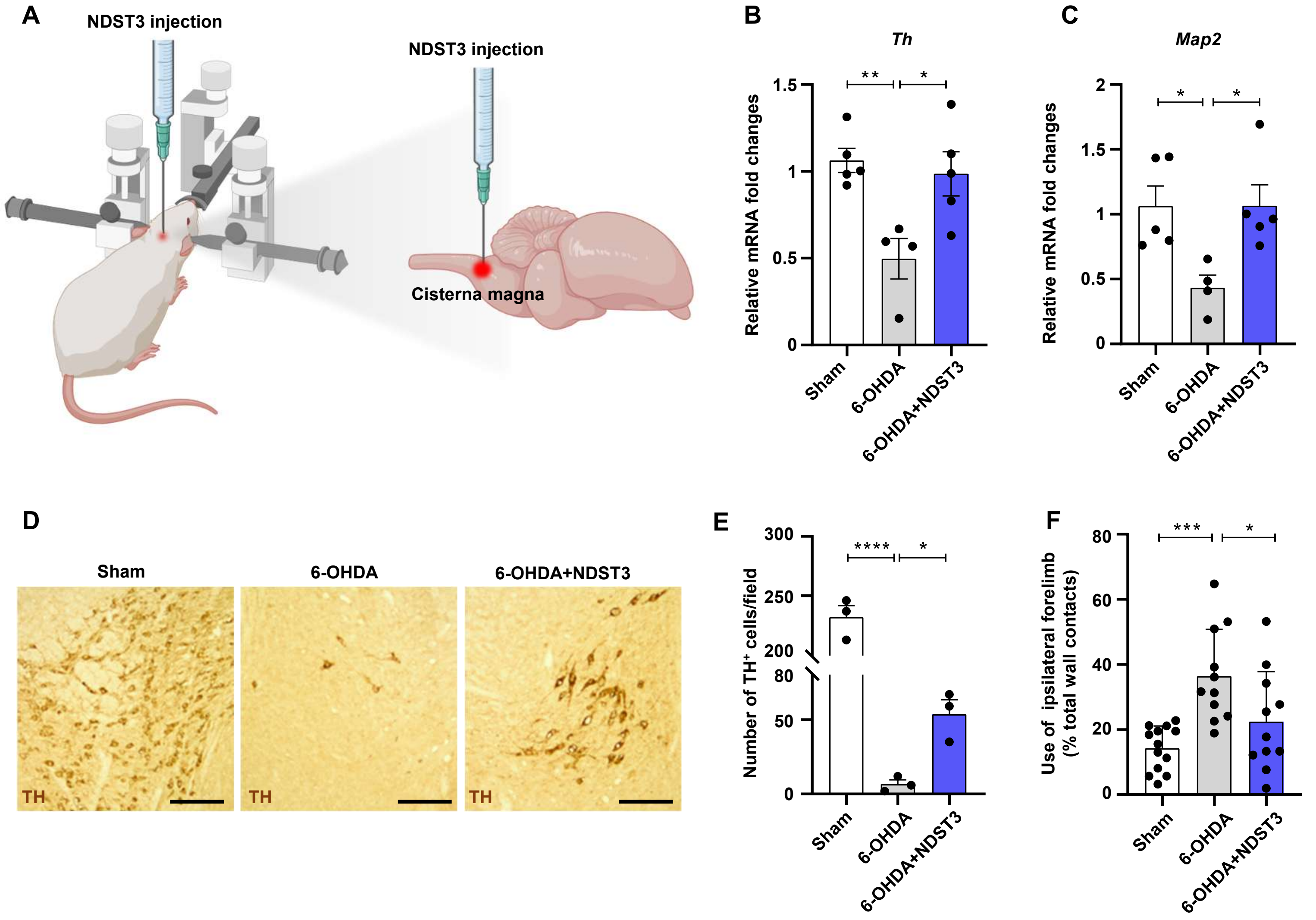

Figs S33. Confirmation of efficacy of NDST3 injected through intra-cisterna magna. (A) Schematic diagram of intra-cisterna magna injection method. Schematic illustration created with Biorender.com. (B-C) Relative mRNA expression analysis of mature DA neuronal markers. Data are presented as mean ± SEM (n = 4 – 5 independent animal per group). One-way ANOVA with Tukey's multiple comparisons test. $*p < 0.05$, and $**p < 0.01$. (D) Representative image of DAB-TH staining in SN, with (E) the corresponding quantitative analysis. Data are presented as mean ± SEM (n = 3 animal per group). One-way ANOVA with Tukey's multiple comparisons test. $*p < 0.05$, and $****p < 0.0001$. (F) % of use for ipsilateral forelimb in sham, 6-OHDA-induced PD model, and NDST3-treated PD model. Data are presented as mean ± SEM (n = 11 – 13 independent animal per group). One-way ANOVA with Tukey's multiple comparisons test. $*p < 0.05$, and $***p < 0.001$.

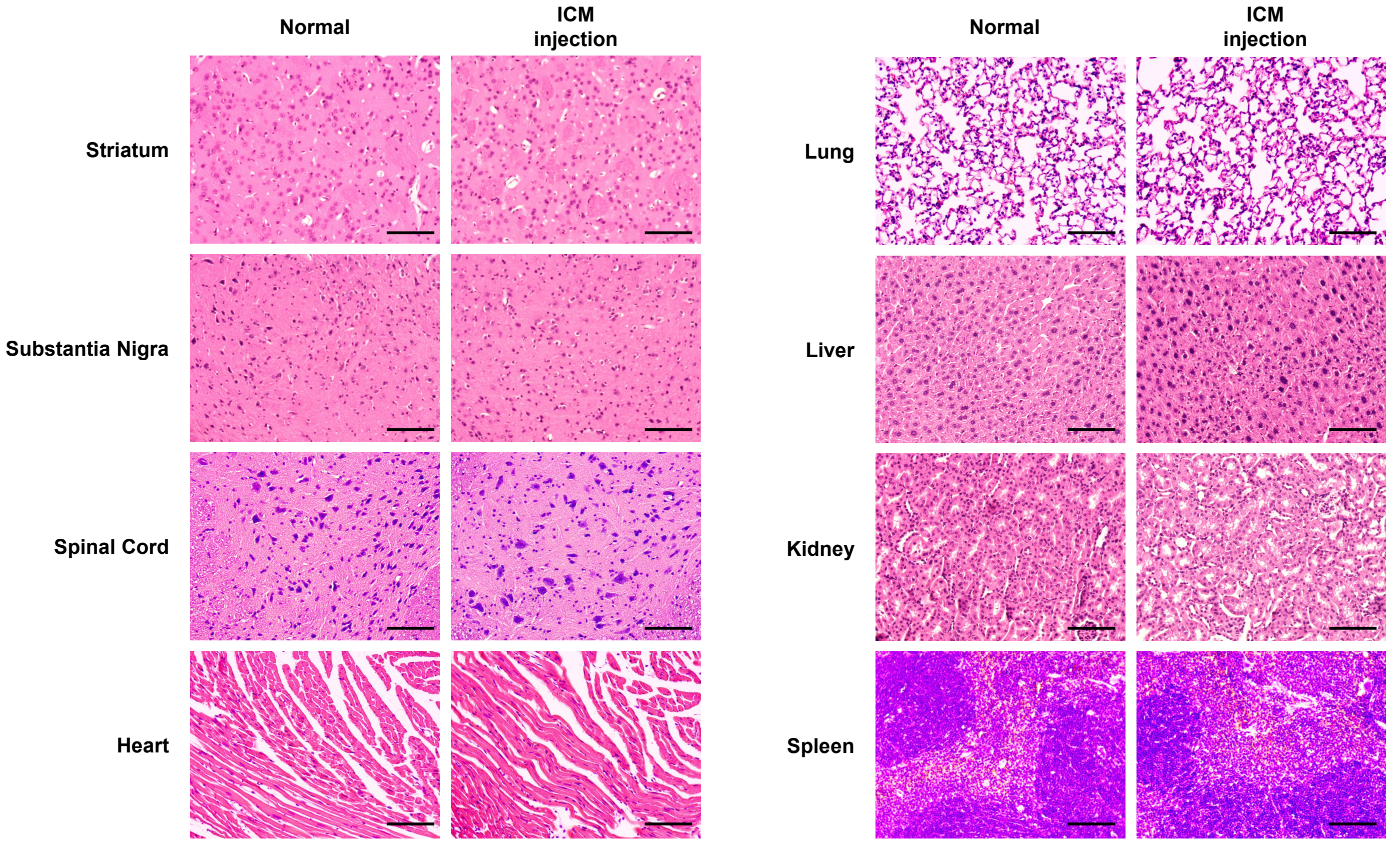

Figs S34. Hematoxylin and eosin (H&E) staining of major organs after Lenti-NDST3 administration through intra-cisterna magna. Scale bar = 500μm.

Statistics	X^2	P value	Difference (Bonferroni's pairwise comparison)
Whole data	13.275385	0.003766**	Sham - 6-OHDA<br>6-OHDA – 6-OHDA+NDST3
Full AP vs. No AP	9.394737	0.028571*	Sham - 6-OHDA<br>6-OHDA – 6-OHDA+NDST3
Full AP vs. incomplete AP	9.670979	0.008731**	Sham - 6-OHDA<br>6-OHDA – 6-OHDA+NDST3

Table 1. Chi-Square test results according to detection frequencies of full AP, incomplete AP and No AP of the research groups (Sham, 6-OHDA-induced PD model, and NDST3-treated PD model). *$p < 0.05$ and **$p < 0.01$.

Motif name	Fold enrichment	Consensus sequence	Motif
Etv4	267.08	NCAGGAAGNN	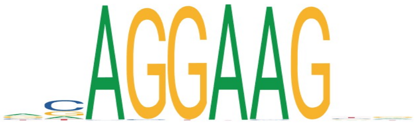
Emx2	229.52	NNTAATTAGN	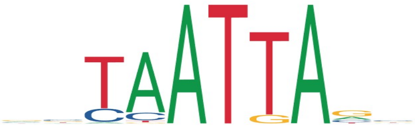
Emx1	227.73	NCTAATTANN	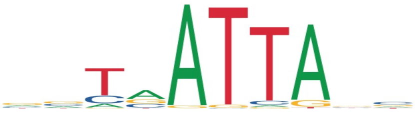
Foxa3	227.13	NNGTAAACANN	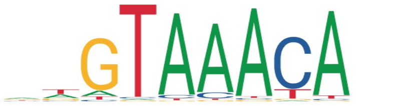
Foxk2	227.13	NNGTAAACANN	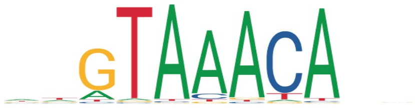
Evx1	219.98	NNTAATTANC	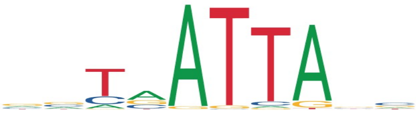
Atoh1	205.08	NNCAGATGGNN	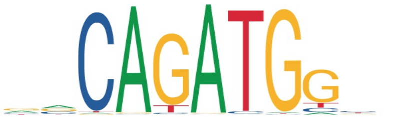
Dlx1	185.10	NNTAATTANN	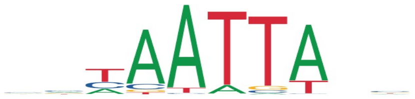
Evx2	185.10	NNTAATTANN	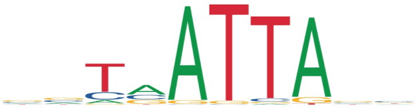
Barhl1	170.05	NNTAATTGNN	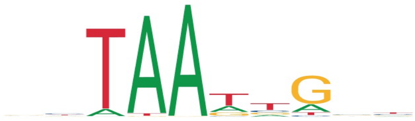

Table 2. Transcription factor motif prediction enriched in the CUN&RUN.
