# Supplementary figures and images for: NDST3‐Induced Epigenetic Reprogramming Reverses Neurodegeneration in Parkinson's Disease
Source: Adv Sci (Weinh). 2025 Nov 21;13(14):e07323. doi: 10.1002/advs.202507323 (PMC12970244; doi:10.1002/advs.202507323)

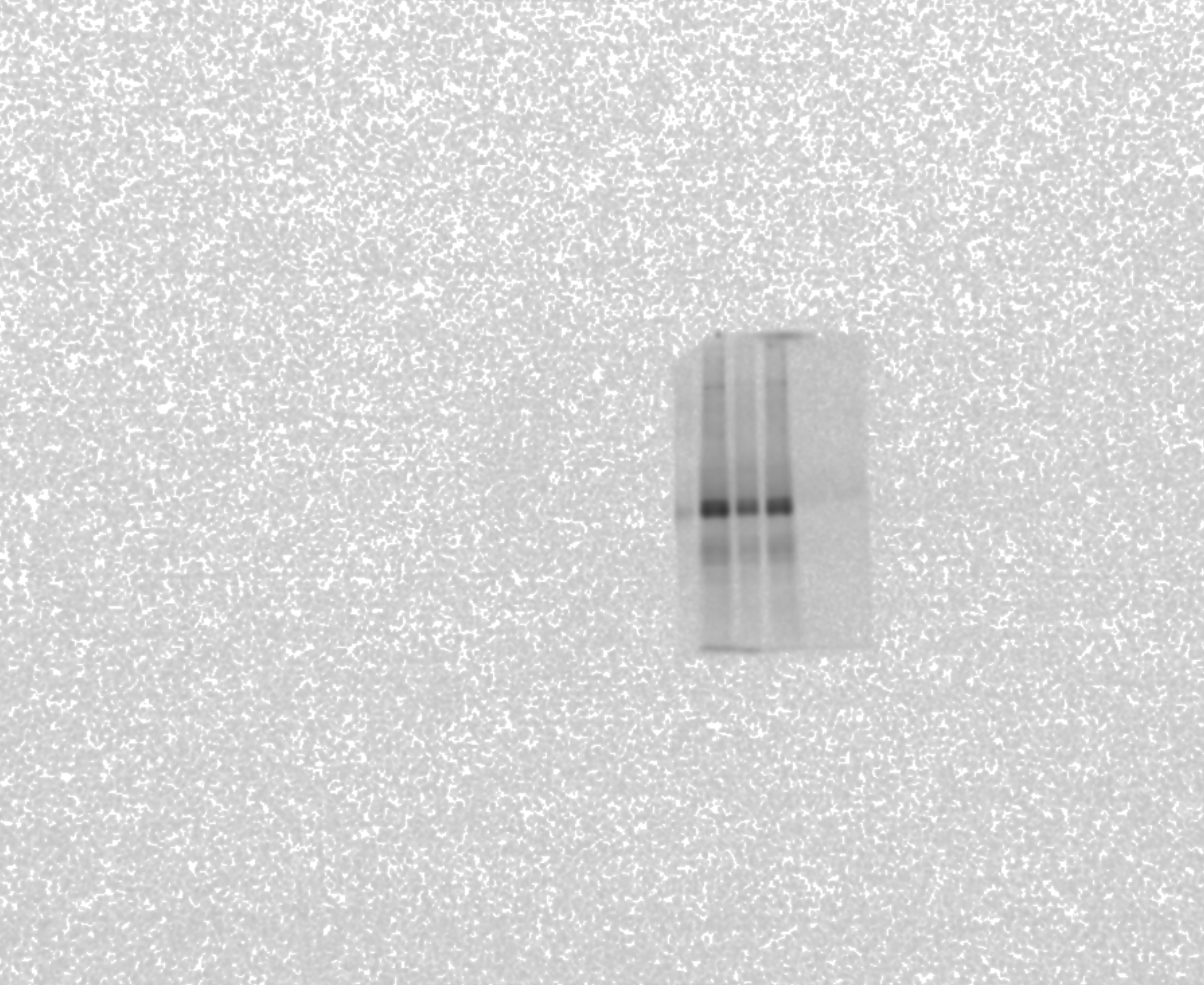

Supplement: Supplementary file 2 — Supporting Information [file ADVS-13-e07323-s002.zip › S.Fig. 5C_SYN1.tif]

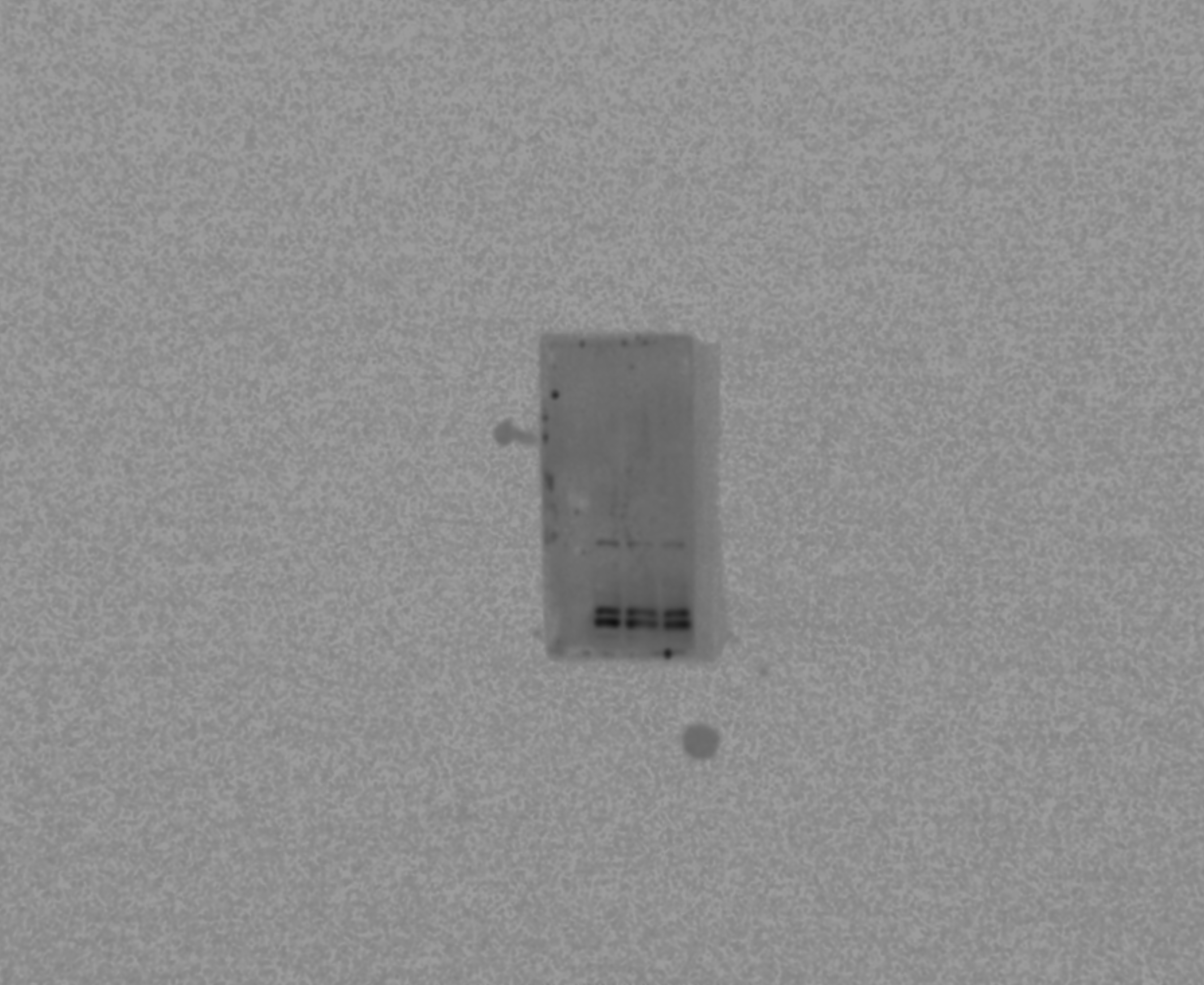

Supplement: Supplementary file 2 — Supporting Information [file ADVS-13-e07323-s002.zip › S.Fig. 5C_Beta-actin.tif]

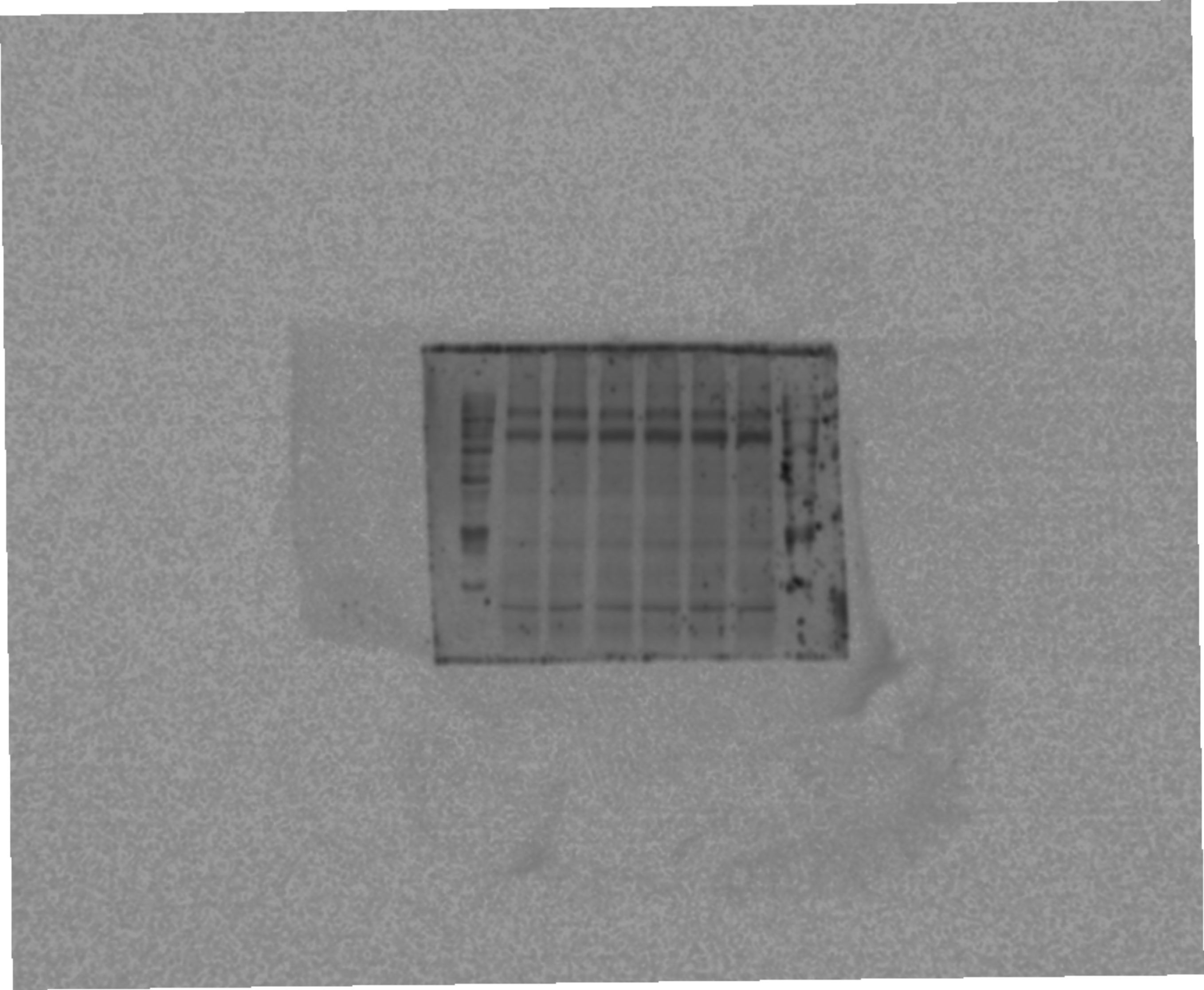

Supplement: Supplementary file 2 — Supporting Information [file ADVS-13-e07323-s002.zip › S.Fig. 5A_NDST3.tif]

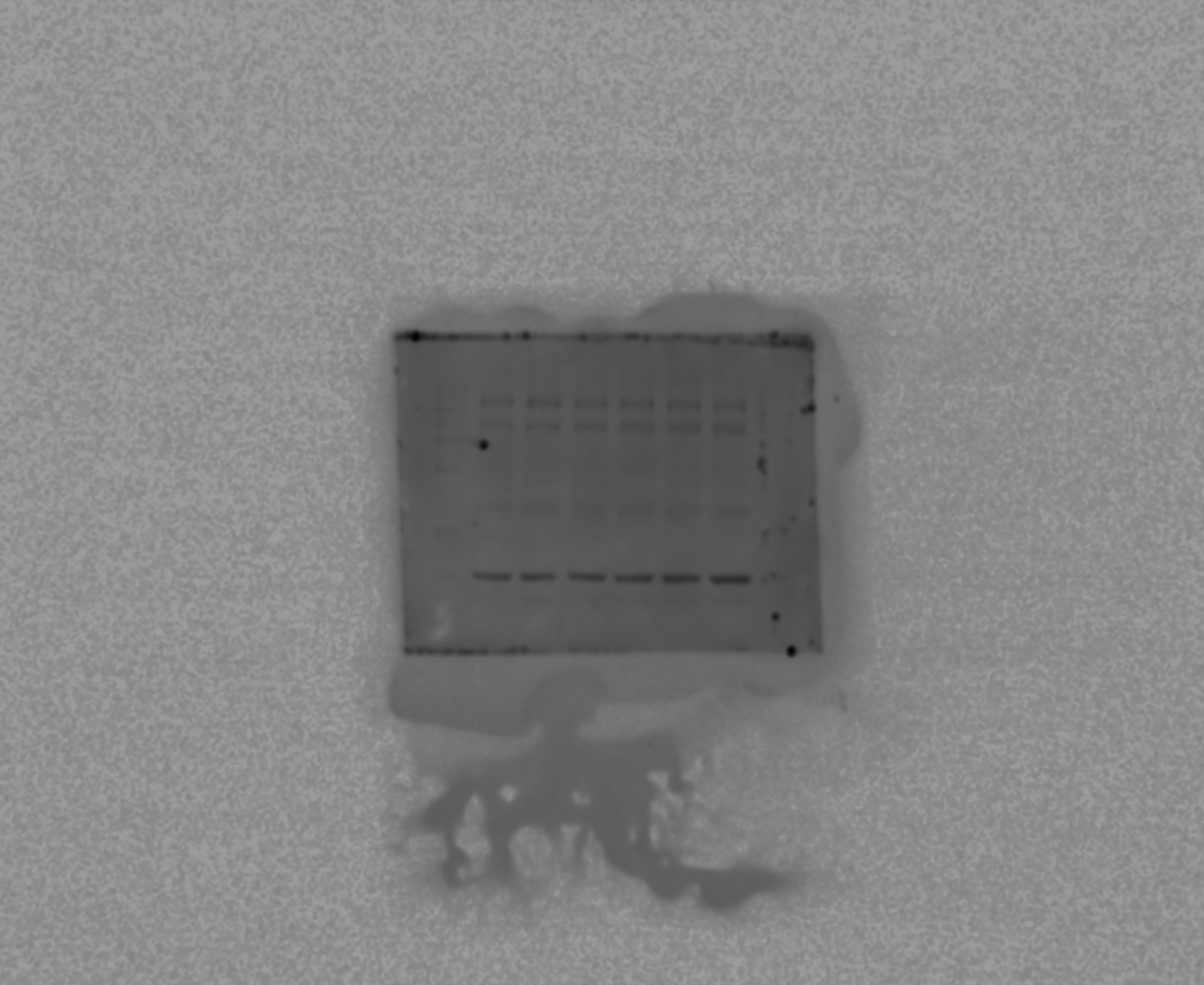

Supplement: Supplementary file 2 — Supporting Information [file ADVS-13-e07323-s002.zip › S.Fig. 5A_Beta actin.tif]

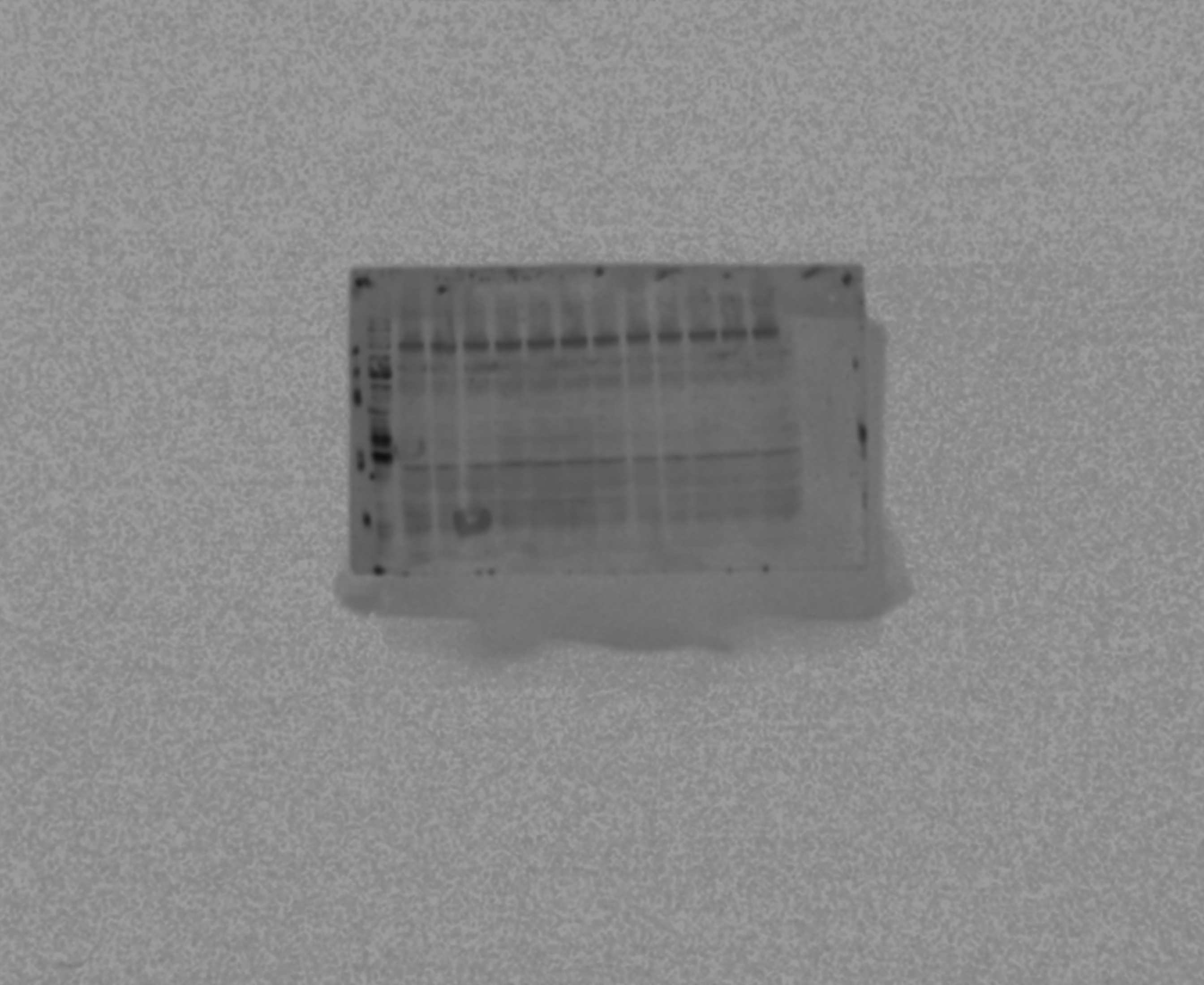

Supplement: Supplementary file 2 — Supporting Information [file ADVS-13-e07323-s002.zip › S.Fig. 3C_NDST3.tif]

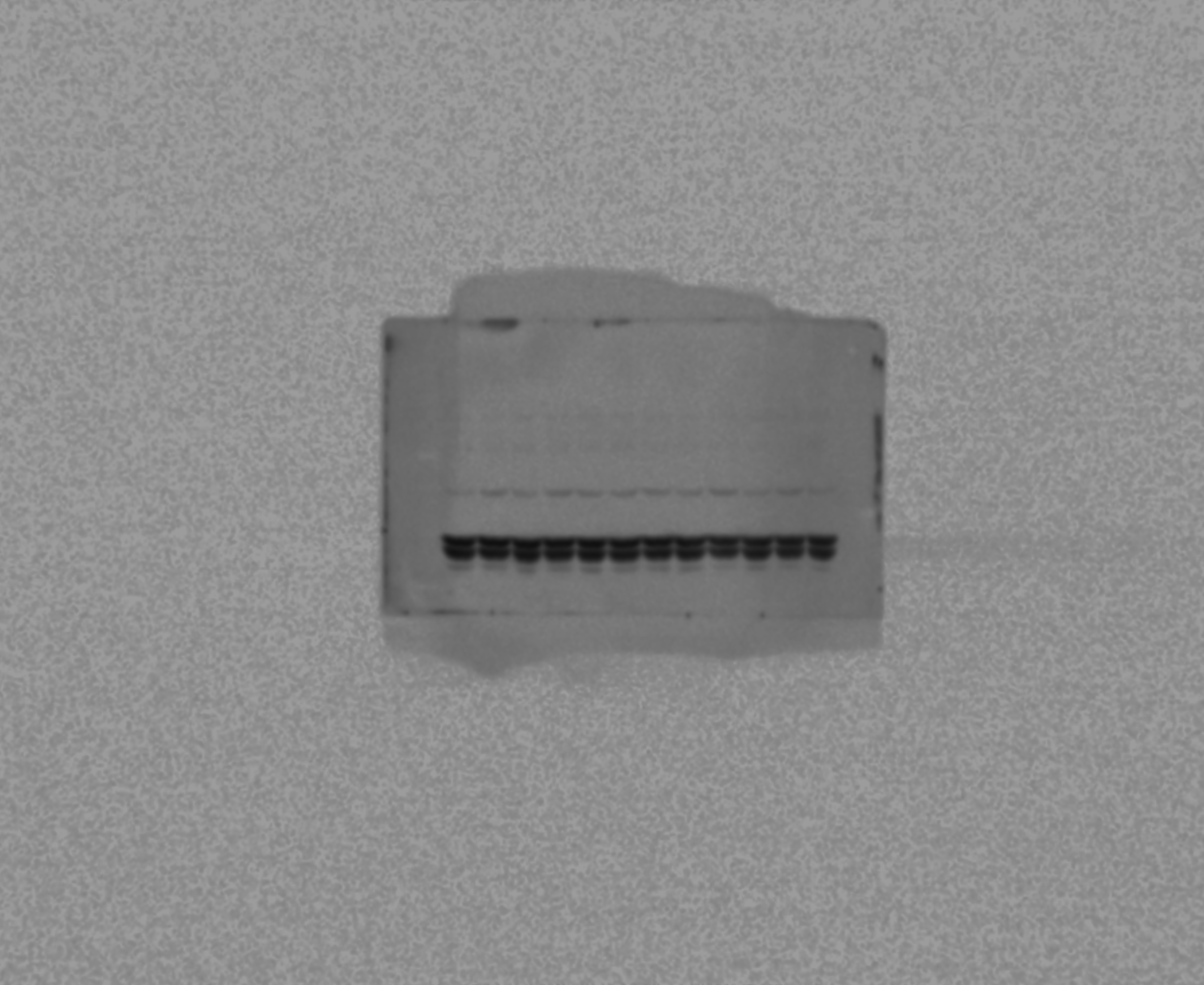

Supplement: Supplementary file 2 — Supporting Information [file ADVS-13-e07323-s002.zip › S.Fig. 3C_Beta actin.tif]

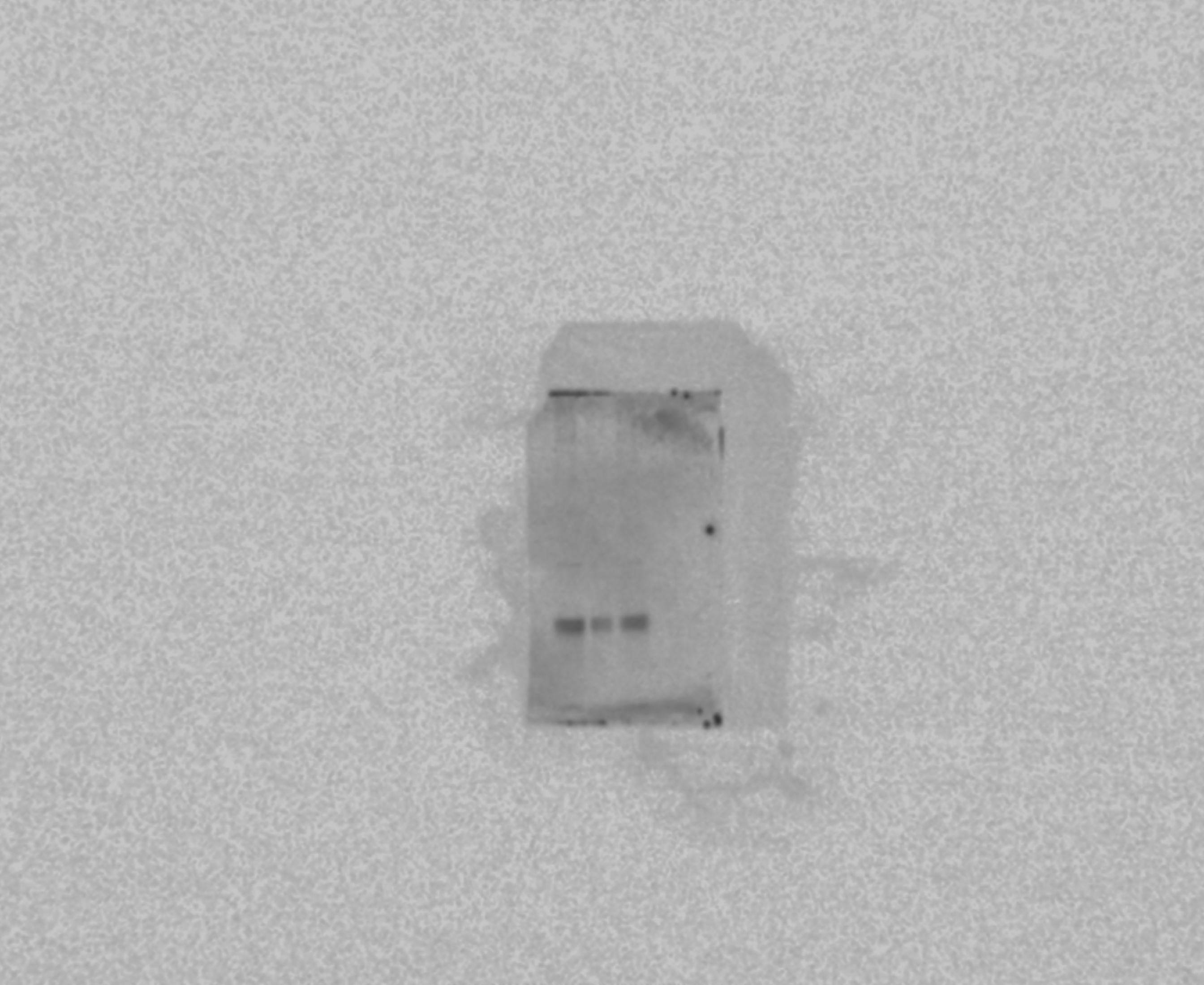

Supplement: Supplementary file 2 — Supporting Information [file ADVS-13-e07323-s002.zip › S.Fig. 5C_TH.tif]
